# Measurement tools and outcome measures used in transitional patient safety; a systematic review

**DOI:** 10.1371/journal.pone.0197312

**Published:** 2018-06-04

**Authors:** Marije A. van Melle, Henk F. van Stel, Judith M. Poldervaart, Niek J. de Wit, Dorien L. M. Zwart

**Affiliations:** Julius Center for Health Sciences and Primary care, University Medical Center Utrecht, Utrecht, the Netherlands; University of Washington, UNITED STATES

## Abstract

**Background:**

Patients are at risk for harm when treated simultaneously by healthcare providers from different healthcare organisations. To assess current practice and improvements of transitional patient safety, valid measurement tools are needed.

**Aim and methods:**

To identify and appraise all measurement tools and outcomes that measure aspects of transitional patient safety, PubMed, Cinahl, Embase and Psychinfo were systematically searched. Two researchers performed the title and abstract and full-text selection. First, publications about validation of measurement tools were appraised for quality following COSMIN criteria. Second, we inventoried all measurement tools and outcome measures found in our search that assessed current transitional patient safety or the effect of interventions targeting transitional patient safety.

**Results:**

The initial search yielded 8288 studies, of which 18 assessed validity of measurement tools of different aspects of transitional safety, and 191 assessed current transitional patient safety or effect of interventions. In the validated measurement tools, the overall quality of content and structural validity was acceptable; other COSMIN criteria, such as reliability, measurement error and responsiveness, were mostly poor or not reported. In our outcome inventory, the most frequently used validated outcome measure was the Care Transition Measure (n = 9). The most frequently used non-validated outcome measures were: medication discrepancies (n = 98), hospital readmissions (n = 55), adverse events (n = 34), emergency department visits (n = 33), (mental or physical) health status (n = 28), quality and timeliness of discharge summary, and patient satisfaction (n = 23).

**Conclusions:**

Although no validated measures exist that assess all aspects of transitional patient safety, we found validated measurement tools on specific aspects. Reporting of validity of transitional measurement tools was incomplete. Numerous outcome measures with unknown measurement properties are used in current studies on safety of care transitions, which makes interpretation or comparison of their results uncertain.

## Background

Incidents frequently occur when patients transfer between different healthcare levels [1], so improving patient safety during healthcare transitions, i.e. transitional patient safety, is an important objective in healthcare. Recently, international patient safety experts have called to widen the safety scope across multiple healthcare levels. Accordingly, the need arises for valid and reliable measurement tools for transitional patient safety [2], because to improve transitional patient safety, we need to know the current status and effect of transitional safety interventions.

In measuring patient safety, we can be guided by the three dimensions of measuring quality of care developed by Donabedian et al, namely *structure* (how care is organised), *process* (what healthcare professionals (HCP) do to maintain or improve health, either for healthy people or for those diagnosed with a healthcare condition) and *outcome* (what ultimately happens to the patient) [3]. Later, Pronovost et al. expanded the quality of care model by adding patient safety culture as a precondition for the provision of high quality care [4].

**Fig 1:**
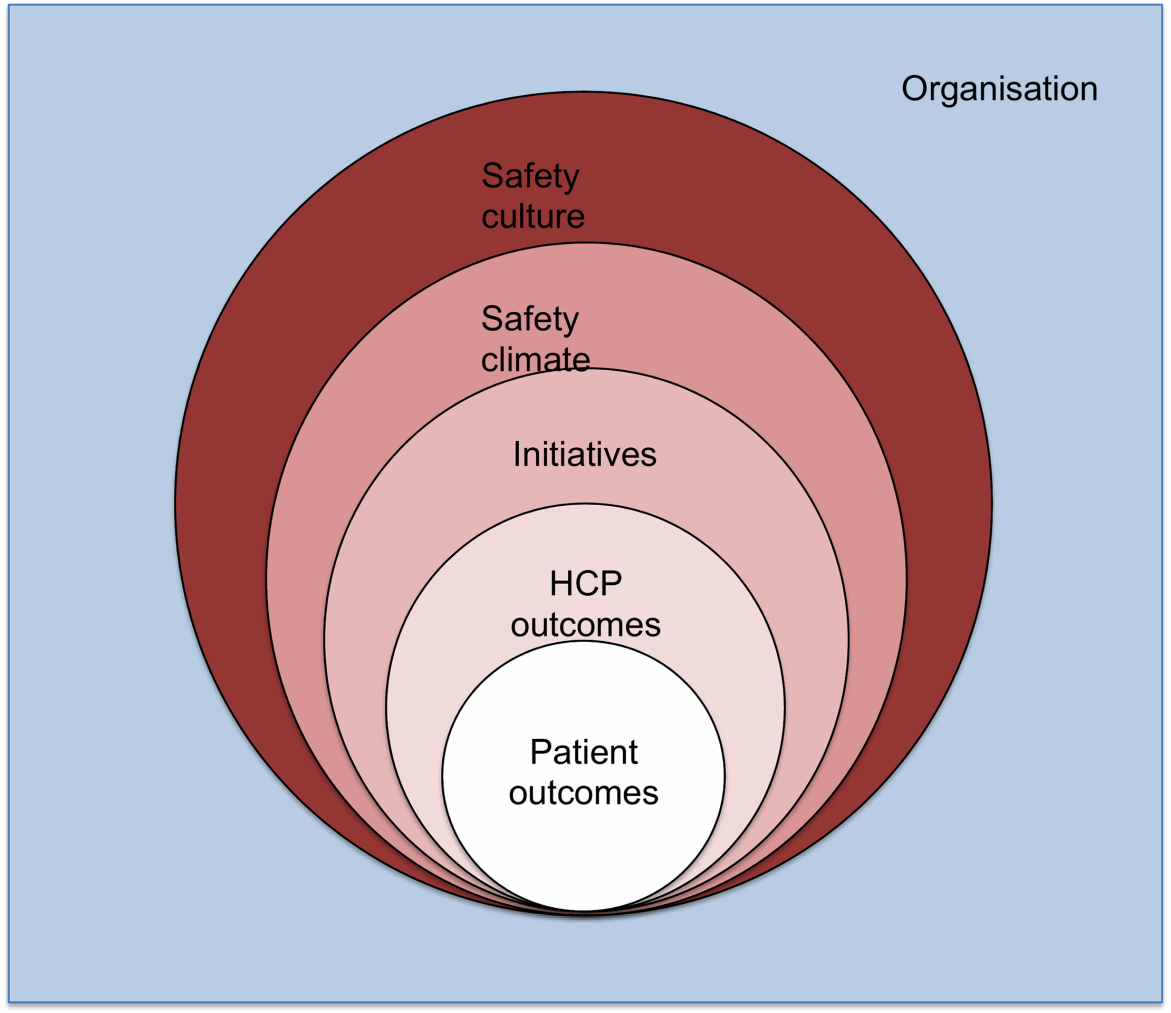
The culture-behaviour-patient outcome continuum in patient safety. HCP = healthcare professional [5].

This extended model fits in the ‘culture-behaviour-outcomes’ continuum as presented by the Health Foundation (Fig 1) [5]. This continuum covers the different types of safety outcomes: safety culture, climate, initiatives and outcomes. Safety culture is a broad term representing the organisation’s values and actions related to safety, whereas safety climate focuses on perceptions of professionals about the way in which safety is managed in the organisation [5]. Initiatives are the actual improvements in the organization that are developed and implemented to improve patient safety. Outcome is further specified and encompasses HCP and patient outcomes. HCP outcomes comprise staff behaviour and transitional patient safety incident reporting (Fig 1). All levels are supposed to interact with each other [5].

To get an overall view, transitional patient safety should be measured from both the perspective of the HC and the patient (Fig 1), as we know patients have a different perspective on patient safety then HCPs and both views complement each other [6]. HCPs do oversee the process of care, but only within their own organisation. The patient experiences the entire journey across different healthcare levels, but does not oversee the organisational aspects [7, 8]. Therefore, instruments measuring both the perception of the patient and the HCP on transitional patient safety are needed.

Previous research on measurement of transitional patient safety covered either a single transition, such as discharge or referral, or related concepts like “continuity of care” [9–15]. However, the patient’s journey often entails several transitions per episode of illness. Focusing only on a single transition does not appreciate the complex reality of multiple communications, transitions and handovers between the different healthcare professionals and settings involved. In contrast, the domain of ‘continuity of care’ is broader than that of transitional patient safety. Transitional patient safety focusses on the prevention or reduction of harm associated with patient’s care transitions, while continuity of care covers other quality aspects like efficiency, effectiveness, equity, and timeliness [16]. Additionally, continuity of care is primarily studied within one organisation, while transitional patient safety focuses on safety between distinct healthcare settings.

At present there is no overview of the available instruments for measuring transitional patient safety, and their validity and reliability. Our primary aim was to systematically identify all validated measurement tools measuring transitional patient safety and appraise their quality. In this, we focus on transitional patient safety between primary care practice (PCP) and hospital comprising all transitions between hospital and PCP (i.e. discharge, referral and concomitant care at PCP and hospital outpatient settings). Our secondary aim was to see whether these validated measurement tools were actually used to measure transitional patient safety outcomes and explore all other (non validated) outcomes used to measure safety of care transitions in the current literature.

## Methods

### Search strategy and data sources

We conducted a systematic literature search in Pubmed (including MEDLINE), EMBASE, PsychINFO and CINAHL (January 1^st^ 2017). We used a combination of the following search strategies: 1) transitional care, including various terms and keywords synonymous and related to transitional care, i.e. continuity of care, transitional, cross-boundary, seamless, integrated; 2) measurement, for which we used an existing filter by Terwee et al. [17, 18]; and 3) patient safety, for which we used an existing filter by Tanon et al. [19]. Both filters were adjusted for PsychInfo. Detailed search strategies are available online in S1 Text. We also reviewed the reference lists of the identified studies in both the validity assessment and the outcome inventory and checked previously published systematic reviews on the measurement of and interventions on related constructs identified in a purposeful Medline and Prospero database search [9–15, 20–26].

The systematic review was registered in the PROSPERO database (nr 42016037311). Additionally, a PRISMA Checklist has been added (S1 Prisma Checklist)

### Study selection

Electronic citations, including available abstracts, were independently screened by two researchers (JP and MM). If the title and abstract did not clearly indicate whether the inclusion criteria were met, full-text was obtained and reviewed. For our primary aim we included studies that assessed the quality of validated measurement tools on aspects of transitional patient safety (hereinafter referred to as “validity assessment”. For our secondary aim we additionally included studies reporting the effect of interventions to improve or evaluating aspects of transitional patient safety in a cross-sectional design (hereinafter referred to as “outcome inventory”). For both aims we included all quantitative studies focusing on the transition between primary and hospital care, addressing all safety outcome types in patient safety according to the Health Foundation [5], from both the patients’ as well as the professionals’ perspectives. No limits were set on the design of the study or on the target population (patients and HCPs). Studies addressing organisational concepts like integrated care were excluded (S2 Text). The full-text publications were reviewed by two independent reviewers (JP and MM) using the same in- and exclusion criteria. If necessary, a third reviewer (HS) was consulted for a final decision.

### Data extraction

#### Validity assessment

Data extraction, and the assessment of measurement properties and methodology were performed by two reviewers (HS and MM) independently. Consensus was reached in consensus meetings. Data were collected on the characteristics of the included publications, namely country and language of development, type of transition, safety outcome type according to the culture-behaviour-outcome continuum, the construct that was measured, the target population (including mean age and gender, number of respondents and response rate), the number of items, and dimensions after factor analysis. Transitions were divided into three categories: from primary care to hospital (referral or admission), from hospital to primary care (discharge after hospital admission or outpatient clinic visit) and two-way transitions (admission and discharge, overall continuity and simultaneous care at hospital outpatient clinic and primary care practice).

The methodological quality of studies was assessed according to the “COnsensus-based Standards for the selection of health Measurement INstruments” (COSMIN) criteria [27]. This checklist evaluates internal consistency, reliability, measurement error, content validity, structural validity, hypothesis testing, cross-cultural validity, criterion validity, responsiveness, interpretability, and generalizability. Each item was rated on a 4-point scale (poor, fair, good or excellent). The overall score for each criterion was determined by taking the lowest rating of the items. As no optimal reference standard is present in transitional patient safety, many items of the COSMIN were not applicable. Therefore, we excluded Box G (cross-cultural validity), Box H (criterion validity) and items D3, F7, F8 from the assessment. As Box C (measurement error) and Box I (responsiveness) were never assessed in all included publications, and therefore not reported.

#### Outcome inventory

Data extraction was performed by MM, and data was collected on year, country and language of development, type of transition, safety outcome type according to the culture-behaviour-outcome continuum, measured construct, target population, study design, perception, whether a validated measurement tool was used and other outcomes used to assess current state or improvement of transitional patient safety. As in some studies multiple outcomes were assessed, each study could describe multiple levels of outcomes and perceptions. Our secondary aim was to see if the validated measurement tools from the validity assessment were actually used and map other outcome measurements used in literature. Because we did not aim to assess possible intervention effects, we did not perform several topics from the PRISMA checklist assess the risk of bias of this second group of articles such as a quality appraisal and assessing the risk of bias [28].

## Results

**Fig 2:**
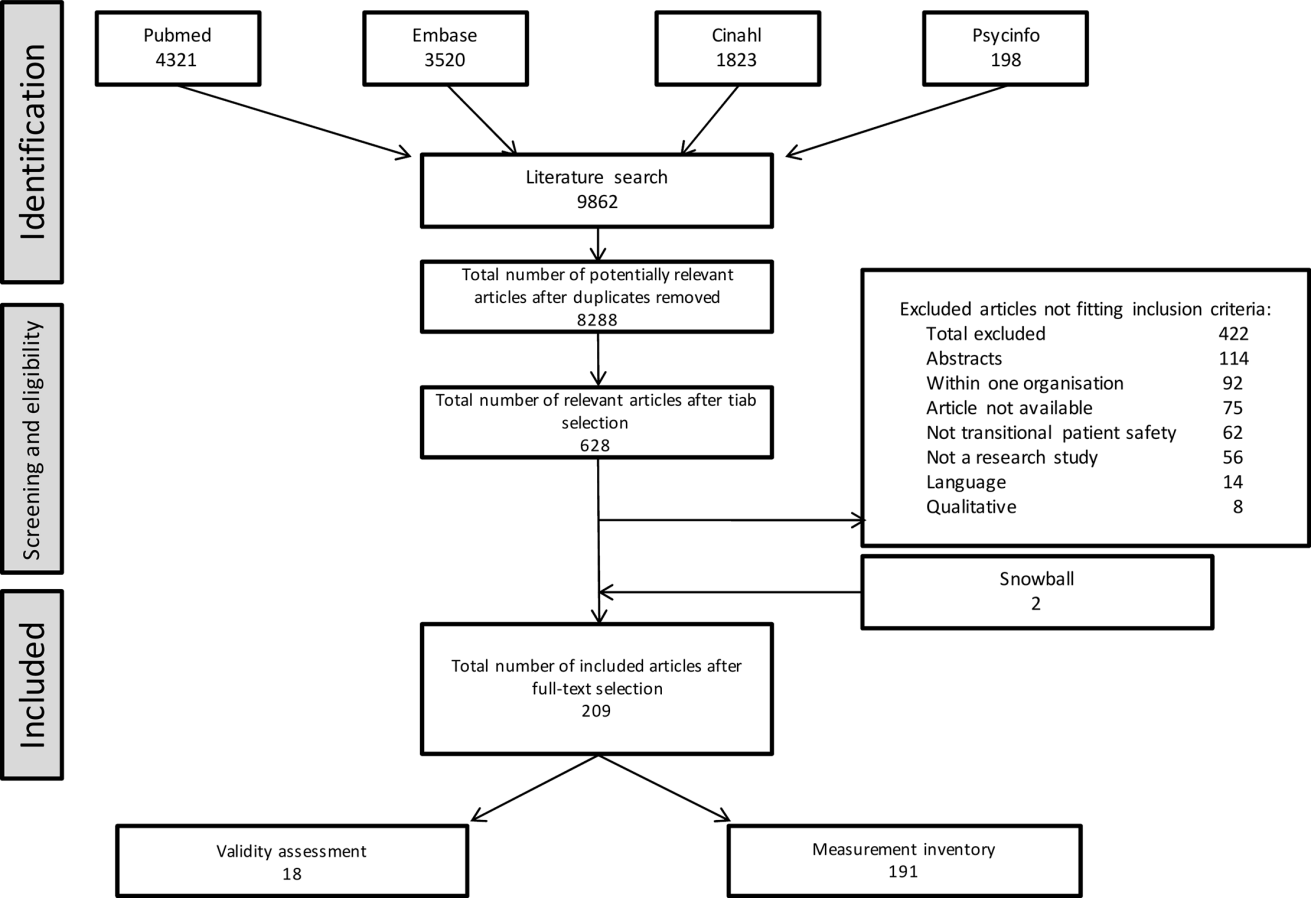
Flowchart of article selection procedure.

Our search identified 8288 unique, potentially relevant published scientific papers, of which 628 were selected for full-text selection (Fig 2). From these, 421 papers were excluded, because they did not meet inclusion criteria, the paper could not be found or did not report original research. Reference lists of included publication and related literature rendered two more publications on validated measurement tools. In total 209 publications remained, of which 18 concerned publications validating instruments measuring (aspects of) transitional patient safety and 191 publications concerned studies reporting either the current state of transitional patient safety or the effect of an intervention to improve safety of care transitions.

### Characteristics of studies in validity assessment

Table 1 presents the characteristics of the 18 included studies validating measurement tools on aspects of transitional patient safety; 12 studies considered eight distinct questionnaires measuring transitional patient safety from a patient’s perspective and six studies measured transitional patient safety from a HCP’s perspective [29–46]. Six measurement tools involved all transitions between PCP and hospital, six concerned only hospital discharge and three only referrals. Considering the safety outcome type measured, three measurement tools measured (aspects of) safety climate, 12 measured HCP outcomes, and five measured patient outcomes. All but two validated measurement tools were questionnaires. Most patient-reported questionnaires were targeted at adult or elderly patients. A complete list of all characteristics is available online (S1 Table).

**Table 1:** Characteristics of the publications in the validity assessment of measurement tools on aspects of transitional patient safety. Level of outcome = the level of outcome in culture-behaviour-outcome continuum.

Authors	N	Country	Measurement tool	Transition	Safety outcome type	Subject of questionnaire	Target population	Response rate
			**Patient perspective**					
**Aller** **2013**	1500	SP	Continuity of care between care Levels (CCAENA)	Hospital<->PCP	HCP outcome	Continuity of care	Patients that have experienced a transition	23%
**Berendsen** **2009**	1404	NL	Consumer Quality Index Continuum of Care (CQI-COC)	PCP referral and relational continuity	HCP outcome	Collaboration between PCP and hospital	Referred patients	65%
**Coleman** **2002**	60	USA	Care Transition Measure (CTM)	Discharge from hospital to home/nursing home	HCP outcome	Care transition	Patients ≥65 years who were recently discharged from hospital and received subsequent skilled nursing care in facility/home.	NR
**Coleman** **2005**	200	USA	Care Transition Measure (CTM)	Discharge from hospital to home/nursing home	HCP and patient outcome	Care transition	Adult patients discharged with primary diagnosis of chronic obstructive pulmonary disease, congestive heart failure, stroke, or hip fracture	100%
**Graumlich** **2008**	460	USA	B-prepared	Hospital discharge	HCP outcome	Patient preparedness on hospital discharge	All adult patients discharged by internal medicine hospitalists	NR
**Grimmer** **2001**	500 (patients), 431 (carers)	AUS	PREPARED	Hospital discharge	HCP and patient outcome	Discharge	Patients ≥65 years, recently discharged from hospital	60% (patients), 52% (care givers)
**Hadjistavropoulos 2008**	204	CAN	Patient continuity of care Questionnaire (PCCQ)	Hospital discharge	HCP outcome	Continuity of care at discharge	Adult patients recently discharged from hospital	NR
**Haggerty** **2011**	236–427	CAN	4 questionnaires with dimension management continuity: PCAS, PCAT-S, CPCI, VANOCSS	PCP<-> other specialists	HCP outcome	Management continuity	Primary care patients who had seen more than one provider in the previous month	54–99%*
**Haggerty** **2012**	256	CAN	Patient Perceived Continuity from Multiple Clinicians	Hos<->PCP	HCP and patient outcome	Continuity of care	Adult patients in primary care seeing other clinicians in a variety of settings	80%
**Kollen** **2010**	1404	NL	Consumer Quality Index Continuum of Care (CQI-COC)	PCP referral and relational continuity	HCP outcome	Continuity of care	Adult patients who had been referred and visited a specialist	65%
**Uijen** **2011**	288	NL	Nijmegen Continuity Questionnaire	Hos<->PCP	HCP outcome	Continuity of care	Patients with comorbidity	72%
**Uijen** **2012**	268	NL	Nijmegen Continuity Questionnaire	Hos<->PCP	HCP outcome	Continuity of care	Patients with comorbidity	76%
			**Healthcare professional perspective**					
**Berendsen** **2010**	496	NL	Doctors’ opinions on collaboration (DOC) questionnaire	Hos<->PCP	HCP outcome and climate	Interprofessional collaboration	PCPs and specialists	45%
**Forster** **2012**	**	CAN	Peer review process of adverse outcome	Hospital discharge	Patient outcome	Adverse events at discharge	NA	NA
**Graumlich** **2008**	417	USA	Modified Physician-PREPARED	Hospital discharge	HCP outcome	Discharge	Community physicians	76%
**Hess** **2009**	12212	USA	CRP-PIM: Communication with Referring Physicians Practice Improvement Module	PCP referral to secondary care consultants (hospital and private groups)	HCP outcomeand climate	Communication of consultants	Referring physicians	?
**Nuno-solinis** **2013**	187	SP	No name reported	Hos<->PCP	Climate	Interprofessional collaboration	Physicians and nurses working in integrated healthcare organisations	16%
**Smith** **2004**	***	USA	Medication discrepancy tool	Hospital discharge	Patient outcome	Medication discrepancies	Practitioners across the continuum of care	NA

SP: Spain; NL: the Netherlands; USA: United States of America; AUS: Australia; CAN: Canada; HOS: hospital; PCP: Primary care practitioner; NA = not applicable; NR = not reported

* PCAS:79%, PCAT-S:91%, CPCI:99%, VANOCCS:64+54%

** Vignette study on 319 case report, 30 physicians

***Vignette study on 20 cases, 6 clinicians

### Quality of studies in validity assessment

The 18 included publications were published between 2001 and 2013. The quality of Forster et al. and Smith at al. could not be assessed using the COSMIN, for they did not validate a questionnaire, but reported on diagnostic tools for adverse events and medication discrepancies, respectively [42,46]. The methodological quality of the studies was overall acceptable for the COSMIN items internal consistency, content validity, structural validity, and hypothesis testing (Table 2). Reliability was only assessed by Uijen et al., and measurement error and responsiveness were not assessed at all [40]. In 14 of the 18 publications we identified methodological flaws in the validation: 1) after extensive item reduction, the resulting item set did not fully cover the original content, and objectives and content validity were not re-assessed [29,30,35,39], 2) not reporting important design choices and characteristics, such as number of items in the measurement tool, total respondents, or respondent characteristics [31,33,34,44], 3) not describing statistical analysis [44], treating a multi-dimensional construct as a unidimensional questionnaire in the analysis [32] and performing an exploratory factor analysis instead of a confirmatory analysis in one sample [32,40,41,45] and 4) ignoring strikingly low factor loadings (S1 Table) [34,44]. Graumlich et al. chose a very selective group of patients and therefore their validation is not generalizable beyond their study population [33].

**Table 2:** Quality of the studies in validity assessment; validation of measurement tools on aspects of transitional patient safety, according to the COSMIN criteria.

Author	Year	Measurement tool	Box A: Internal consistency	Box B: Reliability	Box D: Content validity	Box E: Structural validity	Box F: Hypothesis testing
**Patient perspective**							
Aller	2013	Continuity of care between care Levels (CCAENA)	excellent	–	unknown*	good	good
Berendsen	2009	Consumer Quality Index Continuum of Care (CQI-COC)	fair	–	fair	good	good
Kollen	2010		–	–	–	–	fair
Coleman	2002	Care Transition Measure (CTM)	–	–	excellent	–	poor
Coleman	2005		good	–	–	excellent	poor
Grimmer	2001	(B-)prepared	poor	–	excellent	fair	fair
Graumlich	2008		poor	–	excellent/ previously	fair	fair
Hadjistavropoulos	2008	Patient Continuity of Care Questionnaire	fair	–	fair	good	good
Haggerty	2011	Several existing questionnaires with a single dimension on management continuity: PCAS, PCAT-S, CPCI, VANOCSS	good	–	NA	good	fair
Haggerty	2012	Patient Perceived Continuity from Multiple Clinicians	good	–	excellent	good	poor**
Uijen	2011	Nijmegen Continuity Questionnaire	good	–	poor	good	good
Uijen	2012		poor	fair		good	fair
**Healthcare professional perspective**							
Berendsen	2010	Doctors’ opinions on collaboration (DOC) questionnaire	fair	–	excellent	fair	fair
Forster	2012	Peer review process of adverse outcome	NA	NA	NA	NA	NA
Graumlich	2008	Physician-PREPARED	poor		excellent	fair	fair
Hess	2009	Communication with Referring Physicians Practice Improvement Module (CRP-PIM)	poor	–	poor	poor	fair
Nuno-Solinis	2013	(unnamed)	poor	–	excellent	poor	fair
Smith	2004	Medication discrepancy tool	NA	NA	NA	NA	NA

– = not assessed in this paper; NA = not applicable

*Referred to a Spanish article on the development and first steps of validation

**Driven by of the lack of a reference standard, the researchers used an alternative reference standard that we judged not applicable as a reference standard

We excluded Box G (cross-cultural validity), Box H (criterion validity) and items D3, F7, F8 from the assessment. Box C (measurement error) and Box I (responsiveness) were not assessed in any of the included publications and therefore omitted from the table

### Results of the outcome inventory

**Fig 3:**
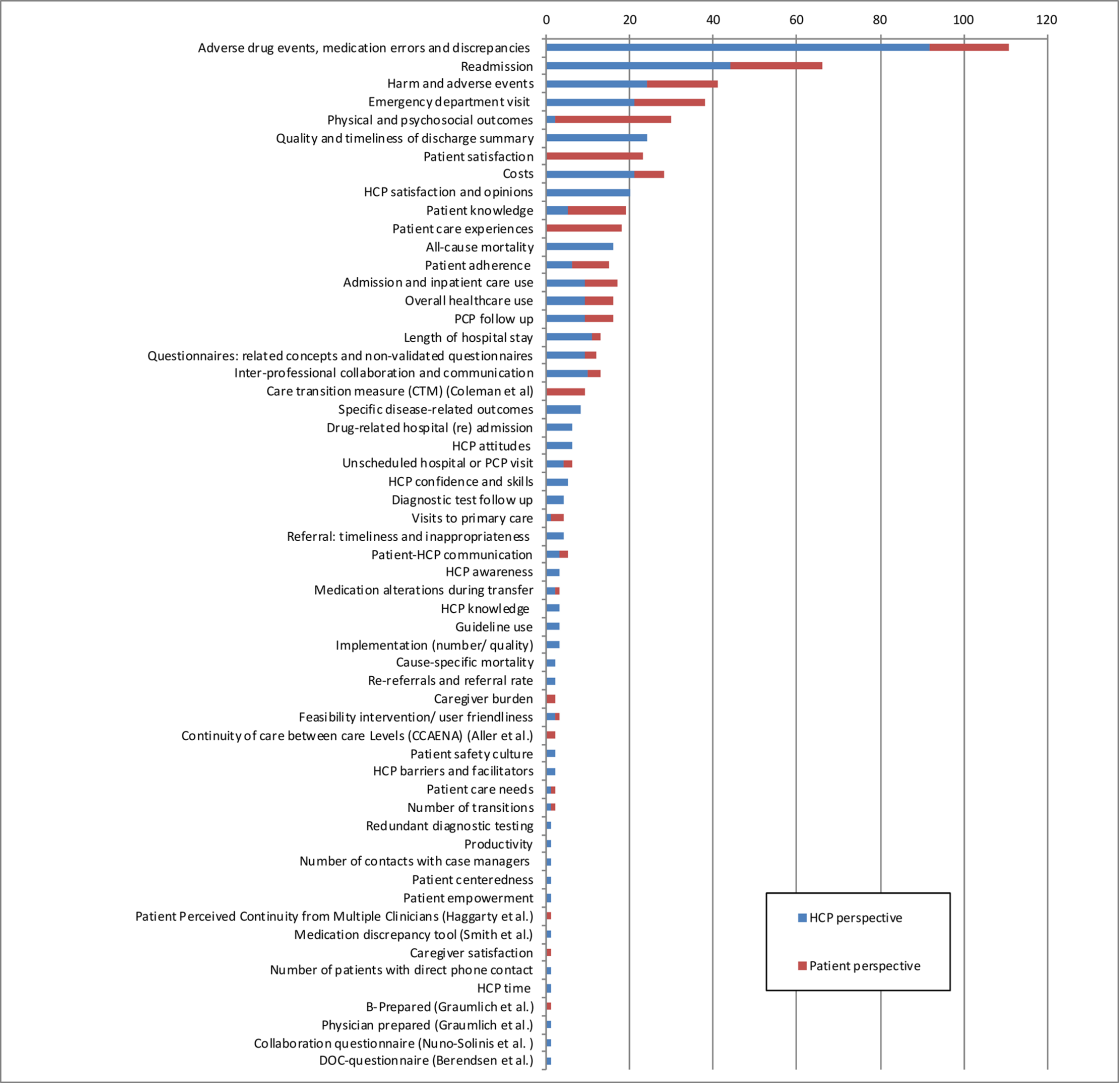
Outcomes used in transitional patient safety evaluation according to perspective. HCP = healthcare professional, PCP = Primary care practitioner.

We identified 191 publications, of which 76 reported evaluation of current transitional patient safety and 115 outcomes of interventions for improving transitional patient safety. Of the latter group, 39 were designed as a randomized controlled trial, 16 as a non-randomized controlled trial, 29 as a before-after study and 31 were had an observational design. In total, only sixteen publications (8.4%) used a validated measurement tool measuring transitional patient safety, of which 9 used the Care Transition Measure by Coleman et al. Another 15 studies (7.9%) used a validated measurement tool on a related concept or non-validated questionnaires. Furthermore, the 191 publications used 45 other outcome measures, of which the most frequently used ones were: medication discrepancies (n = 98), hospital readmissions (n = 55), adverse events (n = 34), emergency department visits (n = 33), (mental or physical) health status (n = 28), quality and timeliness of discharge summary, patient satisfaction (n = 23), and costs (n = 21). Additionally, the way these outcomes were assessed was very diverse; e.g. medication errors were measured using either medical records, trained pharmacists, or patient interviews, PCP questionnaires and incident reports and were assessed at different moments in the healthcare process. Hundred-and twenty-five publications assessed transitional patient safety only from a HCPs’ perspective, 17 only from the patients’ perspective and 49 from both perspectives (Fig 3 and S2 Table).

**Fig 4:**
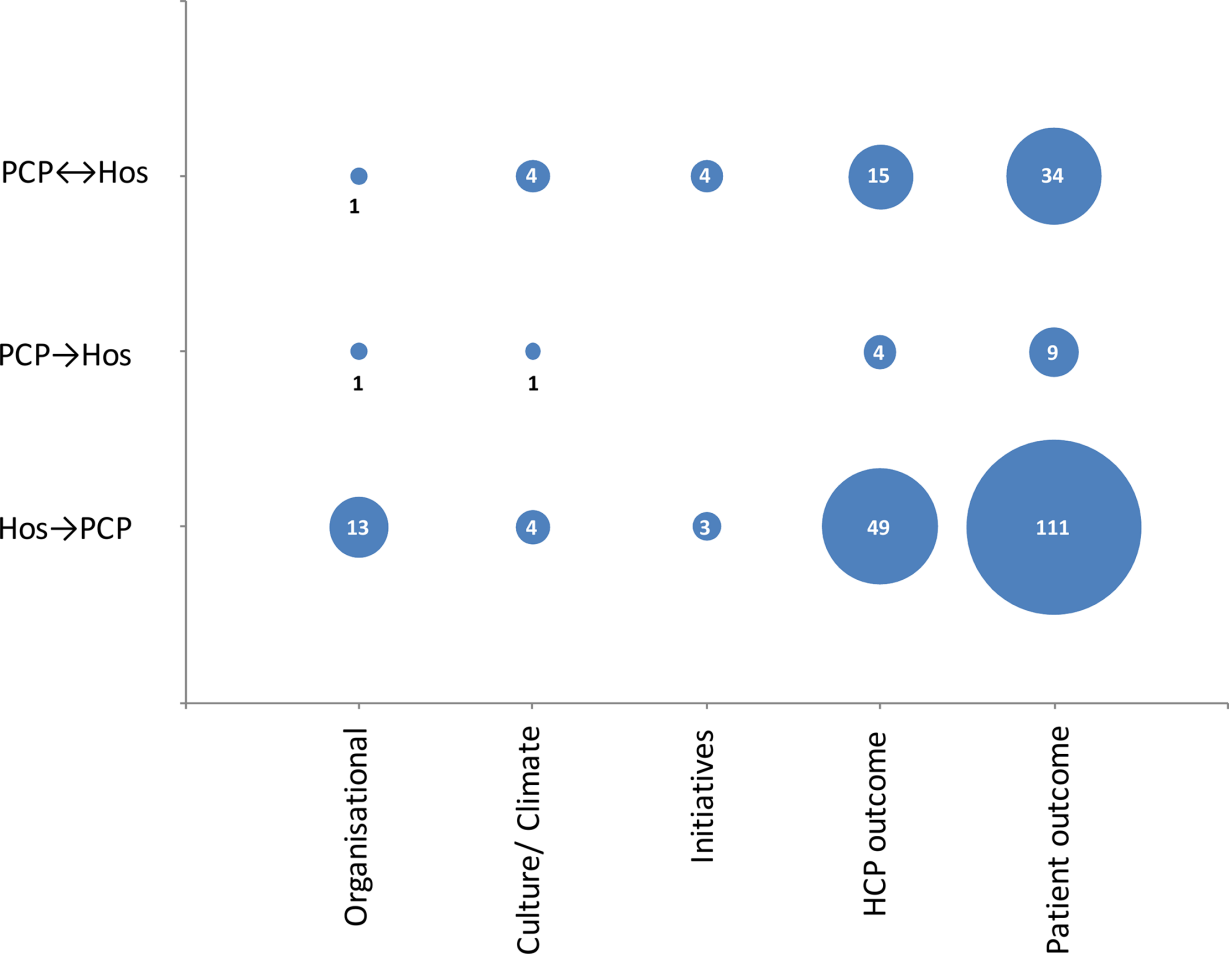
Outcome inventory: Which transitions and which safety outcome type (mentioned in Fig 1) do the selected publications include? PCP = Primary care practice, Hos = hospital, HCP = healthcare professional; number = absolute number of publications.

Of all 191 publications in this outcome inventory, 43 measured two-way patient transitions between hospital to primary care, 13 from primary care to hospital and 135 from hospital to primary care. Regarding the safety outcome type, 15 studies measured an organisational outcome, 9 measured culture, 7 initiatives, 68 a HCP outcome and 154 a patient outcome, respectively (Fig 4). A complete list of all the instruments in the outcome inventory, their sources, and characteristics is available in S3 Table.

## Discussion

Although our systematic review did not identify any measurement tool that measured the full scope of transitional patient safety, we identified 18 validation studies of 14 different instruments measuring relevant aspects of transitional patient safety. According to the COSMIN criteria, the quality of these measurement tools was generally acceptable on the items internal consistency, content validity and structural validity. Measurement tools from the patients’ perspectives performed somewhat better (quality: moderate to good) than tools from the healthcare professionals’ perspective (quality: moderate). The methodological quality of other criteria was generally poor or not addressed at all. For example, by leaving out minimal important change, test-retest reliability and responsiveness, instruments are not sufficiently validated to measure change over time or the effect of a transitional patient safety intervention.

Only few recent publications on patient safety used validated outcomes. The most often used validated instrument was the Care Transition Measure by Coleman et al., which measures only the patient preparedness for discharge [31, 32]. In total, over 45 different non-validated outcomes were used in our literature review, and most were not used consistently.

### Comparison to literature

Existing systematic reviews on measuring continuity of care (Schultz et al. and Uijen et al.), or integrated care (Strandberg-Larsen et al.) partly included the same articles we did [9, 10, 13]. However, these reviews assess coordination of care, while our review focuses on measuring transitional patient safety. Transitional patient safety encompasses the prevention of errors or reduction of adverse effects associated with transitions of care potentially causing harm to patients and is focused more on the safety outcome instead of the organisational aspects. These transitional patient safety outcomes are specifically of interest because we need to be able to measure if an intervention is actually effective on all safety outcome types: from patient safety culture to patient outcomes. In our systematic review we also disregarded instruments that assessed other quality aspects such as efficiency and effectiveness [47] and measurement tools that focused on continuity within healthcare organisations instead of transitional patient safety [48–56].

Additionally, our outcomes inventory partly includes similar articles as existing systematic reviews on interventions in transitional patient safety [22–26], especially with Hesselink’s systematic review [22]. Although the aim of this systematic review was to review interventions aiming to improve hospital discharge, they also identified the fragmented use of outcomes in literature on the effect of discharge interventions. However, our approach differs by the focus on measuring transitional patient safety outcomes, and by including all the transitions between PCP and hospital instead of looking only at discharge.

### Interpretations of findings

Although we found an acceptable methodological quality of several aspects of validity according to the COSMIN criteria, the ratings should be viewed with caution [27]. The most important step in development of a measurement tool is content validity, which scored high in the included studies. However, the majority of studies excluded items after factor analysis. In 3 of the studies Aller et al., Berendsen et al., and Uijen et al., up to half of all items were excluded, as well as entire transitions (e.g. excluding all items concerning referral, while the questionnaire was developed for overall continuity back and forth) [29,30,35,39]. This discarded the initial development and item generation process, and potentially undermined the initial content validity. This may have been induced by the common practice in current medical literature to validate measurement tools as being reflective concepts (the construct is reflected by the items, for example anxiety) [57]. Transitional patient safety, however, is a formative concept, in which the construct is the result of the presented items, for example socio-economic status [57]. Therefore, the majority studies conducted item generation in a formative way, with the intention to assemble a set of items that all together would capture the construct of transitional safety. Up to now there is no consensus among experts on the optimal way to assess validity of formative measurement tools, and therefore construction of domains is usually performed ‘as usual’, i.e. in a reflective way using exploratory factor analysis [58, 59]. As a result of this approach, many items relevant for transitional safety were excluded from final versions of measurement tools.

Many instruments in our review could not be adequately validated because of the absence of a valid comparator or reference standard. This aspect of validation is often ignored, and solved by using an internal comparator question, single outcomes such as the number of emergency department visits, or other proxies that only poorly relate to transitional patient safety. This was reflected in poor quality scores in the boxes “hypothesis testing” and “criterion validity”, thus lowering the overall rating. In contrast, other important aspects of validity such as reliability and measurement error which could have been assessed were not performed.

The outcome inventory showed that the large majority of studies only measured transitional patient safety from one perspective, presenting a limited view. Most studies approached transitional patient safety from the HCP perspective which covers only part of the transition. Fifteen studies measured transitional patient safety from the patients’ perspective, underlining the importance of the patients’ perspective as being the only stakeholder that fully experiences the healthcare transitions [6]. However, 42 of the 191 studies combined the patients’ and HCPs’ perspectives, leading to a more complete view.

The outcome inventory also demonstrated that the majority of studies focused on single directional transitions, specifically on discharge. This leaves referral from primary care to hospital underrepresented. On the safety outcome type, the majority of studies measure HCP and patient outcomes, leaving especially culture and climate unmeasured. These gaps provide insight into the niches to fill by future research on interventions and measurement tools needed for transitional patient safety.

### Strengths and limitations

We combined a very broad search strategy on transitional care with validated search filters on patient safety and measurement tools. This resulted in a broad range of papers and a wide selection of validated measurement tools. The broad search strategy also provided a wide range of publications measuring either the current state of transitional patient safety or improvement of transitional patient safety in intervention studies. We included all types of research design as often patient safety is assessed in observational studies [60]. Including only randomised controlled trials would have limited the overview and subsequently the generalizability of the results.

A limitation may have been that the concept of transitional patient safety that we used is relatively new, moving away from the traditional single organisation safety concept to a more patient-centered view across multiple healthcare settings. Therefore, the specific term “transitional patient safety” is not yet commonly used in literature. In addition, of all articles included in the full text selection, 75 (11.9%) could not be found. This might have led to the omission of relevant publications in our article selection for both the validity assessment and the outcome inventory. However, as the large majority of these studies concerned the inclusion into the outcome inventory, we believe the completeness of identified validated measurement tools was not affected.

Lastly, the COSMIN criteria are originally designed for patient reported outcome measurements, which did not match all the instruments we included [27]. The COSMIN criteria, however, are frequently used in other types of measurement tools, such as HCP-reported measurement tools. However, the absence of a reference standard in transitional patient safety limits the applicability of COSMIN criteria (e.g. criterion validity, hypothesis testing). And importantly, as we intended to identify all validated measurement tools developed for transitional patient safety, we also identified two studies validating review methods for transitional safety incidents (Smith et al. and Forster et al.) [42, 46]. Even though quality could not be assessed using the COSMIN criteria, these measurement tools are useful, as they could help other healthcare organisations identify and improve their identified weaknesses.

### Implications

With this systematic review, we provide an overview of the available validated measurement tools on transitional patient safety. Although their quality was generally acceptable, the validation process could be improved considerably, specifically for their use in intervention studies. For this, we need better methodological methods, e.g. for validation of measurement tools on formative constructs, handling validation when a reference standard is not available, and quality assessment of such measurement tools. Furthermore, the validated measurement tools are scarcely used in current effect studies while a broad array of other outcomes measures is used. Besides cautiousness with interpretation of current studies because of these methodological flaws, we need to develop a standardised set of well validated measurement tools in order to be able to compare current transitional patient safety between settings and the effect of interventions. Lastly, none of the tools measures all aspects of transitional patient safety outcomes; especially measurement tools for transitional patient safety culture and the referral process are needed.

## Conclusion

The concept of transitional patient safety is evolving, resulting in the use of a wide variety of outcome measures and measurement tools. Adequate monitoring of transitional patient safety in future requires standardised measurements, to be used in adjacent healthcare settings that care for the same patients in order to identify best practices and to assess the effectiveness of care transition interventions. This systematic review could be used as a base for developing this set of measurement tools.

## Supporting information

S1 Prisma Checklist (DOC)

S1 Text Search strategy. (DOCX)

S2 Text In- and exclusion criteria. (DOCX)

S1 Table Validity assessment: Characteristics included studies. SP: Spain; NL: the Netherlands; USA: United States of America; AUS: Australia; CAN: Canada; HOS: hospital; PCP: Primary care provider, NA = not applicable, NR = not reported, GP = general practitioner.
* PCAS:79%, PCAT-S:91%, CPCI:99%, VANOCCS:64+54%.
¥ PCAS:342, PCAT-S:392, CPCI:427, VANOCCS:278+136.
§ Vignette study on 319 case reports, 30 physicians.
ǂ Vignette study on 20 cases, 6 clinicians.
# Primary Care Assessment Survey (PCAS), the Primary Care Assessment Tool–Short Form (PCAT-S), the Components of Primary Care.
Instrument (CPCI) and the Veterans Affairs National Outpatient Customer Satisfaction Survey (VANOCSS).
(DOCX)

S2 Table Outcome inventory: Used outcomes from healthcare professionals’ and patients’ perspectives inventoried in 191 publications that measured transitional patient safety: Which are used and how often. As some publications measured more than one outcome or one outcome from both the perspective of the healthcare professional and the patient, the added numbers can exceed the total.
HCP = healthcare professional, PCP = Primary care practitioner. Many studies used more than one outcome, and from different perspectives. Therefore, the sub-totals can add up to more than the total outcomes. *Brain natriuretic peptide (BNP), Hemoglobine A1c, lipid levels, volume status, renal function, weight, International Normalized Ratio (INR), haemorrhagic events, thromboembolic events, post referral colonoscopy delay.
(DOCX)

S3 Table Outcome inventory: Complete data extraction and references. ADE = Adverse drug event; AE = Adverse event; DOC-questionnaire = Doctors’ Opinions on Collaboration-questionnaire; ED = Emergency department; GP = General practitioner; HCP = Healthcare professional; PCP = Primary care physician; RCT = Randomised controlled trial.
(DOCX)
