# Supplementary material for: Measurement tools and outcome measures used in transitional patient safety; a systematic review
Source: PLoS One. 2018 Jun 4;13(6):e0197312. doi: 10.1371/journal.pone.0197312 (PMC5986135; doi:10.1371/journal.pone.0197312)
Supplement: S1 Text — (DOCX) [file pone.0197312.s002.docx]

**S1 Text. SEARCH STRATEGY**

**PUBMED (INCLUDING MEDLINE)**

**1. Transitional care**((Continuity[tiab] OR Continuum[tiab] OR Integration[tiab] OR Integrated[tiab] OR Integrating[tiab] OR Integral[tiab] OR “Patient-centered”[tiab] OR Transitional[tiab] OR Transition[tiab] OR Transmural[tiab] OR Seamless[tiab] OR Crossboundary[tiab] OR “Cross-boundary”[tiab]) AND (care[tiab] OR healthcare[tiab])) OR (Continuity[tiab] AND service[tiab]) OR “continuity of patient care”[Mesh]

**2. Measurement (Terwee et al.)**

(instrumentation[sh] OR methods[sh] OR Validation Studies[pt] OR Comparative Study[pt] OR psychometrics[MeSH] OR psychometr*[tiab] OR clinimetr*[tw] OR clinometr*[tw] OR “outcome assessment (health care)”[MeSH] OR outcome assessment[tiab] OR outcome measure*[tw] OR “observer variation”[MeSH] OR observer variation[tiab] OR “Health Status Indicators”[Mesh] OR “reproducibility of results”[MeSH] OR reproducib*[tiab] OR “discriminant analysis”[MeSH] OR reliab*[tiab] OR unreliab*[tiab] OR valid*[tiab] OR coefficient[tiab] OR homogeneity[tiab] OR homogeneous[tiab] OR “internal consistency”[tiab] OR (cronbach*[tiab] AND (alpha[tiab] OR alphas[tiab])) OR (item[tiab] AND (correlation*[tiab] OR selection*[tiab] OR reduction*[tiab])) OR agreement[tiab] OR precision[tiab] OR imprecision[tiab] OR “precise values”[tiab] OR test–retest[tiab] OR (test[tiab] AND retest[tiab]) OR (reliab*[tiab] AND (test[tiab] OR retest[tiab])) OR stability[tiab] OR interrater[tiab] OR inter-rater[tiab] OR intrarater[tiab] OR intra-rater[tiab] OR intertester[tiab] OR inter-tester[tiab] OR intratester[tiab] OR intra-tester[tiab] OR interobserver[tiab] OR inter-observer[tiab] OR intraobserver[tiab] OR intra-observer[tiab] OR intertechnician[tiab] OR inter-technician[tiab] OR intratechnician[tiab] OR intra-technician[tiab] OR interexaminer[tiab] OR inter-examiner[tiab] OR intraexaminer[tiab] OR intra-examiner[tiab] OR interassay[tiab] OR inter-assay[tiab] OR intraassay[tiab] OR intra-assay[tiab] OR interindividual[tiab] OR inter-individual[tiab] OR intraindividual[tiab] OR intra-individual[tiab] OR interparticipant[tiab] OR inter-participant[tiab] OR intraparticipant[tiab] OR intra-participant[tiab] OR kappa[tiab] OR kappa’s[tiab] OR kappas[tiab] OR repeatab*[tiab] OR ((replicab*[tiab] OR repeated[tiab]) AND (measure[tiab] OR measures[tiab] OR findings[tiab] OR result[tiab] OR results[tiab] OR test[tiab] OR tests[tiab])) OR generaliza*[tiab] OR generalisa*[tiab] OR concordance[tiab] OR (intraclass[tiab] AND correlation*[tiab]) OR discriminative[tiab] OR “known group”[tiab] OR factor analysis[tiab] OR factor analyses[tiab] OR dimension*[tiab] OR subscale*[tiab] OR (multitrait[tiab] AND scaling[tiab] AND (analysis[tiab] OR analyses[tiab])) OR item discriminant[tiab] OR interscale correlation*[tiab] OR error[tiab] OR errors[tiab] OR “individual variability”[tiab] OR (variability[tiab] AND (analysis[tiab] OR values[tiab])) OR (uncertainty[tiab] AND (measurement[tiab] OR measuring[tiab])) OR “standard error of measurement”[tiab] OR sensitiv*[tiab] OR responsive*[tiab] OR ((minimal[tiab] OR minimally[tiab] OR clinical[tiab] OR clinically[tiab]) AND (important[tiab] OR significant[tiab] OR detectable[tiab]) AND (change[tiab] OR difference[tiab])) OR (small*[tiab] AND (real[tiab] OR detectable[tiab]) AND (change[tiab] OR difference[tiab])) OR meaningful change[tiab] OR “ceiling effect”[tiab] OR “floor effect”[tiab] OR “Item response model”[tiab] OR IRT[tiab] OR Rasch[tiab] OR “Differential item functioning”[tiab] OR DIF[tiab] OR “computer adaptive testing”[tiab] OR “item bank”[tiab] OR “cross-cultural equivalence”[tiab])

**3. Patient safety (Tanon et al.)**

(Safe*[tiab] OR Safety[Mesh] OR Err*[tiab] OR Adverse[tiab]) AND (“Risk Management”[Mesh] OR “Quality of Health Care”[Mesh] OR “Medical Errors”[Mesh] OR “Safety Management”[Mesh] OR “Medical Audit”[Mesh]) OR “patient safety”[Mesh]

**EMBASE**

**1. Transitional care**((‘Continuity’:ab,ti OR ‘Continuum’:ab,ti OR ‘Integration’:ab,ti OR ‘Integrated’:ab,ti OR ‘Integrating’:ab,ti OR ‘Integral’:ab,ti OR ‘Patient-centered’:ab,ti OR ‘Transitional’:ab,ti OR ‘Transition’:ab,ti OR ‘Transmural ‘:ab,ti OR ‘Seamless’:ab,ti OR ‘Crossboundary’:ab,ti OR ‘Cross-boundary’:ab,ti) AND (‘care’:ab,ti OR ‘healthcare’:ab,ti)) OR (‘Continuity’:ab,ti AND ‘service’:ab,ti) OR ‘integrated health care system’/exp AND [embase]/lim

**2. Measurement (Terwee et al.)**

('intermethod comparison'/exp OR 'data collection method'/exp OR 'validation study'/exp OR 'feasibility study'/exp OR 'pilot study'/exp OR 'psychometry'/exp OR 'reproducibility'/exp OR reproducib*:ab,ti OR 'audit':ab,ti OR psychometr*:ab,ti OR clinimetr*:ab,ti OR clinometr*:ab,ti OR 'observer variation'/exp OR 'observer variation':ab,ti OR 'discriminant analysis'/exp OR 'validity'/exp OR reliab*:ab,ti OR valid*:ab,ti OR 'coefficient':ab,ti OR 'internal consistency':ab,ti OR (cronbach*:ab,ti AND ('alpha':ab,ti OR 'alphas':ab,ti)) OR 'item correlation':ab,ti OR 'item correlations':ab,ti OR 'item selection':ab,ti OR 'item selections':ab,ti OR 'item reduction':ab,ti OR 'item reductions':ab,ti OR 'agreement':ab,ti OR 'precision':ab,ti OR 'imprecision':ab,ti OR 'precise values':ab,ti OR 'test-retest':ab,ti OR ('test':ab,ti AND 'retest':ab,ti) OR (reliab*:ab,ti AND ('test':ab,ti OR 'retest':ab,ti)) OR 'stability':ab,ti OR 'interrater':ab,ti OR 'inter-rater':ab,ti OR 'intrarater':ab,ti OR 'intra-rater':ab,ti OR 'intertester':ab,ti OR 'inter-tester':ab,ti OR 'intratester':ab,ti OR 'intra- tester':ab,ti OR 'interobeserver':ab,ti OR 'inter-observer':ab,ti OR 'intraobserver':ab,ti OR 'intra- observer':ab,ti OR 'intertechnician':ab,ti OR 'inter-technician':ab,ti OR 'intratechnician':ab,ti OR 'intra- technician':ab,ti OR 'interexaminer':ab,ti OR 'inter-examiner':ab,ti OR 'intraexaminer':ab,ti OR 'intra- examiner':ab,ti OR 'interassay':ab,ti OR 'inter-assay':ab,ti OR 'intraassay':ab,ti OR 'intra-assay':ab,ti OR 'interindividual':ab,ti OR 'inter-individual':ab,ti OR 'intraindividual':ab,ti OR 'intra-individual':ab,ti OR 'interparticipant':ab,ti OR 'inter-participant':ab,ti OR 'intraparticipant':ab,ti OR 'intra- participant':ab,ti OR 'kappa':ab,ti OR 'kappas':ab,ti OR 'coefficient of variation':ab,ti OR repeatab*:ab,ti OR (replicab*:ab,ti OR 'repeated':ab,ti AND ('measure':ab,ti OR 'measures':ab,ti OR 'findings':ab,ti OR 'result':ab,ti OR 'results':ab,ti OR 'test':ab,ti OR 'tests':ab,ti)) OR generaliza*:ab,ti OR generalisa*:ab,ti OR 'concordance':ab,ti OR ('intraclass':ab,ti AND correlation*:ab,ti) OR 'discriminative':ab,ti OR 'known group':ab,ti OR 'factor analysis':ab,ti OR 'factor analyses':ab,ti OR 'factor structure':ab,ti OR 'factor structures':ab,ti OR 'dimensionality':ab,ti OR subscale*:ab,ti OR 'multitrait scaling analysis':ab,ti OR 'multitrait scaling analyses':ab,ti OR 'item discriminant':ab,ti OR 'interscale correlation':ab,ti OR 'interscale correlations':ab,ti OR ('error':ab,ti OR 'errors':ab,ti AND (measure*:ab,ti OR correlat*:ab,ti OR evaluat*:ab,ti OR 'accuracy':ab,ti OR 'accurate':ab,ti OR 'precision':ab,ti OR 'mean':ab,ti)) OR 'individual variability':ab,ti OR 'interval variability':ab,ti OR 'rate variability':ab,ti OR 'variability analysis':ab,ti OR ('uncertainty':ab,ti AND ('measurement':ab,ti OR 'measuring':ab,ti)) OR 'standard error of measurement':ab,ti OR sensitiv*:ab,ti OR responsive*:ab,ti OR ('limit':ab,ti AND 'detection':ab,ti) OR 'minimal detectable concentration':ab,ti OR interpretab*:ab,ti OR (small*:ab,ti AND ('real':ab,ti OR 'detectable':ab,ti) AND ('change':ab,ti OR 'difference':ab,ti)) OR 'meaningful change':ab,ti OR 'minimal important change':ab,ti OR 'minimal important difference':ab,ti OR 'minimally important change':ab,ti OR 'minimally important difference':ab,ti OR 'minimal detectable change':ab,ti OR 'minimal detectable difference':ab,ti OR 'minimally detectable change':ab,ti OR 'minimally detectable difference':ab,ti OR 'minimal real change':ab,ti OR 'minimal real difference':ab,ti OR 'minimally real change':ab,ti OR 'minimally real difference':ab,ti OR 'ceiling effect':ab,ti OR 'floor effect':ab,ti OR 'item response model':ab,ti OR 'irt':ab,ti OR 'rasch':ab,ti OR 'differential item functioning':ab,ti OR 'dif':ab,ti OR 'computer adaptive testing':ab,ti OR 'item bank':ab,ti OR 'cross-cultural equivalence':ab,ti OR ‘outcome assessment’/exp OR ‘medical record’:ab,ti) AND [embase]/lim

**3. Patient safety (Tanon et al.)**

((Safety/exp OR Safe*:ti,ab OR Err*:ti,ab OR Adverse:ti,ab) AND (‘Health Care Quality’/exp OR ‘Risk assessment’/exp) OR ‘patient safety'/exp) AND [embase]/lim

**CINAHL (EBSCO)**

**1. Transitional care**

TI (( “Continuity” OR “Continuum” OR “Integration” OR “Integrated” OR “Integrating” OR “Integral” OR “Patient-centered” OR “Transitional” OR “Transition” OR “Transmural ” OR “Seamless” OR “Crossboundary” OR “Cross-boundary” AND ("care" OR "healthcare")) OR AB (( “Continuity” OR “Continuum” OR “Integration” OR “Integrated” OR “Integrating” OR “Integral” OR “Patient-centered” OR “Transitional” OR “Transition” OR “Transmural ” OR “Seamless” OR “Crossboundary” OR “Cross-boundary” AND ("care" OR "healthcare")) OR TI (continuity of patient care OR continuity of care) OR AB (continuity of patient care OR continuity of care) OR (MH "Continuity of Patient Care+")

**2. Measurement (Terwee et al.)**

TI psychometr* OR TI observer variation OR TI reproducib* OR TI reliab* OR TI unreliab* OR TI valid* OR TI coefficient OR TI homogeneity OR TI homogeneous OR TI “internal consistency” OR AB psychometr* OR AB observer variation OR AB reproducib* OR AB reliab* OR AB unreliab* OR AB valid* OR AB coefficient OR AB homogeneity OR AB homogeneous OR AB “internal consistency” OR (TI cronbach* OR AB cronbach* AND (TI alpha OR AB alpha OR TI alphas OR AB alphas)) OR (TI item OR AB item AND (TI correlation* OR AB correlation* OR TI selection* OR AB selection* OR TI reduction* OR AB reduction*)) OR TI agreement OR TI precision OR TI imprecision OR TI “precise values” OR TI test-retest OR AB agreement OR AB precision OR AB imprecision OR AB “precise values” OR AB test-retest OR (TI test OR AB test AND (TI retest OR AB retest)) OR (TI reliab* OR AB reliab* AND (TI test OR AB test OR TI retest or AB retest)) OR TI stability OR TI interrater OR TI interrater OR TI intrarater OR TI intra-rater OR TI intertester OR TI inter-tester OR TI intratester OR TI intra-tester OR TI interobserver OR TI inter-observer OR TI intraobserver OR TI intra-observer OR TI intertechnician OR TI inter-technician OR TI intratechnician OR TI intra-technician OR TI interexaminer OR TI inter-examiner OR TI intraexaminer OR TI intra-examiner OR TI interassay OR TI inter-assay OR TI intraassay OR TI intra-assay OR TI interindividual OR TI inter-individual OR TI intraindividual OR TI intra-individual OR TI interparticipant OR TI inter-participant OR TI intraparticipant OR TI intra-participant OR TI kappa OR TI kappa’s OR TI kappas OR TI repeatab* OR AB stability OR AB interrater OR AB inter-rater OR AB intrarater OR AB intra-rater OR AB intertester OR AB inter-tester OR AB intratester OR AB intra-tester OR AB interobserver OR AB inter-observer OR AB intraobserver OR AB intra-observer OR AB intertechnician OR AB inter-technician OR AB intratechnician OR AB intra-technician OR AB interexaminer OR AB inter-examiner OR AB intraexaminer OR AB intra-examiner OR AB interassay OR AB inter-assay OR AB intraassay OR AB intra-assay OR AB interindividual OR AB inter-individual OR AB intraindividual OR AB intra-individual OR AB interparticipant OR AB inter-participant OR AB intraparticipant OR AB intra-participant OR AB kappa OR AB kappa’s OR AB kappas OR AB repeatab* OR ((TI replicab* OR AB replicab* OR TI repeated OR AB repeated) AND (TI measure OR AB measure OR TI measures OR AB measures OR TI findings OR AB findings OR TI result OR AB result OR TI results OR AB results OR TI test OR AB test OR TI tests OR AB tests)) OR TI generaliza* OR TI generalisa* OR TI concordance OR AB generaliza* OR AB generalisa* OR AB concordance OR (TI intraclass OR AB intraclass AND (TI correlation* or AB correlation*)) OR TI discriminative OR TI “known group” OR TI factor analysis OR TI factor analyses OR TI dimension* OR TI subscale* OR AB discriminative OR AB “known group” OR AB factor analysis OR AB factor analyses OR AB dimension* OR AB subscale* OR (TI multitrait OR AB multitrait AND (TI scaling OR AB scaling) AND (TI analysis OR AB analysis OR TI analyses OR AB analyses)) OR TI item discriminant OR TI interscale correlation* OR TI error OR TI errors OR TI “individual variability” OR AB item discriminant OR AB interscale correlation* OR AB error OR AB errors OR AB “individual variability” OR (TI variability OR AB variability AND (TI analysis OR AB analysis OR TI values OR AB values)) OR (TI uncertainty OR AB uncertainty AND (TI measurement OR AB measurement OR TI measuring OR AB measuring)) OR TI “standard error of measurement” OR TI sensitiv* OR TI responsive* OR AB “standard error of measurement” OR AB sensitiv* OR AB responsive* OR ((TI minimal OR TI minimally OR TI clinical OR TI clinically OR AB minimal OR AB minimally OR AB clinical OR AB clinically) AND (TI important OR TI significant OR TI detectable OR AB important OR AB significant OR AB detectable) AND (TI change OR AB change OR TI difference OR AB difference)) OR (TI small* OR AB small* AND (TI real OR AB real OR TI detectable OR AB detectable) AND (TI change OR AB change OR TI difference OR AB difference)) OR TI meaningful change OR TI “ceiling effect” OR TI “floor effect” OR TI “Item response model” OR TI IRT OR TI Rasch OR TI “Differential item functioning” OR TI DIF OR TI “computer adaptive testing” OR TI “item bank” OR TI “cross-cultural equivalence” OR TI outcome assessment OR AB meaningful change OR AB “ceiling effect” OR AB “floor effect” OR AB “Item response model” OR AB IRT OR AB Rasch OR AB “Differential item functioning” OR AB DIF OR AB “computer adaptive testing” OR AB “item bank” OR AB cross-cultural equivalence” OR AB outcome assessment

**3. Patient safety (Tanon et al.)**

((TX Health* OR TX Care* OR TX Prevent*) AND (TX Safe* OR TX Err*) AND (MH “Child” OR TX Patient* or TX Human)) OR MH “Health Care Errors+”.

**PSYCHINFO (OVID)**

1. **Transitional care**

(( Continuity.ti OR Continuum.ti OR Integration.ti OR Integrated.ti OR Integrating.ti OR Integral.ti OR Patient-centered.ti OR Transitional.ti OR Transition.ti OR Transmural.ti OR Seamless.ti OR Crossboundary.ti OR Cross-boundary.ti) AND (care.ti OR healthcare.ti OR care.ab OR healthcare.ab)) OR (( Continuity.ab OR Continuum.ab OR Integration.ab OR Integrated.ab OR Integrating.ab OR Integral.ab OR Patient-centered.ab OR Transitional.ab OR Transition.ab OR Transmural.ab OR Seamless.ab OR Crossboundary.ab OR Cross-boundary.ab) AND (care.ti OR healthcare.ti OR care.ab OR healthcare.ab))

**2. Measurement (Terwee et al.)**

psychometr*.ti OR observer variation.ti OR reproducib*.ti OR reliab*.ti OR unreliab*.ti OR valid*.ti OR coefficient.ti OR homogeneity.ti OR homogeneous.ti OR internal consistency.ti OR psychometr*.ab OR observer variation.ab OR reproducib*.ab OR reliab*.ab OR unreliab*.ab OR valid*.ab OR coefficient.ab OR homogeneity.ab OR homogeneous.ab OR internal consistency.ab OR (cronbach*.ti OR cronbach*.ab AND (alpha.ti OR alpha.ab OR alphas.ti OR alphas.ab)) OR ((item.ti OR item.ab) AND (correlation*.ti OR correlation*.ab OR selection*.ti OR selection*.ab OR reduction*.ti OR reduction*.ab)) OR agreement.ti OR precision.ti OR imprecision.ti OR precise values.ti OR test-retest.ti OR agreement.ab OR precision.ab OR imprecision.ab OR precise values.ab OR test-retest.ab OR (test.ti OR test.ab AND retest.ti OR retest.ab) OR (reliab*.ti OR reliab*.ab AND (test.ti OR test.ab OR retest.ti or retest.ab)) OR stability.ti OR interrater.ti OR interrater.ti OR intrarater.ti OR intra-rater.ti OR intertester.ti OR inter-tester.ti OR intratester.ti OR intra-tester.ti OR interobserver.ti OR inter-observer.ti OR intraobserver.ti OR intra-observer.ti OR intertechnician.ti OR inter-technician.ti OR intratechnician.ti OR intra-technician.ti OR interexaminer.ti OR inter-examiner.ti OR intraexaminer.ti OR intra-examiner.ti OR interassay.ti OR inter-assay.ti OR intraassay.ti OR intra-assay.ti OR interindividual.ti OR inter-individual.ti OR intraindividual.ti OR intra-individual.ti OR interparticipant.ti OR inter-participant.ti OR intraparticipant.ti OR intra-participant.ti OR kappa.ti OR kappa-s.ti OR kappas.ti OR repeatab*.ti OR stability.ab OR interrater.ab OR inter-rater.ab OR intrarater.ab OR intra-rater.ab OR intertester.ab OR inter-tester.ab OR intratester.ab OR intra-tester.ab OR interobserver.ab OR inter-observer.ab OR intraobserver.ab OR intra-observer.ab OR intertechnician.ab OR inter-technician.ab OR intratechnician.ab OR intra-technician.ab OR interexaminer.ab OR inter-examiner.ab OR intraexaminer.ab OR intra-examiner.ab OR interassay.ab OR inter-assay.ab OR intraassay.ab OR intra-assay.ab OR interindividual.ab OR inter-individual.ab OR intraindividual.ab OR intra-individual.ab OR interparticipant.ab OR inter-participant.ab OR intraparticipant.ab OR intra-participant.ab OR kappa.ab OR kappa-s.ab OR kappas.ab OR repeatab*.ab OR ((replicab*.ti OR replicab*.ab OR repeated.ti OR repeated.ab) AND (measure.ti OR measure.ab OR measures.ti OR measures.ab OR findings.ti OR findings.ab OR result.ti OR result.ab OR results.ti OR results.ab OR test.ti OR test.ab OR tests.ti OR tests.ab)) OR generaliza*.ti OR generalisa*.ti OR concordance.ti OR generaliza*.ab OR generalisa*.ab OR concordance.ab OR (intraclass.ti OR intraclass.ab AND correlation*.ti or correlation*.ab) OR discriminative.ti OR known group.ti OR factor analysis.ti OR factor analyses.ti OR dimension*.ti OR subscale*.ti OR discriminative.ab OR known group.ab OR factor analysis.ab OR factor analyses.ab OR dimension*.ab OR subscale*.ab OR (multitrait.ti OR multitrait.ab AND scaling.ti OR scaling.ab AND (analysis.ti OR analysis.ab OR analyses.ti OR analyses.ab)) OR item discriminant.ti OR interscale correlation*.ti OR error.ti OR errors.ti OR individual variability.ti OR item discriminant.ab OR interscale correlation*.ab OR error.ab OR errors.ab OR individual variability.ab OR (variability.ti OR variability.ab AND (analysis.ti OR analysis.ab OR values.ti OR values.ab)) OR (uncertainty.ti OR uncertainty.ab AND (measurement.ti OR measurement.ab OR measuring.ti OR measuring.ab)) OR standard error of measurement.ti OR sensitiv*.ti OR responsive*.ti OR standard error of measurement.ab OR sensitiv*.ab OR responsive*.ab OR ((minimal.ti OR minimally.ti OR clinical.ti OR clinically.ti OR minimal.ab OR minimally.ab OR clinical.ab OR clinically.ab) AND (important.ti OR significant.ti OR detectable.ti OR important.ab OR significant.ab OR detectable.ab) AND (change.ti OR change.ab OR difference.ti OR difference.ab)) OR (small*.ti OR small*.ab AND (real.ti OR real.ab OR detectable.ti OR detectable.ab) AND (change.ti OR change.ab OR difference.ti OR difference.ab)) OR meaningful change.ti OR ceiling effect.ti OR floor effect.ti OR Item response model.ti OR IRT.ti OR Rasch.ti OR Differential item functioning.ti OR DIF.ti OR computer adaptive testing.ti OR item bank.ti OR cross-cultural equivalence.ti OR outcome assessment.ti OR meaningful change.ab OR ceiling effect.ab OR floor effect.ab OR Item response model.ab OR IRT.ab OR Rasch.ab OR Differential item functioning.ab OR DIF.ab OR computer adaptive testing.ab OR item bank.ab OR cross-cultural equivalence.ab OR outcome assessment.ab OR psychometrics/ OR exp Measurement/ OR exp Interrater Reliability/ exp OR Test Reliability/ OR exp Test Validity/

**3. Patient safety (Tanon et al.)**

((Safe*.ti OR Err*.ti OR Adverse.ti) OR (Safe*.ab OR Err*.ab OR Adverse.ab) OR exp Safety/) AND (risk management/ OR exp "Quality of Care"/ OR exp Errors/ OR exp Accident Prevention/ OR exp Clinical Audits/) OR patient safety.ti OR patient safety.ab
