# Supplementary material for: Measurement tools and outcome measures used in transitional patient safety; a systematic review
Source: PLoS One. 2018 Jun 4;13(6):e0197312. doi: 10.1371/journal.pone.0197312 (PMC5986135; doi:10.1371/journal.pone.0197312)
Supplement: S2 Text — (DOCX) [file pone.0197312.s003.docx]

**S2 Text. In- and exclusion criteria**

**Inclusion criteria:**

- Quantitative measurements
- Patients of all ages
- Physical and mental health
- No limits on study design
- Research assessing transitional patient safety:
  Group 1: Validity assessment: Research regarding measurement properties (development, validation or evaluation) of measurement tools in transitional patient safety

Group 2: Outcome inventory: Research regarding the current state of, or interventions on transitional patient safety and measuring their effect

- Studies on transitional patient safety between general practitioner/ primary care practitioner/ family medicine and hospital. A transition of patient responsibility from a healthcare professional (general practice/primary care or hospital) to another healthcare professional in the other level (general practice/primary care or hospital) is essential.
- Studies on patient safety culture/ climate (definition: the norm, values and assumptions of healthcare professionals on transitional patient safety or “How do healthcare professionals think about transitional patient safety”)
- Studies on initiatives and staff outcomes: behaviour of healthcare professionals (“How do healthcare professionals behave in transitional care”, e.g. referral, discharge, simultaneous care, medication reconciliation, quality indicators, discharge/ referral letters or other communication)
- Studies on patient outcomes: transitional patient safety incidents and mortality/ morbidity outcomes and patient-reported outcome measures (PROMs): patients perspectives (“How does the patient perceive transitional patient safety”)
- Measurements from both the perspectives of the healthcare professional (process and culture) and patient
- Studies on a medical home (which includes a primary care team)

**Exclusion criteria:**

- Qualitative measurements
- Measurement tools addressing more organisational concepts such as integral care
- Research regarding transitions between other healthcare organisations in primary and secondary care (e.g. ambulance, obstetric health center, nursing homes)
- Models and frameworks
- Electronic health record implementation
- Relational continuity of care
- Home healthcare (only included if a primary care provider is clearly involved)
