# Supplementary material for: Measurement tools and outcome measures used in transitional patient safety; a systematic review
Source: PLoS One. 2018 Jun 4;13(6):e0197312. doi: 10.1371/journal.pone.0197312 (PMC5986135; doi:10.1371/journal.pone.0197312)
Supplement: S1 Table — SP: Spain; NL: the Netherlands; USA: United States of America; AUS: Australia; CAN: Canada; HOS: hospital; PCP: Primary care provider, NA = not applicable, NR = not reported, GP = general practitioner. * PCAS:79%, PCAT-S:91%, CPCI:99%, VANOCCS:64+54%. ¥ PCAS:342, PCAT-S:392, CPCI:427, VANOCCS:278+136. § Vignette study on 319 case reports, 30 physicians. ǂ Vignette study on 20 cases, 6 clinicians. # Primary Care Assessment Survey (PCAS), the Primary Care Assessment Tool–Short Form (PCAT-S), the Components of Primary Care. Instrument (CPCI) and the Veterans Affairs National Outpatient Customer Satisfaction Survey (VANOCSS). (DOCX) [file pone.0197312.s004.docx]

| **S3 Table. Validity assessment: Characteristics of the included studies** | | | | | | | | | | | | | | | | | | | | |
| --- | --- | --- | --- | --- | --- | --- | --- | --- | --- | --- | --- | --- | --- | --- | --- | --- | --- | --- | --- | --- |
| ***Author/Year***  Country  Response rate  Mean age  Gender | **Measurement tool** | **N** | **Transition** | | **Safety**  **Outcome type** | | **Domain** | | **Number of**  **items** | | **Dimensions** | | **Target population** | | **Tool administration** Time between transition and measurement | | **Methodological observations** | |  |  |
| **Patient perception** | | | | | | | | | | | | | | | | | | | | |
| ***Aller, 2013***  Spain  Response rate 23%  Mean age 56  Gender (% female) 56% | Continuity of care between care  levels (CCAENA) | 1500 | HOS<->GP | | HCP outcome | | Continuity of  care | | 29 | | 1) Patient-PCP relationship (Relational Continuity (7 items),  2) Patient-SCP relationship (Relational Continuity) (7 items),  3) Continuity across care levels (IC and MC) (11 items),  4) Accessibility across care levels (MC) (4 items) | | Patients that have  experienced a  transition | | Face-to-face interviews at primary care centres or at home covering the 3 months prior to the interview | | - Relevant items excluded after factor analysis undermining initial content validity  - More details of validation in Spanish publication | |  |  |
| ***Berendsen, 2009***  The Netherlands  Response rate 65%  Mean age 54  Gender (% female) 60% | Consumer  Quality Index  Continuum of  Care (CQI-COC) | 1404 | GP->HOS | | HCP outcome | | Collaboration  between PCP  and hospital | | 22 | | 1) GP approach (6 items),  2) GP referral (5 items),  3) Specialist (9 items),  4) Collaboration (2 items) | | Referred patients | | Means of dispersion unclear (post?). Covering transitions in the last 2 years | | - Many items excluded  - After extensive item reduction, the resulting item set did not fully cover the original content | |  |  |
| ***Coleman, 2002***  USA  Response rate NR  Mean age NR  Gender (% female) NR | Care Transition  Measure (CTM) | 60 | HOS->GP | | HCP outcome | | Quality of care  transition | | NR | | 1) Information transfer  2) Patient and caregiver preparation,  3) Support for self-management,  4) Empowerment to assert preferences | | Patients ≥65 years recently discharged from hospital and who received subsequent skilled nursing care in a facility/home | | Telephone survey covering ‘recent transitions’ | | - No reported total of items  - No reported number and characteristics of respondents | |  |  |
| ***Coleman, 2005***  USA  Response rate 99.5%  Mean age 67  Gender (% female) 60% | Care Transition  Measure (CTM) | 200 | HOS->GP | | HCP +  patient outcome | | Quality of care  transition | | 15 | | 1) Critical understanding (6 items),  2) Preferences important (3 items),  3) Management preparation (4 items),  4) Care plan (2 items) | | Adult patients discharged with primary diagnosis of chronic obstructive pulmonary disease, congestive heart failure, stroke, or hip fracture | | Telephone interview by trained survey researchers covering dischs=arge in the last 6-12 weeks | | - Sequel to Coleman, 2002  - All items treated as unidimensional measurement tool, although the questionnaire was developed with 4 dimensions | |  |  |
| ***Graumlich, 2008***  USA  Response rate NR  Mean age 54  Gender (% female) 58% | B-prepared | 460 | HOS->GP | | HCP  outcome | | Patient  perceptions of  preparedness  for hospital  discharge | | 11 | | 1) Self-care information for medication and activity (4 items),  2) Equipment and services (4 items),  3) Confidence (3 items) | | Adult patients discharged by internal medicine hospitalists | | Two telephone interviews conducted by trained researchers 1 week and 1 month after discharge | | - Sequel to Grimmer, 2001  - Very selective group of patients, limiting generalizability | |  |  |
| ***Grimmer, 2001***  Australia  Response rate patients  60%; carers 52%  Mean age NR  Gender (%female) NR | PREPARED | Patients  500,  Carers  431 | HOS->GP | | HCP +  patient  outcome | | Quality of  discharge  planning  activities | | 21 | | 1) Information exchange (5 items),  2) Receipt of medication information (4 items),  3) Preparation for coping post dis-charge (3 items), 4) Control of post-discharge circumstances (2 items),  5) Patient outcomes (3 items),  6) Cost and service usage (4 items) | | Patients ≥65 years, recently discharged from hospital | | Paper survey sent by post shortly after discharge | | - No patient characteristics reported  - Low factor loadings ignored in conclusion | |  |  |
| ***Hadjistavropoulos, 2008***  Canada  Response rate NR  Mean age 65  Gender (%female) 60% | Patient Continuity of Care Questionnaire (PCCQ) | 204 | HOS->GP | | HCP outcome | | Continuity of care at discharge | | 27 | | 1. 1) Relationships in hospital (7 items) 2. 2) Information transfer (6 items) 3. 3) Relationships in community (4 items) 4. 4) Management of forms (3 items) 5. 5) Management of follow-up (3 items) 6. 6) Management of communication (4 items) | | Adult patients recently discharged form hospital | | Means of dispersion unclear (post?). sent after 4 weeks after discharge. Possible help from research team. | | - Relevant items excluded because >5% of patients found it not applicable | |  |  |
| ***Haggerty, 2011***  Canada  Response rate 54-99%*  Mean age 48  Gender (% female) 65% | Several existing (validated)  questionnaires on continuity of care  with a  dimension on  management  continuity:  PCAS, PCAT-S,  CPCI, VANOCSS^#^ | 236-  427^¥^ | HOS<->GP | | HCP  outcome | | Management  continuity | | 28 in 4  questionnaires | | 1) PCAS: Integration (6 items),  2) PCAT-S: Coordination (4 items),  3) CPCI: Coordination of care (8 items),  4) VANOCCS: overall coordination (6 items), specialty access (4 items) | | Primary care patients who had seen more than one provider in the previous month | | Paper survey sent by post on  1) all transitions ever experienced,  2) all transitions ever experienced,  3) all transitions ever experienced,  4) Transitions in the last 12 months | | - Compared single dimensions on management continuity of related questionnaires, so  COSMIN not applicable | |  |  |
| ***Haggerty, 2012***  Canada  Response rate 80%  Mean age 53  Gender (% female) 71% | Patient  Perceived  Continuity from  Multiple  Clinicians | 256 | HOS<->GP | | HCP +  patient outcome | | Continuity of  care | | 37 | | 1) Coordinator role (5 items),  2) Comprehensive knowledge of patient (4 items),  3) Confidence and partnership (3 items),  4) Confidence in team (2 items),  5) Role clarity and coordination (6 items),  6) Information between clinicians (6 items),  7) Evidence of a care plan (7 items),  8) Self-management information provided (4 items) | | Adult patients in primary care seeing other clinicians in a variety of settings | | Paper self-administered survey in waiting rooms of primary care clinics on continuity of care in the last 12 months | | - Sequel to Haggerty 2011  - Use of a reference standard not applicable in transitional patient safety | |  |  |
| ***Kollen, 2010***  The Netherlands  Response rate 65%  Mean age 56  Gender (% female) 60% | Consumer Quality Index Continuum of Care (CQI-COC) | 1404 | GP->HOP | | HCP  outcome | | Quality of  continuum of  care | | 22 | | 1) GP approach (6 items),  2) GP referral (5 items),  3) Specialist (9 items),  4) Collaboration (2 items) | | Adult patients who had been referred and visited a specialist | | Means of dispersion unclear (post?). Covering transitions in the last 2 years | | - Sequel to Berendsen 2009 | |  |  |
| ***Uijen, 2011***  The Netherlands  Response rate 72%  Mean age 65  Gender (% female) 54% | Nijmegen  Continuity  Questionnaire | 288 | HOS<->GP | | HCP outcome | | Continuity of  care | | 28 | | 1) Personal continuity: care provider knows me (10 items),  2) Personal continuity: care provider shows commitment (6 items),  3) Team/cross boundary continuity (12 items) | | Patients with comorbidity | | Paper surveys distributed at their practice by GP trainees to 30 patients and returned by post covering transitions in the previous year | | - Relevant items excluded after factor analysis, undermining initial content validity | |  |  |
| ***Uijen, 2012***  The Netherlands  Response rate 76%  Mean age in GP 66, in  hos 58  Gender (% female) in GP  54%, in hos 49% | Nijmegen  Continuity  Questionnaire | 268 | HOS<->GP | | HCP  outcome | | Continuity of  care | | 28 | | 1) Personal continuity: care provider knows me (10 items),  2) Personal continuity: care provider shows commitment (6 items),  3) Team/cross boundary continuity (12 items) | | Patients with comorbidity | | Paper surveys distributed at their practice by GP trainees to 30 patients and returned by post covering transitions in the previous year | | - Sequel to Uijen, 2011  - Assessing discriminative validity  - Confirmatory factor analysis would be more appropriate than exploratory factor analysis | |  |  |
| **HCP perspective** | | | | | | | | | | | | | | | | | | | | |
| ***Author/Year***  Country  Response rate  Mean age  Gender | **Measurement tool** | **N** | | **Transition** | | **Safety outcome type** | | **Domain** | | **Number of items** | | **Dimensions** | | **Target population** | | **Tool administration**  Time between transition and measurement | | **Methodological observations** | |  |
| ***Berendsen, 2010***  The Netherlands  Response rate 45% (GP  47%, spec 44%)  Mean age GP 50, hospital specialist 51  Gender (% female) GP  33%, spec 21% | Doctors' opinions on collaboration (DOC-)  Questionnaire | 496 | | HOS<->GP | | Climate +  HCP  Outcome | | Quality of  interprofessional  collaboration | | 20 | | 1) Organisation (7 items),  2) Communication (3 items),  3) professional expertise (4 items),  4) image (3 items),  5) knowing each other (3 items) | | GPs and specialists | | Paper survey sent by post on current practice | | - Confirmatory factor analysis would be more appropriate than exploratory factor analysis | |  |
| ***Forster, 2012***  Canada  Response rate NA  Mean age NA  Gender (% female) NA | Peer review  process of  adverse  outcome | § | | HOS->GP | | Patient  outcome | | Adverse events at  discharge | | NA | | NA | | NA | | Web based application to rate adverse outcomes of recent transitional adverse events | | - COSMIN not applicable | |  |
| ***Graumlich, 2008***  USA  Response rate 76%  Mean age NR  Gender (% female) NR | Modified  Physician-  PREPARED | 417 | | HOS->GP | | HCP  outcome | | Quality of  hospital discharge | | 8 | | 1) Adequacy of discharge plan/Transmission (6 items),  2) Timeliness of communication (2 items) | | Community physicians | | Survey sent by e-mail to designated outpatient primary care professional, 10 days after discharge | | - No characteristics of respondents reported  - Relevant items excluded after factor analysis, undermining initial content validity | |  |
| ***Hess, 2009***  USA  Response rate NR  Mean age 46  Gender (% female) 20% | CRP-PIM:  Communication  with Referring  Physicians  Practice  Improvement  Module | 12212 | | GP->HOS | | Climate +  HCP outcome | | Communication  of consultants | | 13 | | 1) contacting/ communication with the consultant (10 items),  2) consultant office staff (3 items) | | Referring physicians | | Internet or telephone survey on communication in 12 months prior to the survey | | - No characteristics of respondents reported  - Generalizability theory used, but not described in publication | |  |
| ***Nuno-solinis, 2013***  Spain  Response rate 16%  Mean age 45  Gender (% female) 77% | Unnamed | 187 | | HOS<->GP | | Climate | | Interprofessional  collaboration | | 10 | | 1) personal relationship (4 items),  2) organisational setting (6 items) | | Clinical professionals (doctors and nurses) working in integrated healthcare organisations | | Electronic survey sent by e-mail on current practice | | - Based on a theoretical model  - Exploratory and confirmatory factor analysis used in the same sample | |  |
| ***Smith, 2004***  USA  Response rate NA  Mean age NA  Gender (% female) NA | Medication  discrepancy  tool | ǂ | | HOS->GP | | Patient outcome | | Medication discrepancies | | 27 | | 1) patient level (8 items),  2) system level (11 items),  3) resolution (8 items) | | Practitioners across the continuum of care | | Vignette study, means distribution unclear (face-to-face?) | | - COSMIN not applicable | |  |

SP: Spain; NL: the Netherlands; USA: United States of America; AUS: Australia; CAN: Canada; HOS: hospital; PCP: Primary care provider, NA= not applicable, NR=not reported, GP=general practitioner

* PCAS:79%, PCAT-S:91%, CPCI:99%, VANOCCS:64+54%

¥ PCAS:342, PCAT-S:392, CPCI:427, VANOCCS:278+136

§ Vignette study on 319 case reports, 30 physicians

ǂ Vignette study on 20 cases, 6 clinicians

# Primary Care Assessment Survey (PCAS), the Primary Care Assessment Tool – Short Form (PCAT-S), the Components of Primary Care

Instrument (CPCI) and the Veterans Affairs National Outpatient Customer Satisfaction Survey (VANOCSS)
