# Supplementary material for: Measurement tools and outcome measures used in transitional patient safety; a systematic review
Source: PLoS One. 2018 Jun 4;13(6):e0197312. doi: 10.1371/journal.pone.0197312 (PMC5986135; doi:10.1371/journal.pone.0197312)
Supplement: S2 Table — As some publications measured more than one outcome or one outcome from both the perspective of the healthcare professional and the patient, the added numbers can exceed the total. HCP = healthcare professional, PCP = Primary care practitioner. Many studies used more than one outcome, and from different perspectives. Therefore, the sub-totals can add up to more than the total outcomes. *Brain natriuretic peptide (BNP), Hemoglobine A1c, lipid levels, volume status, renal function, weight, International Normalized Ratio (INR), haemorrhagic events, thromboembolic events, post referral colonoscopy delay. (DOCX) [file pone.0197312.s005.docx]

**S4 Table. Outcome inventory: used outcomes from healthcare professionals’ and patients’ perspectives inventoried in 191 publications that measured transitional patient safety: which are used and how often.**
As some publications measured more than one outcome or one outcome from both the perspective of the healthcare professional and the patient, the added numbers can exceed the total.

| **Used outcomes in 191 publications** | Total (n) | HCP perspective  (n) | Patient perspective  (n) |
| --- | --- | --- | --- |
| **Medication total** | **98** | **92** | **19** |
| Adverse drug events, medication errors and discrepancies | 97 | 92 | 18 |
| Medication alterations during transfer | 3 | 2 | 1 |
| **Admissions and readmission total** | **66** | **53** | **30** |
| Readmission | 55 | 44 | 22 |
| Admissions and inpatient care use | 13 | 9 | 8 |
| Drug-related hospital (re) admission | 7 | 7 | 0 |
| **Patient and caregiver total** | **58** | **16** | **46** |
| Physical and psychosocial outcomes | 28 | 2 | 28 |
| Patient satisfaction | 23 | 0 | 23 |
| Patient care experiences | 18 | 0 | 18 |
| Patient knowledge | 19 | 5 | 14 |
| Patient adherence | 15 | 6 | 9 |
| Patient- HCP communication | 4 | 3 | 2 |
| Caregiver burden | 2 | 0 | 2 |
| Patient care needs | 2 | 1 | 1 |
| Caregiver satisfaction | 1 | 0 | 1 |
| Patient empowerment | 1 | 1 | 0 |
| **Unscheduled visits total** | **40** | **28** | **20** |
| Emergency department visit | 33 | 21 | 17 |
| PCP follow up | 13 | 9 | 7 |
| Unscheduled hospital or PCP visit | 5 | 4 | 2 |
| **Healthcare use total** | **33** | **24** | **13** |
| Overall healthcare use | 13 | 9 | 7 |
| Length of hospital stay | 12 | 11 | 2 |
| Visits to primary care | 4 | 1 | 3 |
| Number of contacts with case managers | 1 | 1 | 0 |
| Number of transitions | 1 | 1 | 1 |
| Redundant diagnostic testing | 1 | 1 | 0 |
| Number of patients with direct phone contact | 1 | 1 | 0 |
| **HCP total** | **37** | **36** | **3** |
| HCP satisfaction and opinions | 20 | 20 | 0 |
| Interprofessional collaboration and communication | 11 | 10 | 3 |
| HCP attitudes | 6 | 6 | 0 |
| HCP confidence and skills | 5 | 5 | 0 |
| HCP knowledge | 4 | 4 | 0 |
| HCP awareness | 3 | 3 | 0 |
| Guideline use | 3 | 3 | 0 |
| HCP: barriers and facilitators | 2 | 2 | 0 |
| Patient safety culture | 2 | 2 | 0 |
| Productivity | 2 | 2 | 0 |
| Patient centeredness | 1 | 1 | 0 |
| **Harm and adverse events** | **34** | **24** | **17** |
| **Discharge communication total** | **26** | **26** | **0** |
| Quality and timeliness of discharge summary | 24 | 24 | 0 |
| Diagnostic test follow up | 4 | 4 | 0 |
| **Costs** | **21** | **21** | **7** |
| **Mortality total** | **17** | **17** | **0** |
| All-cause mortality | 16 | 16 | 0 |
| Cause-specific mortality | 2 | 2 | 0 |
| **Questionnaires on transitional patient safety** | **16** | **4** | **13** |
| Care transition measure (CTM) (Coleman et al.) | 9 | 0 | 9 |
| Continuity of care between care Levels (CCAENA) (Aller et al.) | 2 | 0 | 2 |
| Collaboration questionnaire (Nuno Solinis et al.) | 1 | 1 | 0 |
| DOC-questionnaire (Berendsen et al.) | 1 | 1 | 0 |
| B-prepared (Graumlich et al.) | 1 | 0 | 1 |
| Physician prepared (Graumlich et al.) | 1 | 1 | 0 |
| Patient Percieved Continuity from Multiple Clinicians (Haggerty et al.) | 1 | 0 | 1 |
| Medication discrepancy tool (Smith et al.) | 1 | 1 | 0 |
| **Questionnaires: related concepts and non-validated questionnaires** | **15** | **10** | **6** |
| **Specific disease-related outcomes*** | **9** | **9** | **0** |
| **Referrals total** | **5** | **5** | **0** |
| Referral: timeliness and inappropriateness | 4 | 4 | 0 |
| Re-referrals and referral rate | 2 | 2 | 0 |
| **Intervention total** | **5** | **5** | **1** |
| Implementation (number/ quality) | 3 | 3 | 0 |
| Feasibility intervention/ user friendliness | 2 | 2 | 1 |

HCP= healthcare professional, PCP=Primary care practitioner. Many studies used more than one outcome, and from different perspectives. Therefore the sub-totals can add up to more than the total outcomes.
*Brain natriuretic peptide (BNP), Hemoglobine A1c, lipid levels, volume status, renal function, weight, International Normalized Ratio (INR), haemorrhagic events, thromboembolic events, post referral colonoscopy delay.
