# Supplementary material for: Measurement tools and outcome measures used in transitional patient safety; a systematic review
Source: PLoS One. 2018 Jun 4;13(6):e0197312. doi: 10.1371/journal.pone.0197312 (PMC5986135; doi:10.1371/journal.pone.0197312)
Supplement: S3 Table — ADE = Adverse drug event; AE = Adverse event; DOC-questionnaire = Doctors’ Opinions on Collaboration-questionnaire; ED = Emergency department; GP = General practitioner; HCP = Healthcare professional; PCP = Primary care physician; RCT = Randomised controlled trial. (DOCX) [file pone.0197312.s006.docx]

| **Author/ Year** | **Transition** | **Safety outcome level** | **Construct** | **Aim/ research question** | **Target population** | **Design** | **Intervention** | **Perspective/ Reported by** | **Used outcomes** |
| --- | --- | --- | --- | --- | --- | --- | --- | --- | --- |
| Ahmed 2005 [1]  UK  N=208 | PCP<->HOS Overall continuity | Patient outcome | Medication safety | To bridge the information gap that exists between the primary and secondary care continuum with regard to the swift transfer of accurate patient medication details using the interventions of an intervention pharmacist. | Patients visiting hospital | Intervention study: before- after design | Medication compared between primary care and secondary care. | HCP | ADEs, medication errors and discrepancies; Medication alterations during transfer |
| Akbarov 2015 [2]  UK  N= 205,519 | PCP<->HOS Overall continuity | Patient outcome | Medication safety | To investigate the feasibility of linked primary and secondary care electronic health record data for surveillance of medication safety, examining not only prescribing but also monitoring, and associations with patient- and general practice-level characteristics. The objectives of this study included assessing the prevalence of 22 medication safety indicators, investigating associations with patient and practice characteristics, and investigating variation between general practices. | All adult patients of general practices | Observational study: cross-sectional design |  | HCP | ADEs, medication errors and discrepancies |
| Alassaad 2011 [3]  Sweden  N=290 | HOS->PCP Discharge | Patient outcome | Medication safety | To investigate the frequency, type and severity of prescribing and transcribing errors for drugs dispensed in multidose plastic packs when patients are discharged from the hospital. The secondary objective was to correct identified errors and suggest measures to promote safe prescribing. | Adult patients with Multi drug dosage = baxter | Intervention study: prospective design | All multi drug dosage orders were reconciled and transcribing errors communicated to the prescribing physician. | HCP | ADEs, medication errors and discrepancies |
| Alex 2016 [4]  USA Intervention N=145, Control N=134 | HOS<->PCP Admission and discharge | Patient outcome | Medication reconciliation | To determine whether collaboration between a clinical pharmacist and inpatient physicians can improve the discharge process and resolve medication discrepancies at discharge. | All patients admitted to a veteran hospital | Intervention study: non-randomized controlled trial | A pharmacist assisted with medication management, medication reconciliation, and medication education upon discharge. | HCP | ADEs, medication errors and discrepancies |
| Alidina 2016 [5]  USA  N=13 practices | HOS<->PCP Overall continuity | HCP outcome | Coordination of care | To explore how patient centred medical homes use coordination mechanisms to build and optimize their medical neighbourhoods. | Patient-centred medical home physicians | Intervention study: before- after design | Implementation of a patient centred medical home | HCP | HCP: barriers and facilitators;  HCP satisfaction and opinions;  HCP attitudes |
| Aller 2012  [6]  Spain  N=1500 | PCP<->HOS Overall continuity | HCP outcome | Continuity of care | To analyse patient’s reported elements of relational, informational and managerial (dis)continuity between primary and outpatient secondary care and to identify associated factors. |  | Observational study: cross-sectional design |  | Patient | Patient care experiences;  Patient satisfaction; Continuity of care between care Levels (CCAENA) (Aller et al.) |
| Aller 2013  [7]  Spain  N= 1405 | PCP<->HOS Overall continuity | HCP outcome | Continuity of care | To determine the patients’ perceived degree of continuity of care between primary and secondary care and to identify contextual and individual factors that influence patients’ perceptions of continuity of care. | Adult patients who had received primary care and secondary care for the same condition | Observational study: cross-sectional design |  | Patient | Patient care experiences;  Patient satisfaction; Continuity of care between care Levels (CCAENA) (Aller et al.) |
| Anderson 2013 [8]  USA  N= 470 | HOS->PCP Discharge | HCP & patient outcome | Medication reconciliation | To examine the feasibility and characteristics that define successful implementation of a Clinical Pharmacy Specialist telephonic hospital discharge follow-up quality improvement initiative, as well as the impact of this initiative. | Adult patients with a PCP and discharged from the hospital medical and surgical services | Intervention study: retrospective design | Telephonic discharge follow-up by residency-trained Clinical Pharmacy Specialists within 48 to 96 h of hospital discharge. | Patient | Readmission;  PCP follow up |
| Armor 2016 [9]  USA N=43 | HOS->PCP Discharge | Patient outcome | Medication reconciliation | To evaluate ADEs, potential ADEs and medication discrepancies occurring between hospital discharge and primary care follow-up. | Adult patients recently discharged from the hospital | Observational study: retrospective design | Medication reconciliation conducted by a pharmacist after discharge. | HCP | ADEs, medication errors and discrepancies; Readmission;  Harm and adverse events |
| Arora 2010  [10]  USA  Patient N=64, PCP n=40 | HOS->PCP Discharge | HCP outcome | Discharge safety and experience | To report older patients’ experiences with problems after hospital discharge and investigate whether PCP were aware of their hospitalization. | Frail, older patients hospitalized patients and their primary care practitioners. | Observational study: prospective design |  | HCP & patient | HCP awareness; Interprofessional collaboration and communication; Patient care experiences |
| Atkinson 2005  [11]  USA  N=1006 | PCP<->HOS Overall continuity | Patient outcome | Medication safety | To describe a successful initiative to implement a pharmacist-centred medication management program into the existing care practices at Home Care Plus. | Elderly patients: with one or more of four risk factors for ADES | Observational study: prospective design | A pharmacist conducted a drug regimen review, assessing the patient for potential inappropriate medications, ADES, and negative health outcomes potentially related to medication use. | HCP | ADEs, medication errors and discrepancies |
| Azzi 2014  [12]  Australia N=300 | PCP->HOS Referral | Patient outcome | Medication safety | To identify, classify and determine the factors associated with medication discrepancies for type 2 diabetes mellitus patients, referred from primary care to a tertiary ambulatory clinic. | Adult type 2 diabetes mellitus patients first refereed to the Diabetes Centre. | Observational study: retrospective design |  | HCP | ADEs, medication errors and discrepancies |
| Balaban 2015 [13]  USA  N=1510 | HOS->PCP Discharge | Patient outcome | Case management at discharge | To determine if an intervention by patient navigators, hospital-based Community Health Workers, reduces readmissions among high risk, low socioeconomic status patients. | High risk patients of general medicine with at least one readmission risk factors | Intervention study: RCT | Patient navigators provided coaching and assistance in the transition from hospital to home (discharge preparation, telephone outreach, supporting medication management, scheduling of follow-up appointments, communication with primary care, and symptom management). | HCP & patient | Readmission;  PCP follow up;  ED visits |
| Baldo 2014  [14]  Italy  N=3568 | PCP<->HOS Overall continuity | Patient outcome | Continuity of diabetes care (care chain) | To assess mortality of patients with type2 diabetes by type of healthcare delivery system, i.e. through specialist centres or generalist doctors, or integrated care. | Adult patients with diabetes treated at either a diabetes clinics, the GP, or receiving integrated care | Observational study: retrospective design |  | HCP | All-cause mortality; Cause-specific mortality |
| Balling 2015  [15]  USA  N=1011 | HOS->PCP Discharge | Patient outcome | Medication reconciliation | To assess the impact of a transition-of-care pharmacist during hospital discharge. | Patients discharged from two inpatient units (adult medical/surgical) with high readmission rates. | Observational study: prospective design | A transition-of-care pharmacist to provided discharge medication reconciliation and patient education on discharge medications. | HCP | ADEs, medication errors and discrepancies; Readmission |
| Balogun 2015 [16] USA  N=251 | PCP<->HOS Overall continuity | HCP outcome | Interprofessional communication | To enable students to discuss the roles of physicians and nurses in transitioning patient care from one clinical setting to another and to demonstrate competence in interprofessional communication skills and strategies in transitions of care. | Medical and nursing students | Intervention study: before after design | An interprofessional workshop on transitions in geriatric care. | HCP | HCP knowledge;  HCP confidence and skills |
| Bauer 2015 [17]  Germany  Sample size calculation: N=370 | HOS->PCP Discharge | Patient outcome | Care transitions in colorectal care | To increase the proportion of patients achieving a clinical relevant improvement of their health-related quality of life. | Adults with colorectal cancer in curative therapy conditions | Intervention study: RCT (protocol) | Additional support by specialized oncology nurses after discharge by telephone, consisting of symptom monitoring, counselling on self-assessment and self-management and dealing with individual resources for coping and psychosocial well-being. | Patient | Physical and psychosocial outcomes;  Patient care needs |
| Belda-Rustarazo 2015  [18]  Spain  N=814 | PCP<->HOS Admission and discharge | Patient outcome | Medication safety | To examine the frequency/type of reconciliation errors at hospital admission and discharge and to report on the drugs involved, associated risk factors and potential to cause harm in a healthcare setting with comprehensive digital health records. | Elderly patients with polypharmacy, admitted to the Internal Medicine Department | Observational study: prospective design |  | HCP | ADEs, medication errors and discrepancies;  Harm and adverse events |
| Berendsen 2009  [19]  the Netherlands  N=491  (259 GPs, 232 Hos spec) | PCP<->HOS Overall continuity | Climate & HCP outcome | Communication between GP and hospital | To address the following research question. How do GPs and specialists assess their mutual communication through telephone, letters and postgraduate courses? | GPs and specialists in the Netherlands | Observational study: cross-sectional design |  | HCP | Interprofessional collaboration and communication;  DOC-questionnaire (Berendsen et al.) |
| Bergkvist 2009  [20]  Sweden  N=172 | HOS->PCP Discharge | Patient outcome | Medication safety and quality of discharge communication | To investigate whether the process improves the quality of the discharge summary and if this process development also reduces medication errors when patients are discharged from hospital. | Elderly patients admitted to the Department of Internal Medicine | Intervention study: non-randomized controlled trial | Medication reconciliation by a pharmacist at admission and discharge. Based on identified problems, a systematic medication care plan was created. | HCP | ADEs, medication errors and discrepancies;  Quality and timeliness of discharge summary |
| Biese 2014  [21]  USA  N=120 | HOS->PCP Discharge form ED | Patient outcome | Case management at discharge from ED | To investigate whether a post-discharge telephone call–mediated intervention by a nurse would improve discharge care plan adherence, specifically by expediting post–ED visit physician follow-up appointments and/or compliance with medication changes. The second objectives were to determine if this telephone call intervention would reduce return ED visits and/or hospitalizations within 35 days of the index ED visit and to determine potential cost savings of this intervention. | Elderly patients discharged from an academic Level I trauma center ED | Intervention study: RCT | A telephone call from a trained study nurse between 1 and 3 days after ED discharge to review and facilitate compliance with patient-specific discharge instructions. | Patient | Medication alterations during transfer; PCP follow up; admission and inpatient care use;  ED visits;  Costs;  Patient adherence; Patient satisfaction; Patient knowledge |
| Bishop 2015  [22]  USA  N=104 | HOS->PCP Discharge | Patient outcome | Medication reconciliation | To determine whether integration of pharmacist review in the process of medication reconciliation at discharge identifies and corrects discrepancies. | Adult patients admitted and discharged with medication for Internal Medicine | Observational: study: prospective design | Discharge medication reconciliation by a pharmacist. | HCP | ADEs, medication errors and discrepancies |
| Bloom-Feshback 2016 [23] USA  N=101 | HOS->PCP Discharge | HCP outcome | Discharge safety in patients with low health literacy | To develop an Objective Structured Clinical Examination for medical students to demonstrate communication skills to be used during the hospital discharge process with patients of low health literacy. | Medical students | Observational study: prospective design | A health literacy workshop. | HCP | HCP knowledge;  HCP confidence and skills |
| Bonnet-Zamponi 2012  [24]  France  N=639 | HOS->PCP Discharge | Patient outcome | Medication safety | To assess the effect of an intervention on drug-related problem (DRP; adverse drug reactions, adherence problems, underuse)-related readmission rates in older adults. | Elderly patients admitted emergently to hospital | Intervention study: RCT | Multimodal discharge planning intervention: prescription review, patient education on disease self-management, enhanced Transition-of-Care Communication. | HCP | Readmission;  Drug-related hospital (re) admission |
| Braund 2014  [25]  New Zealand N=1374 | HOS->PCP Discharge | Patient outcome | Medication safety | To identify the types and quantities of drug related problems that community pharmacies detect within a single district health board in New Zealand. | All patients discharged with medication | Observational study: cross-sectional design |  | HCP | ADEs, medication errors and discrepancies;  Harm and adverse events |
| Bray-Hall 2010  [26]  USA  N=136 | HOS->PCP Discharge | Climate & HCP outcome | Education in transitional safety and skills | To develop a sustainable and effective Transition in Care Curriculum (TICC). Specific goals were to increase student confidence in and knowledge of skills necessary during care transitions at the time of hospital discharge, and to quantify the frequency of student-identified medication discrepancies during a post-discharge home visit. | Third year medical students | Intervention study: before- after design | Multiple instructional modalities, including didactics, self-study, experiential learning, and small group discussion. | HCP | ADEs, medication errors and discrepancies;  HCP knowledge;  HCP satisfaction and opinions;  HCP confidence and skills |
| Brener 2016 [27]  Canada  N= 164,059 | HOS->PCP Discharge | Patient outcome | Discharge safety | To determine the frequency of supportive-care visits by PCPs to hospitalized patients and to identify the association between these visits, patient outcomes, and health services utilization. | Adults hospitalized between 2008 to 2009 | Observational study: retrospective design |  | HCP | All-cause mortality; Readmission;  Visits to primary care; ED visit;  Patient care needs |
| Burns 2014  [28]  USA  N=423 | HOS->PCP Discharge | HCP & patient outcome | Case management at discharge | To pilot-test the feasibility and preliminary effect of a community health worker intervention to reduce hospital readmissions. | Adult patients with one or more risk factors for readmission | Intervention study: RCT | A community health worker facilitated the transitions from hospital to home and back to the primary care provider. Including: introductory visits with patients in the hospital; community health worker participation in the hospital discharge process; semi-structured community health worker outreach to elicit patient concerns; and liaison calls, as needed, to primary care nurses to assist in scheduling or to respond to patient concerns. | HCP | Readmission;  Visits to primary care; Admission and inpatient care use;  ED visits;  Number of contacts with case managers; HCP: barriers and facilitators |
| Callen 2008  [29]  Australia N=245 | HOS->PCP Discharge | HCP outcome | Discharge safety | To study the hospital discharge summaries, which were either handwritten or electronic, of a population of inpatients, with regard to documentation of information required for ongoing care; and to compare the electronic with the handwritten summaries concerning documentation of this information. | Discharge summaries (handwritten or electronic) of elderly patients hospitalised for at least two days. | Observational study: prospective design | An electronic discharge summary system with training. | HCP | Quality and timeliness of discharge summary |
| Callen 2009  [30]  Australia  N=1808 | HOS->PCP Discharge | Patient outcome | Medication safety at discharge | To quantify and compare the medication transcription error rate from handwritten medications on manual discharge summaries to typed medications on electronic discharge summaries, and to examine the quality of medication documentation according to the level of medical training of the doctors who created the discharge summaries. | Discharge summaries of elderly patients with polypharmacy discharged to private homes or a nursing home | Intervention study: before- after design | An electronic discharge summary system. | HCP | ADEs, medication errors and discrepancies |
| Carter 2008  [31]  USA  Sample size calculation: N=1000 | PCP<->HOS Admission and discharge | Patient outcome | Medication reconciliation | To determine the effects of hospital pharmacists’ enhanced communication with patients and their community providers are described. | Adult patients admitted  with a diagnosis of hypertension, hyperlipidaemia, heart failure, coronary artery disease, myocardial infarction, stroke, transient ischemic attack, asthma, chronic obstructive pulmonary disease, or diabetes or receives oral anticoagulation. | Intervention study: RCT | A pharmacist case manager provided admission medication verification with the patients’ community pharmacists, medication teaching, and discharge counselling. | HCP & patient | ADEs, medication errors and discrepancies; Readmission;  Visits to primary care; Admission and inpatient care use; Unscheduled hospital or PCP visit;  ED visits;  Costs;  Guideline use;  Patient adherence; Harm and adverse events |
| Cavanaugh 2015  [32]  USA  N=124 | HOS->PCP Discharge | Patient outcome | Case management at discharge | To compare the transitions of care, measured by hospital readmission rates and medication interventions, in a multidisciplinary team visit coordinated by a clinical pharmacist with those conducted by a physician-only team in a hospital follow-up program. | Discharged patients | Observational study: retrospective design | A multidisciplinary team visit coordinated by a pharmacist in a hospital follow-up program. | HCP | ADEs, medication errors and discrepancies; Medication alterations during transfer; Readmission;  Patient adherence |
| Chan 2015 [33]  USA  N=685 | HOS->PCP Discharge | Patient outcome | Discharge safety | To assess the effect of the support from hospitalization to home for elders intervention on the patient experience. | Patients 55 years and older, admitted to the internal medicine, family medicine, cardiology, or neurology services and discharged home | Intervention study: RCT | Intervention participants received inpatient visits by a language-concordant study nurse and post-discharge phone calls from a language-concordant nurse practitioner to reinforce the care plan and to address acute complaints. | HCP & patient | Patient- HCP communication; Patient care experiences;  Patient knowledge; Implementation (number/ quality); Care transition measure (CTM) (Coleman et al.) |
| Chevalier 2006  [34]  Canada  N=39 | HOS->PCP Discharge | Climate & HCP outcome | Medication safety at discharge | To measure nurses’ perceptions of patient safety, medication safety and current medication reconciliation practice at transition points in a patient’s hospital stay. This study provided baseline information on nurses’ perceptions of patient safety culture and medication safety at transition points in a patient’s hospital stay. | Nursing staff in three general medicine units | Observational study: cross-sectional design |  | HCP | ADEs, medication errors and discrepancies;  Patient safety culture; Questionnaires: related concepts and non-validated questionnaires |
| Coleman 2004a  [35]  USA  N=1393 | HOS->PCP Discharge | HCP & patient outcome | Patient empowerment | To test whether an intervention designed to encourage older patients and their HCPs to assert a more active role during care transitions can reduce rehospitalisation rates. | Community dwelling elderly patient | Intervention study: non-randomized controlled trial | A personal health record and a series of visits and telephone calls with a transition coach. | HCP % patient | Admission and inpatient care use;  ED visits;  Patient care experiences |
| Coleman 2004b  [36]  USA  N=1404 | HOS->PCP Discharge | Patient outcome | Discharge safety | To describe patterns of post hospital care transitions; characterize these patterns as uncomplicated or complicated; identify those at greatest risk for complicated transitions. | Elderly patients discharged from an acute care hospital | Observational study: cross-sectional design |  | HCP & patient | Number of transitions;  Overall healthcare use;  Patient care experiences;  Patient satisfaction; Physical and psychosocial outcomes; Questionnaires: related concepts and non-validated questionnaires |
| Coleman 2005  [37]  USA  N=375 | HOS->PCP Discharge | Patient outcome | Medication safety at discharge | To determine the prevalence and contributing factors associated with post hospital medication discrepancies. | Elderly, community dwelling adults admitted to the hospital | Observational: study prospective design |  | HCP | ADEs, medication errors and discrepancies; Medication discrepancy tool (Smith et al.) |
| Coleman 2006  [38]  USA  N=750 | HOS->PCP Discharge | Patient outcome | Case management at discharge and patient empowerment | To tests whether this same intervention, designed to encourage older patients and their HCPs to assert a more active role in their care transitions, can reduce rates of rehospitalisation. | Community dwelling, elderly patients admitted for a non-psychiatric condition | Intervention study: RCT | Tools for patients to promote cross-site communication and encouragement to take a more active role in their care and to assert their preferences and guidance from a “transition coach.” | HCP | Readmission |
| Dalal 2014  [39]  USA  N=441 | HOS->PCP Discharge | HCP outcome | Post-discharge test continuity | To evaluate the impact of our automated email notification system on physician awareness of a tests pending at discharge results and assess overall satisfaction with this strategy. | Adult patients, discharged from general medicine or cardiology | Intervention study: RCT | An email system that emailed the hospital and network PCP when a patient’s test was pending at discharge | HCP | HCP awareness;  HCP satisfaction and opinions |
| Daley 2010  [40]  USA  Intervention N=89, Control N=375 | HOS->PCP Discharge | Patient outcome | Medication reconciliation | To test the effect of a transitional heart failure programme. | Patients with heart failure, who were admitted with a diagnosis of heart failure and are at high risk for readmission. | Intervention study: non-randomized controlled trial | Health literacy screening, medication reconciliation upon hospital admission and discharge, communication of discharge medication to all healthcare providers, and a follow-up appointment with the patient’s cardiologist post-discharge | HCP | All-cause mortality; Readmission;  Length of hospital stay;  Costs |
| Dichmann Sorknaes 2016 [41]  Denmark  N=266 | HOS->PCP Discharge | Patient outcome | Discharge safety | To investigate the effect of one week of daily real-time telemedicine video consultations (teleconsultation) between hospital-based nurses specialized in respiratory diseases (telenurses) and patients with severe Chronic obstructive pulmonary disease discharged in addition to conventional treatment compared to the effect of conventional treatment. | Patients discharged after admission with acute exacerbation of chronic obstructive pulmonary disease | Intervention study: RCT | One week of daily real-time telemedicine video consultations  (teleconsultation) between hospital-based nurses specialized in respiratory diseases (telenurses) | HCP | Readmission |
| Donaho 2015 [42]  USA  N=114 | HOS->PCP Discharge | HCP & patient outcome | Discharge safety | To reduce 30-day readmission rates to the hospital. | Patients recently admitted to the hospital with a  diagnosis of HF | Observational study: prospective design | Providing timely, protocol-driven  post-discharge evaluation and management, including a discharge plan, medication education and reconciliation, education on symptom recognition and reporting, and coordination of outpatient health care  resources. Care coordination services included communications with  the primary care provider, other specialist providers, and/or home health services. | HCP | ADEs, medication errors and discrepancies; Readmission;  Volume status;  Costs |
| Douglas 2013  [43]  Canada  N=47 | HOS->PCP Discharge | Patient outcome | Discharge safety | To determine whether two pre discharge functional measures were valid for predicting time to incident or harm after discharge. | Elderly patients recruited from a geriatric rehabilitation unit and referred to occupational therapy and undergoing functional or cognitive assessment | Observational study: prospective design |  | HCP & patient | Visits to primary care; Admission and inpatient care use;  Harm and adverse events;  Physical and psychosocial outcomes; Questionnaires: related concepts and non-validated questionnaires |
| Dunn 2015  [44]  USA  N=797 | HOS->PCP Discharge | HCP outcome | Continuity of anticoagulant care (care chain) | To develop a concise report to improve the timeliness of international normalized ratio (INR) testing and quality of warfarin management post hospitalization. | Patients who were discharged on warfarin | Intervention study: before- after design | Safe Transitions Anticoagulation Report (STAR), which contains essential information on anticoagulation and is embedded in the discharge summary. | HCP | Diagnostic test follow up;  Specific disease-related outcomes;  HCP satisfaction and opinions |
| Dutton 2003  [45]  UK  N=1247 | PCP->HOS Admission | Patient outcome | Medication reconciliation | To quantify prescribing errors to pre-admission medication in patients admitted to hospital. It also assesses the impact of a hospital pharmacies in identifying and correcting these errors | Patients admitted to hospital | Intervention study: before- after design | Medication reconciliation | HCP | ADEs, medication errors and discrepancies;  Harm and adverse events |
| Elliot 2012  [46]  Australia N=428 | PCP<->HOS Admission and discharge | HCP & patient outcome | Medication reconciliation | To test the impact of a hospital pharmacist-prepared interim residential care medication administration chart (IRCMAC) on medication administration errors and use of locum medical services after discharge from hospital to residential care. | Patients discharged with medication changes | Intervention study: before- after design | Medication reconciliation using software | HCP | ADEs, medication errors and discrepancies;  HCP satisfaction and opinions |
| Ellit 2010  [47]  Australia  N=76 | HOS->PCP Discharge | Patient outcome | Medication safety at discharge | To reconcile patients’ medicines and to classify drug related problems identified during medication review conducted after discharge from hospital. | Patients discharged home from the cardiology unit  with cardiac medication | Observational study: retrospective design |  | HCP | ADEs, medication errors and discrepancies; Questionnaires: related concepts and non-validated questionnaires |
| Eskildsen 2011  [48]  USA  N=121 | HOS->PCP Discharge | Climate & HCP outcome | Education in transitional safety and skills | To give medical students the fund of knowledge and skills to develop and implement a safe discharge plan. | Fourth-year medical students | Intervention study: before- after design | A care transitions curriculum: discussion of an online case highlighting care transitions issues; preparation of a discharge summary based on online templates; and a post-discharge phone call to one of their patients. | HCP | Quality and timeliness of discharge summary;  HCP knowledge;  HCP satisfaction and opinions;  HCP confidence and skills;  HCP attitudes |
| Farris 2014  [49]  USA  N=945 | HOS->PCP Discharge | Patient outcome | Medication reconciliation | To determine if a pharmacist case manager providing a faxed discharge medication care plan from a tertiary care institution to primary care could improve medication appropriateness and reduce adverse events, rehospitalisation and ED visits. | Discharged patients | Intervention study: RCT | Medication reconciliation on admission and discharge, patient education. | HCP | ADEs, medication errors and discrepancies; Readmission;  Overall healthcare use;  Unscheduled hospital or PCP visit;  ED visits;  Harm and adverse events |
| Fertig 1993  [50]  UK  N=31 practices | PCP->HOS Referral | Patient outcome | Referral safety | To determine the extent to which variation in rates of referral among GPs may be explained by inappropriate referrals and to estimate the effect of implementing referral guidelines. | General practices in the UK | Observational study: prospective design |  | HCP | Re-referrals and referral rate;  Referral: timeliness and inappropriateness |
| Flink 2015  [51]  Sweden  N=76 medical records of 22 patients | PCP<->HOS Overall continuity | Climate & HCP outcome | Continuity of care | To explore patient handovers between primary and secondary care by assessing the levels of patient-centeredness of medical records used for communication between care settings and by assessing continuity of patient care. | Adult patients with chronic diseases and/or polypharmacy | Observational study: retrospective design |  | HCP | Patient centeredness |
| Forster 2003  [52]  Canada  N=400 | HOS->PCP Discharge | Patient outcome | Discharge safety | To describe the incidence, severity, preventability, and “ameliorability” of adverse events affecting patients after discharge from the hospital and to develop strategies for improving patient safety during this interval. | Adult hospital patients | Observational study: retrospective design |  | HCP & patient | Harm and adverse events |
| Forster 2004  [53]  Canada  N=361 | HOS->PCP Discharge | Patient outcome | Discharge safety | To determine the risk, severity and type of adverse events after discharge from 2 campuses of a Canadian teaching hospital. | Patients discharged home or to residential living from general internal medicine | Observational study: retrospective design |  | HCP & patient | Harm and adverse events |
| Forster 2005  [54]  Canada  N=400 | HOS->PCP Discharge | Patient outcome | Discharge safety | To describe the incidence of ADEs, preventable ADEs, and ameliorable ADEs occurring after hospital discharge and their associated risk factors. | Patients discharged home from general internal medicine | Observational study: retrospective design |  | HCP & patient | ADEs, medication errors and discrepancies |
| Forster 2007  [55]  Canada  N=408 | HOS->PCP Discharge | Patient outcome | Discharge safety from the ED | To describe the types of adverse events in patients discharged home from an ED. | Patients who were sent home directly from the ED | Observational study: retrospective design |  | HCP & patient | All-cause mortality; Readmission;  ED visits;  Harm and adverse events;  Physical and psychosocial outcomes |
| Foust 2011  [56]  USA  N=198 hospital discharge medical records, representing 162 patients | HOS->PCP Discharge | Patient outcome | Medication safety at discharge | To examine the rates and types of medication reconciliation problems among older adults hospitalized for acute episodes of heart failure who were discharged home. | Adult patients hospitalized for acute episodes of heart failure who were discharged home | Observational study: retrospective design |  | HCP | ADEs, medication errors and discrepancies |
| Frydenberg 2012  [57]  Norway  N=30 | PCP<->HOS Admission and discharge | Patient outcome | Medication reconciliation | To explore whether such inadequate communication leads to errors in patients’ medication on admission, during hospital stay, and after discharge, and whether these errors were potentially harmful. | Patients urgently admitted to the medical ward, and using three or more drugs | Observational study: prospective design |  | HCP & patient | ADEs, medication errors and discrepancies |
| García-Molina Sáez 2016 [58]  Spain  N=321 | HOS->PCP Discharge | Patient outcome | Medication reconciliation | To analyse the effectiveness of a computerized pharmaceutical intervention to reduce reconciliation errors at discharge in Spain. | All patients admitted for at least 48 hours | Intervention study: before after design | Before patient discharge, the  pharmacist included the pre-admission medication in a computerized tool integrated into the electronic clinical history of the patient and designed to facilitate the task of medication reconciliation/prescription, and adding recommendations for discharge. | HCP | ADEs, medication errors and discrepancies |
| Gardella 2012  [59]  USA  N= 8586 | PCP<->HOS Admission and discharge | HCP & patient outcome | Medication safety and patient education of medication safety | To produce an accurate preadmission medication list within 24 hours of admission, and to provide post-discharge education of complex patients by pharmacists | Hospital patients | Intervention study: non-randomized controlled trial | Medication reconciliation at discharge and communication of discharge medication to the next provider. The reconciliation team included a hospitalists, non-hospitalist physicians, pharmacists, nurses, and personnel from marketing, information technology, and administration. | HCP | ADEs, medication errors and discrepancies; Readmission;  Drug-related hospital (re) admission;  PCP follow up;  ED visits;  Harm and adverse events;  Patient knowledge; Patients with Direct Phone Contact |
| Gardner 2014  [60]  USA  N=1240 | HOS->PCP Discharge | Patient & organisational outcome | Cost effectiveness of care transition intervention | To evaluate the cost avoidance associated with implementation of the Care Transitions Intervention (CTI). | Patients with planned discharges home | Intervention study: non-randomized controlled trial | A patient-centred coaching intervention to empower individuals to better manage their health. It begins in-hospital and continues for 30 days post-discharge, including one home visit and one to two phone calls. | HCP | Overall healthcare use;  Costs |
| Ghatnekar 2013  [61]  Sweden N=NA | PCP<->HOS Admission and discharge | HCP & patient outcome | Medication reconciliation | To evaluate the cost effectiveness of a multidisciplinary team including a pharmacist for systematic medication review and reconciliation from admission to discharge at hospital among elderly patients (the Lund Integrated Medicines Management (LIMM)) in order to reduce drug-related readmissions and outpatient visits. | Patients admitted to and discharged from hospital | Cost effectiveness model | Medication reconciliation by pharmacists at admission, during hospital stay, and after discharge including communication with the patient, and the primary and/or community care provider at discharge. | HCP | ADEs, medication errors and discrepancies;  Overall healthcare use;  Costs |
| Gilbert 2012  [62]  Australia  N=77 | HOS->PCP Discharge | Patient outcome | Medication safety at discharge | To compare the number and clinical significance of medication errors and omission in discharge medicines information, and the timeliness of delivery of this information to community-based health practitioners, between the existing Hospital Discharge Summary (HDS) and a pharmacist prepared Medicines Information Transfer Fax (MITF). | Patients at high risk of medication errors | Observational study: prospective design |  | HCP | ADEs, medication errors and discrepancies |
| Glintborg 2007  [63]  Denmark N=200 | HOS->PCP Discharge | Patient outcome | Medication safety at discharge | To estimate whether hospitals succeed in making accurate medication lists congruent with patients’ actual medication use. Subsequently, the authors evaluated where errors were introduced and the possible implications of incongruent medication lists. | Patients scheduled for discharge (medical and surgical ward) | Observational study: prospective design |  | HCP & patient | ADEs, medication errors and discrepancies |
| Graumlich 2009a  [64]  USA  Physicians N=70, Patients: N=631 | HOS->PCP Discharge | HCP & patient outcome | Transitional communication | To measure patient and physician perceptions after discharge with computerized physician order entry (CPOE) software. | Hospital physicians and their patients discharged home | Intervention study: RCT | Discharge software facilitating communication at the time of hospital discharge to patients, retail pharmacists, and community physicians. | HCP & patient | HCP satisfaction and opinions; Interprofessional collaboration and communication; Patient care experiences;  Patient satisfaction; B-prepared (Graumlich et al.); Physician prepared (Graumlich et al.); Questionnaires: related concepts and non-validated questionnaires |
| Graumlich 2009b  [65]  USA Physicians N=69, Patients: N=631 | HOS->PCP Discharge | Patient outcome | Transitional communication | To measure effects of a discharge software application of computerized physician order entry (CPOE). | Hospital physicians and their patients discharged home | Intervention study: RCT | Discharge software facilitating communication at the time of hospital discharge to patients, retail pharmacists, and community physicians. | Patient | Readmission;  ED visits;  Harm and adverse events |
| Gray 2008  [66]  UK  N=86 | HOS->PCP Discharge | HCP outcome | Medication reconciliation | To investigate the effect of sharing information about patients’ medication between the hospital and practice-based primary care pharmacists. It also sought to assess whether this led to improved implementation of treatment plans after discharge from hospital and could be integrated into the current workload of the practice-based pharmacists. | Discharged elderly patients | Intervention study: before- after design | Information sharing about patients’ medication between hospital and primary care pharmacists. | HCP | Quality and timeliness of discharge summary |
| Grimes 2008  [67]  Ireland  N=139 | HOS->PCP Discharge | Patient outcome | Medication safety at discharge | To investigate the quality and consistency of medication details generated for such transfer from an Irish teaching hospital. | Adult cardiology patients admitted to hospital | Observational study: prospective design |  | HCP | ADEs, medication errors and discrepancies |
| Grimes 2011  [68]  Ireland N=1200 | HOS->PCP Discharge | Patient outcome | Medication safety at discharge | To investigate the factors contributing to medication reconciliation on discharge, and identify the prevalence of non-reconciliation. | Adult patients discharged and prescribed at least one medicine | Observational study: retrospective design |  | HCP | ADEs, medication errors and discrepancies;  Quality and timeliness of discharge summary; Interprofessional collaboration and communication |
| Hamar 2016 [69]  USA  Intervention N=560, Control N=3340 | HOS->PCP Discharge | Patient outcome | Discharge safety | To evaluate the Care Transition Solution as means to improve quality through reduction of preventable hospital readmissions among patients with readmission-sensitive conditions subject to penalties imposed by the Affordable Care Act. | Admitted patients with readmission-sensitive conditions | Observational study: retrospective design | A nurse transition coach assessing risks at discharge, reconciliation of medications, a patient-oriented Care Transition Record, with documented discharge plan; coordination of medical providers and service agencies for post-discharge patient care; and a post-discharge telephonic follow-up | HCP | Readmission |
| Hanlon 2006  [70]  USA  N=808 | HOS->PCP Discharge | Patient outcome | Medication safety at discharge | To examine the incidence and predictors of all and preventable ADEs in frail elderly persons after hospital discharge, a highly vulnerable but rarely studied population. | Frail elderly persons discharged home. | Observational study: retrospective design |  | HCP | ADEs, medication errors and discrepancies;  Harm and adverse events |
| Hansen 2013  [71]  USA  Patient N=NR  Hospitals: Intervention N=11, Control=19 | HOS->PCP Discharge | Patient outcome | Discharge safety | To determine the effect of Project BOOST (Better Outcomes for Older adults through Safe Transitions) on rehospitalisation rates and length of stay. | All patients admitted to a clinical acute-care unit of 11 hospitals | Intervention study: non-randomized controlled trial | Hospitals receive expert mentoring and peer support to aid in improving the care of patients as they transition from hospital to home including map ping current processes and creating and implement action plans for organizational change. | HCP | Readmission;  Length of hospital stay;  Implementation (number/ quality) |
| Hastings 2011  [72]  USA  N=92 | HOS->PCP Discharge | Patient outcome | Discharge safety from ED | To describe older patients’ understanding of ED discharge information and to explore the relationship between understanding of ED discharge information and adverse outcomes. | Patients 65 years and older discharged home from the ED | Observational study: prospective design |  | Patient | All-cause mortality; Readmission;  ED visits;  Harm and adverse events;  Physical and psychosocial outcomes;  Patient knowledge |
| Hawes 2013  [73]  USA  N=61 | HOS->PCP Discharge | Patient outcome | Medication safety at discharge | To evaluate the impact of a transitional care clinic visit conducted by a pharmacist compared with usual care on the resolution of medication discrepancies and incidence of rehospitalisation’s and ED visits. | Admitted patients with risk factors for rehospitalisation who also received primary care | Intervention study: RCT | A care transitions clinic visit with a clinical pharmacist approximately 72 hours post-discharge, and prior to the post hospitalization PCP visit, including a complete medication history, identifying and resolving medication discrepancies, creating a current medication list for both the medical record and the patient, and counselling on appropriate medication use. | HCP | ADEs, medication errors and discrepancies; Readmission;  ED visits |
| Hazen 2015  [74]  the Netherlands  Sample size calculation: 2850 per arm (three arms) | PCP<->HOS Overall continuity | Patient outcome | Medication safety | To evaluate the effect of integration of a non-dispensing pharmacist in general practice on the safety of pharmacotherapy in the Netherlands. | Elderly patients with multimorbidity and polypharmacy | Intervention study: non-randomized controlled trial | An assessment by a pharmacist of the patient’s drug-related needs, a care plan to meet the specific needs of the patient, and a follow-up evaluation to determine the impact of the decisions made and actions taken. | HCP | ADEs, medication errors and discrepancies;  Drug-related hospital (re) admission;  Costs |
| Hellstrom 2012  [75]  Sweden  N=670 | PCP->HOS Admission | Patient outcome | Medication safety at admittance | To describe the frequency, type and predictors of errors in medication history, and to evaluate the extent to which standard care corrects these errors. | All patients admitted to two internal medicine wards | Observational study: prospective design | Clinical pharmacists conducted medication reconciliations | HCP | ADEs, medication errors and discrepancies |
| Heyworth 2013  [76]  USA  N=60 | HOS->PCP Discharge | Patient outcome | Medication reconciliation | To pilot test a medication reconciliation tool delivered via a web portal to improve medication safety among patients recently discharged from hospital. | Hospitalized (adult) veteran patients | Observational study: prospective design | Medication reconciliation by a multidisciplinary team, including physicians, a pharmacist and a nurse informaticist using interactive software so that patients could perform medication reconciliation independently at home | HCP | ADEs, medication errors and discrepancies;  Patient care experiences;  Harm and adverse events |
| Hohmann 2014  [77]  Germany Patient N= 310  PCP N= 111 | HOS->PCP Discharge | HCP & patient outcome | Medication safety at discharge | To develop a structured medication report as part of the discharge letter, to evaluate the impact of the medication report in ischaemic stroke patients and to identify the most important issues in the transitional care process of stroke patients. | Adult patients with either a diagnosis of transient ischaemic attack or ischaemic stroke taking two or more drugs during hospital stay and at discharge | Intervention study: before- after design | Medication reconciliation during hospital stay and a structured medication report created by a pharmacist, as part of the discharge letter. | HCP | Quality and timeliness of discharge summary;  HCP satisfaction and opinions;  Patient adherence |
| Holland 2011  [78]  USA  N=371 | HOS->PCP Discharge | HCP & patient outcome | Discharge safety and experience | To report how we evaluated a change in DP practice from the perspective of recently discharged patients. | Adult patients recently discharged from a medical or surgical nursing unit | Intervention study: non-randomized controlled trial | Discharge planning decision support tool. | Patient | Patient care experiences;  Patient knowledge;  Care transition measure (CTM) (Coleman et al.) |
| Horwitz 2013  [79]  USA  N=377 | HOS->PCP Discharge | HCP outcome | Quality of discharge letter | To conduct a comprehensive quality assessment of discharge summaries. | Patients discharged home after hospitalization for acute coronary syndrome, heart failure, or pneumonia. | Observational study: retrospective design |  | HCP | Quality and timeliness of discharge summary; Questionnaires: related concepts and non-validated questionnaires |
| Hu 2012  [80]  USA  N=82 | HOS->PCP Discharge | Patient outcome | Medication safety at discharge | To examine, in older Chinese Americans, the prevalence of both potential inappropriate medications (PIMs) and medication discrepancies (MDs); the relationship between PIMs and MDs; and the patient and hospitalization characteristics associated with them during care transitions from hospital discharge to home care. | Elderly Chinese Americans receiving home after hospital discharge and taking at least one prescribed medication at hospital discharge. | Observational study: prospective design |  | HCP & patient | ADEs, medication errors and discrepancies; Questionnaires: related concepts and non-validated questionnaires |
| Huynh 2016 [81]  UK  N=142 | HOS->PCP Discharge | Patient outcome | Medication reconciliation | To investigate the incidence, nature and potential clinical severity of medication discrepancies at the point of hospital discharge in a paediatric setting. | Paediatric patients discharged from hospital | Observational study: prospective design |  | HCP | ADEs, medication errors and discrepancies |
| Jack 2009  [82]  USA  N=749 | HOS->PCP Discharge | Patient outcome | Discharge safety | To test the effects of an intervention designed to minimize hospital utilization after discharge. | English-speaking hospitalized adults | Intervention study: RCT | A nurse discharge advocate to arrange follow-up appointments, confirm medication reconciliation, and conduct patient education and a medication review post-discharge by a clinical pharmacist. | HCP & patient | Readmission;  Overall healthcare use;  PCP follow up;  ED visits;  Physical and psychosocial outcomes;  Patient knowledge |
| Julian 2007  [83]  UK  N=193 | PCP->HOS Referral | Patient outcome | Continuity of care and referral safety | To examine the outcomes of an integrated model that lends weight to GP-led evidence based care. | Women with menstrual disorders | Intervention study: non-randomized controlled trial | Facilitate patients’ access to diagnostic and therapeutic facilities within secondary care, by shared evidence based guidelines for the management of patients in both primary and secondary care, which determined timing for investigations and surgical treatment. | HCP & patient | Overall healthcare use;  Patient care experiences;  Physical and psychosocial outcomes |
| Kanaan 2013  [84]  USA  N=1000 | HOS->PCP Discharge | Patient outcome | Medication safety at discharge | To characterize ADEs occurring within the high-risk 45-day period after hospitalization in older adults. | Elderly hospitalized patients discharged home. | Observational study: retrospective design |  | HCP | ADEs, medication errors and discrepancies;  Harm and adverse events |
| Kantelhardt 2016 [85]  Germany  Control N=149, Intervention N=163 | PCP<->HOS Admission and discharge | Patient outcome | Medication reconciliation | To investigate medication-related errors in spinal instrumentation patients and apply current medication reconciliation strategies. | All patients 18 years and older, who underwent spinal instrumentation | Intervention study: before- after design | A medication list at admission and which was stopped before surgery, and discharge, to remind the discharging physician of home medication. A special focus was on anticoagulants | HCP | ADEs, medication errors and discrepancies;  Patient adherence |
| Karapinar-Carkit 2010  [86]  The Netherlands  Sample size calculation N= 360 patients per group | HOS->PCP Discharge | HCP, patient & organisational outcome | Discharge safety | To determine the effect of the COACH program (Continuity Of Appropriate pharmacotherapy, patient Counselling and information transfer in Healthcare) on readmission rates in patients discharged from the internal medicine department. | Patients admitted to an internal medicine ward with at least one prescribed drug intended for chronic use | Intervention study: before- after design | Medication reconciliation, patient counselling and communication between the hospital and primary care healthcare providers. | HCP & patient | ADEs, medication errors and discrepancies; Readmission;  Costs;  HCP satisfaction and opinions;  Patient adherence; Patient satisfaction; Physical and psychosocial outcomes |
| Keers 2015 [87]  UK  N=274 | HOS->PCP Discharge | Patient outcome | Medication safety at discharge | To investigate the quality and safety of discharge prescriptions written at mental health hospitals. | Patients admitted to acute adult and later  life inpatient wards | Observational study: prospective design |  | HCP | ADEs, medication errors and discrepancies;  Quality and timeliness of discharge summary |
| Kennedy 2014 USA  [88]  Sites N=7 Pharmacists N=8 | PCP<->HOS Overall continuity | HCP & organisational outcome | Medication reconciliation | To determine the impact of integrating pharmacists into patient-centred medical homes, with a focus on population management. | Primary care patients | Observational study: prospective design | A pharmacist in the primary care practice providing direct patient care, educating prescribers, and using an approach to identify population-based opportunities to improve prescribing. | HCP | ADEs, medication errors and discrepancies;  Costs |
| Kind 2012  [89]  USA  N= 708 | HOS->PCP Discharge | Patient outcome | Discharge safety | To provide a detailed outline of the program’s clinical protocols and feasibility and an assessment of its impact on thirty-day rehospitalisation. | Elderly, community-dwelling veterans at high risk of poor posthospital outcomes, hospitalized on medical or surgical wards and discharged home | Intervention study: before- after design | A registered nurse case manager to coordinate the veteran’s transitional care through active participation in inpatient multidisciplinary discharge rounds, a single brief protocol-driven inpatient encounter, and one to four protocol-driven post hospital telephone calls with the veteran and, if available, the veteran’s hcp. | Patient | ADEs, medication errors and discrepancies; Readmission |
| King 2008  [90]  USA  N=46 | HOS->PCP Discharge | Patient & organisational outcome | Discharge safety | To determine if implementation of a nurse-driven discharge planning protocol for patients undergoing cardiac implant would result in improved organizational efficiencies, higher medication reconciliation rates, and higher patient satisfaction scores. | Patients scheduled for a cardiac implant procedure | Intervention study: RCT | A physical assessment, review of the post procedure chest radiograph, and examination of the cardiac implant device function by an intervention nurse. The intervention nurse also provided patient education, discharge instructions, and conducted medication reconciliation and conducted a scripted follow-up phone call to answer questions and monitor for post procedure complications. | HCP & patient | ADEs, medication errors and discrepancies; Length of hospital stay;  Costs;  Patient satisfaction; Physical and psychosocial outcomes; Questionnaires: related concepts and non-validated questionnaires |
| Knez 2011  [91]  Slovenia N=101 | PCP<->HOS Admission and discharge | Patient outcome | Medication reconciliation | To evaluate the need for medication reconciliation. | Adult patients with at least one drug discharged from hospital | Observational study: prospective design |  | HCP | ADEs, medication errors and discrepancies; Questionnaires: related concepts and non-validated questionnaires |
| Koehler 2009  [92]  USA  N=41 | HOS->PCP Discharge | HCP & patient outcome | Discharge safety | To assess the impact of a supplemental care bundle targeting high-risk elderly inpatients implemented by hospital-based staff compared to usual care on a composite outcome of hospital readmission and/or ED visitation at 30 and 60 days following discharge. | Elderly medical inpatients predisposed to unplanned readmission or post-discharge ED visitation | Intervention study: RCT | A multicomponent intervention following hospital discharge by a care coordinator and clinical pharmacist including: education, identifying discharge barriers, medication reconciliation at admittance and discharge, personal health record, fax with information to PCP and follow up phone call after discharge. | HCP | ADEs, medication errors and discrepancies; Readmission;  Length of hospital stay;  ED visits;  Physical and psychosocial outcomes |
| Lai 2008  [93]  USA  N=79 | HOS->PCP Discharge | Initiatives & HCP outcome | Discharge safety | To determine whether a discharge curriculum would improve students’ attitudes and self-assessed skills in interdisciplinary collaboration and transitional care for chronically ill patients. | Third-year medical students and fourth-year pharmacy students | Intervention study: before- after design | An interdisciplinary curriculum on transitional care including a post-discharge visit to a patient | HCP | HCP: satisfaction and opinions;  confidence and skills;  attitudes |
| Lalonde 2008  [94]  Canada  N=83 | HOS->PCP Discharge | HCP & patient outcome | Medication reconciliation | To assess the effect of a medication discharge plan (MDP) on the rate of medication discrepancies between hospital and outpatient settings. | Adult patients, discharged from a geriatric, family medicine, or psychiatric ward with at least two pharmacotherapeutic changes | Intervention study: RCT | A medication discharge plan containing medication at admittance, mutations and medication at discharge. | HCP & patient | ADEs, medication errors and discrepancies |
| Lassere 2015  [95]  USA  Sample size calculation: N=792 | PCP<->HOS Overall continuity | Patient outcome | Continuity of care | To assess the acceptability and long-term clinical outcomes of an electronic and paper patient-held portable health files (PHF). | Community-dwelling patients, 60 years or older, who have two or more chronic medical conditions that require prescription medication and regular care by at least three medical practitioners (general and specialist care) | Intervention study: RCT | A patient-held portable health file | HCP & patient | ADEs, medication errors and discrepancies;  All-cause mortality; Overall healthcare use;  Admission and inpatient care use;  Unscheduled hospital or PCP visit;  Redundant diagnostic testing;  Patient adherence; Harm and adverse events;  Physical and psychosocial outcomes;  Feasibility intervention/ user friendliness |
| Lee 2009  [96]  USA  N=241 | HOS->PCP Discharge | Patient outcome | Medication safety; analgesics | To describe the epidemiology of controlled substance prescription errors by physicians-in-training for children being discharged from the hospital. | Discharged paediatric patients | Observational study: prospective design |  | HCP | ADEs, medication errors and discrepancies;  Harm and adverse events;  Questionnaires: related concepts and non-validated questionnaires |
| Lesselroth 2009  [97]  USA  N=111 | PCP->HOS Outpatient clinic visit | HCP & patient outcome | Medication reconciliation | To describes how we developed and implemented a process for patients in the waiting room to use kiosk technology in providing their own medication histories. | Patients of a chemotherapy administration unit | Observational study: prospective design | Automated patient history intake device using reconciliation software program accessed by the patient using a computer terminal kiosk located in the clinic lobby. | HCP & patient | ADEs, medication errors and discrepancies; Productivity;  Patient- HCP communication |
| Lindpaintner  2013  [98]  Switzerland  N=60 | HOS->PCP Discharge | HCP & patient outcome | Discharge safety | To improve patient outcomes following hospital discharge and on reducing barriers to continuity and provider efficiency. | Patients at high risk for adverse events | Intervention study: RCT | An individualized discharge plan was formulated by a nurse case manager in collaboration with the physician team (multicomponent: pt education on self-management, fax with information to PCP, evaluating telephone call within 24 hours of discharge, availability for questions, home visit). | HCP & patient | ADEs, medication errors and discrepancies; All-cause mortality; Readmission;  PCP follow up;  ED visits;  Quality and timeliness of discharge summary; Costs;  HCP satisfaction and opinions;  Patient satisfaction; Physical and psychosocial outcomes;  Caregiver burden |
| Lindquist 2013  [99]  USA  N=114 | HOS->PCP Discharge | Patient outcome | Discharge safety | To determine whether PCP contact with patients at hospital discharge impacts the frequency of medication discrepancies at 24 hours post-discharge. | Community dwelling adults, admitted to acute medicine services on at least 5 medications | Intervention study: before- after design | When the patient was nearing discharge from the hospital, hospitalists were asked to phone the PCP to discuss the patient’s discharge plan and facilitate clinical handoffs to the outpatient setting. Primary care providers were asked to speak with treating hospitalists and contact patients within 24 hours of hospital discharge to discuss any hospital medication changes. | Patient | ADEs, medication errors and discrepancies;  PCP follow up |
| Lingaratnam  2012  [100]  Australia  Control N=223, intervention N= 348 | HOS->PCP Discharge | Initiatives, HCP & patient outcomes | Medication reconciliation | To design and test the effectiveness of a consumer–healthcare professional partnership model towards effective medication reconciliation. | Patients admitted to a surgical ward (intervention), and an oncology ward (control) | Intervention study: non-randomized controlled trial | Medication reconciliation including education of the patient, a medication list from the pharmacist at discharge, telephone call after patient discharge. | HCP & patient | ADEs, medication errors and discrepancies;  Patient satisfaction; Harm and adverse events;  Patient knowledge; Patient empowerment |
| Linsky 2012  [101]  USA  N=106 | PCP<->HOS All transitions | Patient outcome | Medication reconciliation | To determine the prevalence of discrepancies within a national healthcare system with electronic health records –pharmacy linkage to characterise the medications involved and to identify factors associated with discrepancies. | Patients from ambulatory care clinics (including Primary Care, Rheumatology and Heart Failure clinics) | Observational study: retrospective design |  | HCP | ADEs, medication errors and discrepancies; Questionnaires: related concepts and non-validated questionnaires |
| Manning 2007  [102]  USA  N=138 | HOS->PCP Discharge | HCP & patient outcome | Medication reconciliation | To establish of in terms of patient satisfaction, understanding and safety, 3D is better than the Medication Discharge Worksheet (MDW), (3D=custom software enabling semi-automatic 3D form generation) | Adult patients, with more than three discharge medications, and returning home | Intervention study: RCT | Custom software creating an automatic medication form at discharge | Patient | ADEs, medication errors and discrepancies;  Harm and adverse events;  Patient knowledge |
| Martinussen  2013  [103]  Norway N=1298 | PCP->HOS Referral | Climate & HCP outcome | Referral quality | To investigate how hospital physicians view the general quality of patient referrals received from GPs, and the extent to which insufficient information in referrals and inappropriate referrals is considered a problem for cooperation with GPs. | Publicly employed hospital physicians | Observational study: cross-sectional design |  | HCP | HCP satisfaction and opinions; Interprofessional collaboration and communication |
| Maslove 2009  [104]  Canada  PCP N=119,  Patients N=209, Housestaff N=12 | HOS->PCP Discharge | HCP outcome | Discharge safety | To assess PCP satisfaction with an electronic discharge summary (EDS) program as compared to conventional dictated discharge summaries. | Patients discharged home from General Internal Medicine department | Intervention study: RCT | An electronic discharge summary to improve the quality of a discharge summary including preadmission information, hospital course, and discharge and follow-up plans. | HCP | Readmission;  ED visits;  Diagnostic test follow up;  Quality and timeliness of discharge summary;  HCP satisfaction and opinions;  Patient adherence; Patient care experience;  Patient knowledge;  Care transition measure (CTM) (Coleman et al.) |
| Mazor 2016 [105]  USA  N=297 | PCP->HOS referral | HCP outcome | Timely referral | To examine the extent to which primary care practitioners' perceptions of event-level, physician-level and organisation-level factors influence a physician's intent to disclose a medical error in stressful situations. | PCPs from an integrated healthcare delivery systems | Observational study: cross-sectional design |  | HCP | Patient-HCP communication; Referral: timeliness and inappropriateness |
| McCormick 2015 [106]  USA  N=220 | HOS->PCP Discharge | Patient outcome | Home care after discharge (tracheostomy) | To report patient/family experiences and outcomes after tracheostomy. | Parents of children with a tracheostomy tube but also adolescents and adults with a tracheostomy tube themselves | Observational study: cross-sectional design |  | Patient | Overall healthcare use;  Patient care experiences;  Harm and adverse events;  Patient knowledge |
| McMillan 2006  [107]  New zealand N=200 | HOS->PCP Discharge | Patient outcome | Medication safety at discharge | To audit discharge summaries at Auckland Hospital to determine the frequency and nature of any medication errors. We have attempted to identify factors that contribute to these errors. | Patients discharged from hospital | Observational study: retrospective design |  | HCP | ADEs, medication errors and discrepancies;  Quality and timeliness of discharge summary |
| McMillan 2013  [108]  USA  N=120 | PCP<->HOS Admission and discharge | Patient outcome | Medication reconciliation | To evaluate communication and medication discrepancies on admission and discharge between PCPs without admitting privileges and hospitalist physicians. | All patients admitted from primary care practices | Observational study: retrospective design |  | HCP | ADEs, medication errors and discrepancies; Referral: timeliness and inappropriateness |
| Meran 2010  [109]  UK  N=88 | HOS->PCP Discharge from oupatient clinic | HCP & patient outcome | Safety of substitution/ discharge to PCP | To determine if patients discharged from nephrology clinic were appropriately discharged and adequately monitored in primary care. In addition, we prospectively follow-up the discharged patients’ renal parameters and determine whether they were appropriately re-referred to nephrology services. | All patients discharged from a renal outpatient clinic | Observational study: prospective design | A renal patient care pathway. | HCP | All-cause mortality; Re-referrals and referral rate;  Specific disease-related outcomes |
| Mesteig 2010  [110]  Norway  N=131 | HOS->PCP Discharge | Patient outcome | Discharge safety | To describe unwanted incidents registered by the ambulatory team among patients discharged from a geriatric evaluation and management unit (GEMU) by character, frequency and stage in the transitional process. Only unwanted incidents with a severity making contact with the primary health care necessary were registered. | All patients planned to be discharged from a geriatric evaluation and management unit to their homes | Observational study: prospective design | An ambulatory team as part of the geriatric evaluation and management unit to improve the transitional process from hospital to the patients' homes. | HCP & patient | ADEs, medication errors and discrepancies; Quality and timeliness of discharge summary; Harm and adverse events |
| Meyers 2014  [111]  USA  Sample size calculation: N=3000 | HOS->PCP Discharge | Patient outcome | Discharge safety | To enhance understanding of how health literacy and other patient factors affect the quality of care transitions and outcomes after hospitalization. | Adult patients hospitalized with acute coronary syndromes or acute decompensated heart failure | Observational study: prospective design |  | HCP & patient | All-cause mortality; Readmission;  Overall healthcare use;  ED visits;  Physical and psychosocial outcomes |
| Mishra 2009  [112]  Nepal  N=130 | HOS->PCP Discharge | HCP outcome | Quality of discharge letter | The study aims to assess the adequacy of medical records in Bir Hospital, a central hospital. | Patients admitted to a Liver Unit of a central tertiary care hospital | Observational study: cross-sectional design |  | HCP | Quality and timeliness of discharge summary |
| Misky 2010  [113]  USA  N=65 | HOS->PCP Discharge | Patient outcome | Discharge safety | To evaluate characteristics and outcomes of discharged patients lacking timely PCP follow-up, defined as within 4 weeks of discharge. | Adult patients admitted to internal medicine | Observational study: prospective design |  | Patient | Readmission; PCP follow up;  Length of hospital stay;  ED visits |
| Moore 2003  [114]  USA  N=86 | HOS->PCP Discharge | Patient outcome | Discharge safety and medication safety at discharge | To determine the prevalence of medical errors related to the discontinuity of care from an inpatient to an outpatient setting, and to determine if there is an association between these medical errors and adverse outcomes. | Patients discharges who had subsequent visit with an outpatient primary care provider within 2 months of discharge | Observational study: retrospective design |  | HCP | ADEs, medication errors and discrepancies; Readmission;  Diagnostic test follow up |
| Moore 2011  [115]  UK  N=377 | PCP<->HOS Admission and discharge | Initiatives & patient outcome | Medication reconciliation | To evaluate the use of a shared electronic primary health care record (EHR) to assist with medicines reconciliation in the hospital from admission to discharge. | Patients admitted to the elderly care admissions ward | Observational study: prospective design | Medication reconciliation using a shared electronic primary health record | HCP | ADEs, medication errors and discrepancies;  Harm and adverse events |
| Moyer 2014  [116]  USA  N=229 | HOS->PCP Discharge | HCP & patient outcome | Discharge safety and experience | To test whether a multifaceted intervention that included a health coach to assist families and an enhanced personal health record to improve the quality of information available to parents and community professionals would decrease adverse events and improve family assessment of the transition. | The families of all newborn infants admitted for at least 2 weeks to a newborn center, also registered with a primary care provider | Intervention study: non-randomized controlled trial | A transition coach and a personal health record. | HCP & patient | All-cause mortality; Readmission;  Patient adherence; Patient care experiences;  Patient knowledge; Caregiver burden; Caregiver satisfaction;  Care transition measure (CTM) (Coleman et al.) |
| Murphy 2009  [117]  USA  N=760 | PCP<->HOS Admission and discharge | Patient outcome | Medication reconciliation | To implement a comprehensive medication reconciliation program to reduce errors in admission and discharge medication orders at an academic medical centre is described. | Every inpatient admitted to hospital | Observational study: prospective design | Medication reconciliation by a pharmacist at admission and discharge: a discharge medication report was developed for patients to take home. | HCP | ADEs, medication errors and discrepancies |
| Musgrave 2013  [118]  USA  N=192 | HOS->PCP Discharge | Patient outcome | Medication reconciliation at discharge | To critically evaluate the discharge process for solid-organ transplant recipients in order to determine if a transplant pharmacists’ formalized involvement with the discharge medication reconciliation process would improve medication safety by decreasing the rate and severity of medication errors. | Patients discharged from an abdominal transplant surgery service | Intervention study: non-randomized controlled trial (historical control) | Medication reconciliation verified by a transplant pharmacist. | HCP | ADEs, medication errors and discrepancies |
| Nana 2012  [119]  USA  N=102 | PCP<->HOS Admission and discharge | Patient, initiatives & HCP outcome | Medication reconciliation | To describe the initiation of a medication reconciliation program and other pharmacy services in the ED of a community and to address errors and inconsistencies in ED admission and discharge medication reconciliations. | ED patients | Observational study: prospective design | An ED pharmacist performing admission medication, entering ED pharmacy orders, facilitating medication delivery, and providing drug information | HCP | ADEs, medication errors and discrepancies;  HCP satisfaction and opinions |
| Naylor 1990  [120]  USA  N=40 | HOS->PCP Discharge | Patients & organisational outcome | Discharge safety | To examine the effects of a comprehensive discharge planning protocol implemented by a gerontological nurse specialist as compared to the hospital's general discharge planning procedure. | Community-dwelling hospitalized elders, admitted to a medical or surgical unit | Intervention study: RCT | A discharge planning protocol including: assessment of unique needs of elderly patients which were communicated to their primary nurses and documented, validation of patients and healthcare professionals understanding, two visits during hospitalization, possibility of contact and two telephone-contacts post-discharge. | HCP | Readmission;  Length of hospital stay; Specific disease-related outcomes; Costs |
| Naylor 1994  [121]  USA  Patients N=276, HCP N=125 | HOS->PCP Discharge | Patient & organisational outcome | Discharge safety | To study the effects of a comprehensive discharge planning protocol, designed specifically for the elderly and implemented by nurse specialists, on patient and HCP outcomes and cost of care. | Hospitalised patients, 70 years and older | Intervention study: RCT | A comprehensive discharge planning and home follow-up protocol designed specifically for elders at high risk for poor post-discharge outcomes | HCP | Readmission;  Length of hospital stay;  Costs |
| Naylor 1999a  [122]  USA  N=363 | HOS->PCP Discharge | Patient & organisational outcome | Discharge safety | To examine the effectiveness of an advances practice nurses-centred comprehensive discharge planning and home follow-up protocol for elders hospitalized. | Elderly patients at risk for readmissions | Intervention study: RCT | Comprehensive discharge planning and home follow-up protocol designed for elders at risk for poor outcomes after discharge and implemented by advanced practice nurses. | HCP & patient | Readmission; ED visits; Costs;  Patient satisfaction; Physical and psychosocial outcomes |
| Naylor 1999b  [123]  USA  N=202 | HOS->PCP Discharge | Patient outcome | Discharge safety | To examine the effectiveness of a discharge planning and home follow-up management program for elders hospitalized with common medical and surgical cardiac conditions. | Community-dwelling, elderly patients, admitted with heart failure, angina, myocardial infarction, coronary artery bypass graft surgery, or cardiac valve replacement | Intervention study: RCT | A comprehensive discharge planning and home follow-up protocol designed specifically for elders at high risk for poor post-discharge outcomes. | HCP | Readmission;  Physical and psychosocial outcomes |
| Naylor 2004  [124]  USA  N=239 | HOS->PCP Discharge | Patient &organisational outcome | Discharge safety | To examine the effectiveness of a transitional care intervention delivered by advanced practice nurses to elders hospitalized with heart failure. | Elderly patients hospitalized with heart failure | Intervention study: RCT | A 3-month discharge planning and home follow-up protocol by advanced practice nurses. | HCP & patient | All-cause mortality; Readmission;  Overall healthcare use;  Costs;  Patient satisfaction; Physical and psychosocial outcomes |
| Naylor 2011  [125]  USA  N=172 | HOS->PCP Discharge | Patients, HCP & organisational outcome | Discharge safety | To evaluate the impact of translating into a large US health plan, the Transitional Care Model (TCM), an evidence-based approach to address the needs of chronically ill older adults throughout acute episodes of illness. | Chronically ill older adults throughout acute episodes of illness | Intervention study: before- after design | An advanced practice nurse comprehensive in-hospital planning and home follow-up. | HCP & patient | Overall healthcare use;  Costs;  HCP satisfaction and opinions;  Patient satisfaction; Physical and psychosocial outcomes |
| Nuñez 2006  [126]  Spain  N=500 | HOS->PCP Discharge | Patient outcome | Discharge safety from ED | The causes of unscheduled returns to the ED within 72 hours of discharge are unclear. A study was undertaken to identify factors associated with this quality care indicator. | Patients over 14 years, presenting to the ED | Observational study: retrospective design |  | HCP | ADEs, medication errors and discrepancies; Readmission;  PCP follow up;  ED visits |
| Nuno-Solinis 2013  [127]  Spain  N=146 | PCP<->HOS Overall continuity | Climate & HCP outcome | Interprofessional cooperation | To assess the change in the degree of interprofessional collaboration across two different levels of care, before and after the establishment of the Bidasoa Integrated Healthcare Organisation, based on the perceptions of two groups of health professionals (doctors and nurses). | Clinical professionals at different levels of care | Intervention study: before- after design | Organisational integration of the clinical processes between different care levels. | HCP | Interprofessional collaboration and communication; Collaboration questionnaire (Nuno Solinis et al.) |
| Okoniewska  2012  [128]  Canada Sample size calculation: N= 1400 | HOS->PCP Discharge | Patient & organisational outcome | Discharge safety/ communication at discharge | To examine whether a transfer-of-care communication tool is efficacious and cost-effective for reducing hospital readmission, adverse events and ADEs as well as reducing death. | Adult, community-dwelling patients admitted to hospital | Intervention study: RCT | Patient’s discharge summaries generated by a computer-enabled communication tool providing a standardized template for communicating all relevant clinical information on patients who are leaving hospital, is immediately available at time of leaving and is accessible through the web by community-based providers (family physicians, specialists, home care nurses, and community pharmacists). | HCP | ADEs, medication errors and discrepancies;  All-cause mortality; Readmission;  Costs;  Harm and adverse events;  Physical and psychosocial outcomes |
| Ong 2016 [129]  USA  Intervention N=715, Control N=722 | HOS->PCP Discharge | Patient outcome | Discharge safety | To evaluate the effectiveness of a care transition intervention using remote patient monitoring in reducing 180-day all-cause readmissions among a broad population of older adults hospitalized with heart failure. | Older adults hospitalized with heart failure | Intervention study: RCT | The intervention combined health coaching telephone calls and telemonitoring. Telemonitoring used electronic equipment that collected daily information about blood pressure, heart rate, symptoms, and weight. Centralized registered nurses  conducted telemonitoring reviews, protocolized actions, and telephone calls. | HCP & patient | All-cause mortality; Readmission;  Physical and psychosocial outcomes |
| Ouchida 2009  [130]  USA  N=103 | HOS->PCP Discharge | Climate & HCP outcome | Transitional care | To assess the effect of a new curriculum on transitional care on participants’ knowledge, attitudes, and self-reported behaviours in these domains. | Third-year medical students completing their required internal medicine rotations | Intervention study: before- after design | A new curriculum emphasizing transitional care, functional assessment, interdisciplinary team, community resources, and reimbursement. The curriculum consists of two 90-minute sessions that incorporate interdisciplinary lectures, an educational digital video disc (DVD), small-group discussion, and a team-based learning exercise. | HCP | ADEs, medication errors and discrepancies;  All-cause mortality; Readmission;  Costs |
| Parrish 2009  [131]  USA  N=791 | HOS->PCP Discharge | Initiatives, HCP & patient outcomes | Discharge safety and self-management | To identify factors that promote sustainability of the intervention by assessing features of each site's implementation and the site's likelihood of continuing the program; soliciting feedback from the sites; and analysing site and patient characteristic data and data from the Care Transitions Intervention measurement instruments (the 3-ltem Care Transition Measure [CTM-3) and the Patient Activation Assessment [PAA) tool). | Elderly discharged patients | Observational study: prospective design | The Care Transitions Intervention: a self-management program designed to provide patients discharged from the acute care setting with skills, tools, and the support of a transition coach to ensure that their health and self-management needs are met. | HCP & patient | ADEs, medication errors and discrepancies;  All-cause mortality; Readmission;  Costs;  Physical and psychosocial outcomes; Implementation (number/ quality);  Care transition measure (CTM) (Coleman et al.); Medication discrepancy tool (Smith et al); Questionnaires: related concepts and non-validated questionnaires |
| Paulino 2004  [132]  Europe: Austria, Denmark, Germany, The Netherlands, Portugal and Spain.  N=435 | HOS->PCP Discharge | Patient outcomes | Medication discharge safety | To examine the nature and frequency of drug related problems in community pharmacies among patients discharged from hospitals in several countries, and to examine several variables related to these drug related problems. | Patients discharged with a medication prescription | Observational study: prospective design | A systematic medication review by community pharmacists in discharged patients. | HCP & patient | ADEs, medication errors and discrepancies;  All-cause mortality; Readmission;  Costs |
| Phatak 2016 [133]  USA  Intervention N=137, Control N=141 | HOS->PCP Discharge | Patient outcomes | Discharge safety | To evaluate the impact of intensive pharmacist involvement during the acute care admission as well as for a 30-day time period post-discharge on both ADEs and readmissions. | Patients admitted to an internal medicine unit on high-risk medications or with greater than three prescription medications upon discharge home | Intervention study: RCT | Medication reconciliation at admission, a patient-specific pharmaceutical care plan, discharge counselling, and post-discharge  phone calls. | HCP & patient | ADEs, medication errors and discrepancies; Readmission;  Drug-related hospital (re) admission;  ED visit;  Patient satisfaction; Harm and adverse events;  Patient knowledge; Questionnaires: related concepts and non-validated questionnaires |
| Pourrat 2014  [134]  France  Sample size calculation: N=1176 | HOS->PCP Discharge | Patient outcome | Medication discharge safety | To assess the impact of a complex intervention comprising of medication reconciliation performed at discharge by a hospital pharmacist (HP) with communication between the HP and community pharmacist on drug related problems during the seven days following discharge. | Adult patients discharged home, and visiting the same community pharmacist for at least three months. | Intervention study: RCT | The hospital pharmacist will perform a medications reconciliation that will be communicated to the patient and their community pharmacist | HCP & patient | ADEs, medication errors and discrepancies;  All-cause mortality; Readmission;  Costs |
| Redmond 2016 [135]  Ireland  N=949 | PCP<->HOS All transitions of care | Climate s & HCP outcome | Medication reconciliation and communication | To survey GPs and community pharmacists in Ireland regarding current practices of medication management, specifically medication reconciliation, communication between health care providers and medication errors as patients transition in care. | GPs and community pharmacists in Ireland | Observational study: cross-sectional design |  | HCP | Patient safety culture;  HCP satisfaction and opinions; Interprofessional collaboration and communication;  HCP attitudes |
| Reese 2003  [136]  USA  N=15 | HOS->PCP Discharge | Patient outcome | Discharge safety and medication reconciliation at discharge | To examine feasibility of implementing a hospitalist home visit programme. | Elderly, poor, and minority patients discharged home | Observational study: prospective design | Home visit after discharge by a hospital physician including a focused history and physical, a review of medications, and a home safety evaluation. | HCP & patient | ADEs, medication errors and discrepancies;  All-cause mortality; Readmission;  Costs |
| Rice 2016 [137]  USA  N= not reported | HOS->PCP Discharge | HCP & patient outcome | Discharge safety | To describe the development, implementation, and refinement of the transitional care bundle and its effect on readmission rates, patient satisfaction, time to follow-up care after discharge, and discharge medication list errors. | Inpatients  on the internal medicine service | Observational study: prospective design | A transitional care bundle that crosses care settings and organizational functions was developed to meet needs expressed by patients. It comprises 5 elements: risk stratification, a specialized phone number for discharged patients, timely post-discharge follow-up, standardized patient discharge instructions and same-day discharge summaries, and pharmacist-supported medication reconciliation. | HCP & patient | ADEs, medication errors and discrepancies;  PCP follow up;  Visits to primary care; Questionnaires: related concepts and non-validated questionnaires |
| Riordan 2016 [138]  Ireland  N=83 | HOS->PCP Discharge | Patient outcome | Medication safety at discharge | To explore the relationship between discharge prescribing error and post-discharge medication error. | All adult in-patients discharged alive to home from any adult department | Observational study: prospective design |  | HCP | ADEs, medication errors and discrepancies |
| Roorda 2013  [139]  the Netherlands  N=502 | HOS->PCP Discharge | HCP outcome | Discharge safety | The present study explored the discharge of breast cancer patients to primary care by specialists, at the end of hospital follow-up and the experiences and views of GPs regarding transfer of follow-up to the primary care setting. | GPs of patients discharged to primary care of at the end of hospital follow-up after breast cancer | Observational study: cross-sectional design |  | HCP | ADEs, medication errors and discrepancies;  All-cause mortality; Readmission;  Costs |
| Roughead 2009  [140]  Australia  N= 109,860 | HOS->PCP Discharge | Patient outcome | Discharge safety | This study aimed to determine length of time from hospital discharge until a general practice, pharmacy or specialist visit, or care planning service. | Hospitalized veteran patients | Observational study: retrospective design |  | HCP | ADEs, medication errors and discrepancies;  All-cause mortality; Readmission;  Costs |
| Russel 2014  [141]  Australia N=106 | HOS->PCP Discharge | HCP outcome | Discharge safety/ quality of discharge letter | To assess effectiveness of an intervention on accurateness of synopsis in a discharge letter. | Discharged patients whose charts and discharge summaries were written by a general medical team | Intervention study: before- after design | A training session on discharge summaries emphasising the need (i) for a structured problem-based synopsis rather than one that is a free ranging narrative; (ii) for a brief discussion of each problem; (iii) to be clear about the principal diagnosis; and (4) in the list of secondary diagnoses, to list only the problems relevant to this admission. | HCP | ADEs, medication errors and discrepancies;  All-cause mortality; Readmission;  Costs |
| Santel 2006  [142]  USA  Incidents N=2022 | PCP<->HOS Admission and discharge | Patient outcome | Medication safety | To evaluate 2,022 medication errors that involved a reconciliation issue and were reported to an incident reporting program. | All patients | Observational study: prospective design | An incident reporting system. | HCP | ADEs, medication errors and discrepancies;  All-cause mortality; Readmission;  Costs |
| Scales 2016 [143]  Canada  N=1,014,891 | HOS->PCP Discharge | Patient outcome | Discharge safety | To evaluate rates of and risk factors for potentially unintentional medication continuation following hospitalization. | All individuals aged ≥ 66 years who were  discharged alive from an acute care hospital | Observational study: retrospective design |  | HCP | ADEs, medication errors and discrepancies |
| Schnipper 2006  [144]  USA  N= 178 | HOS->PCP Discharge | HCP & patient outcome | Medication safety at discharge | To identify drug-related problems during and after hospitalization and to determine the effect of patient counselling and follow-up by pharmacists on preventable ADEs. | Patients discharged home from the general medicine service at a large teaching hospital | Intervention study: RCT | Pharmacist counselling at discharge and a follow-up telephone call 3 to 5 days later. | HCP & patient | ADEs, medication errors and discrepancies;  All-cause mortality; Readmission;  Costs;  Harm and adverse events |
| Schnipper 2009  [145]  USA  N=322 | PCP<->HOS Admission and discharge | Patient outcome | Medication reconciliation | To measure the impact of an information technology– based medication reconciliation intervention on medication discrepancies with potential for harm. | Patients admitted to a general medicine department | Intervention study: RCT | A computerized medication reconciliation tool for admission and discharge involving physicians, nurses, and pharmacists. | HCP | ADEs, medication errors and discrepancies;  All-cause mortality; Readmission;  Costs;  Harm and adverse events |
| Scotten 2015  [146]  USA  Patient N=NR  Faculties N=8 | HOS->PCP Discharge | Climate & patient outcome | Discharge safety/ interprofessional communication | To evaluate a project, including standardized communication, and techniques to continue care from inpatient, to discharge, across the post-discharge chasm, to hand-off with the primary care provider. | Children and their families discharged after hospital admission to their primary care provider | Intervention study: before- after design | The interprofessional quality improvement initiative included: (1) evidence-based teamwork system; (2) in situ simulation; (3) creation of an IP model of care; and (4) innovations in use of telehealth technology to continue care post-discharge. | HCP | ADEs, medication errors and discrepancies;  All-cause mortality; Readmission;  Costs; Questionnaires: related concepts and non-validated questionnaires |
| Seidling 2010  [147]  Germany Patient N=9978. Hospital wards N=95 | PCP<->HOS Outpatient clinic and discharge | Initiatives, patient & HCP outcome | Medication safety | To evaluate a clinical decision support systems providing upper dose limits personalised to individual patient characteristics thus guaranteeing for specific warnings. | Outpatients and discharged patients | Intervention study: before- after design | A team of pharmacists, physicians and computer scientists built an algorithm for the definition of individual upper dose limits. We evaluated electronic prescriptions as included in discharge letters or printed onto prescription forms. | HCP | ADEs, medication errors and discrepancies;  All-cause mortality; Readmission;  Costs |
| Sharma 2011  [148]  USA  N=36 | HOS->PCP Discharge from ED | Patient outcome | Medication safety after discharge from ED | To compare the medication information that patients ultimately discharged from the ED provide to the ED staff vs. the medication information the patients provide at follow-up, and to classify and quantify the types of discrepancies between the two. | Adult patients discharged from the ED to home, reporting use of five or more medications | Observational study: retrospective design |  | HCP | ADEs, medication errors and discrepancies;  All-cause mortality; Readmission;  Costs |
| Shastri 2014  [149]  UK  N=200 | HOS->PCP Discharge | HCP outcome | Quality and timeliness of discharge letter | To audit the content and timing of discharge summaries from a psychiatric hospital as to whether it adheres to the local Trust guidelines. | Patients admitted and discharged from a psychiatric hospital | Intervention study: before-after design |  | HCP | ADEs, medication errors and discrepancies;  All-cause mortality; Readmission;  Costs |
| Shen 2013  [150]  USA  Groups of pediatric hospitalist N=7 | HOS->PCP Discharge | Initiatives & HCP outcome | Discharge safety/ timeliness of discharge letter | To achieve 90% documentation of hospitalist-PCP communication within 2 days of hospital discharge in <12 months. | All patients discharged from a paediatric hospital | Observational study: prospective design | A quality improvement project to improve the timeliness and reliability of discharge communication. After an initial face-to-face meeting, e-mail and regular conference calls were used to promote shared effort and learning. | HCP | ADEs, medication errors and discrepancies;  All-cause mortality; Readmission;  Costs |
| Shu 2011  [151]  Taiwan  N=313 | HOS->PCP Discharge | Patient outcome | Discharge safety | To investigate the impact of a quality improvement program, integrated post-discharge transitional care, in Taiwan’s hospitalist system. | Adult patients admitted from the ED and later discharged home | Intervention study: non-randomized controlled trial | A post-discharge transitional care program, including a disease-specific care plan, telephone monitoring, hotline counselling and referral to a hospitalist-run clinic. | HCP | ADEs, medication errors and discrepancies;  All-cause mortality; Readmission;  Costs |
| Sides 2012  [152]  USA  N=30 | HOS->PCP Discharge | Initiatives & patient outcome | Medication safety at discharge | To assess the feasibility of medication coaching via telephone after discharge in patients with stroke. | Patients admitted with stroke or TIA with at least 2 medications changes between admission and discharge | Intervention study: non-randomized controlled trial | The medication coach contacted patients post-discharge via phone call to discuss risk factors, review medications and triage patients’ questions to a stroke nurse and/or pharmacist. | HCP & patient | ADEs, medication errors and discrepancies;  All-cause mortality; Readmission;  Cost;  Physical and psychosocial outcomes;  Patient knowledge; Feasibility intervention/ user friendliness;  Care transition measure (CTM) (Coleman et al) |
| Silva 2011  [153]  USA  N=94 | PCP<->HOS Different outpatient transitions | Initiatives & patient outcome | Medication reconciliation | To explore a rule-based medication reconciliation algorithm to accomplish this task across different healthcare transitions. | Patients at the outpatient clinic | Observational study: prospective design | A medication reconciliation algorithm that creates a list of merged prescriptions. | HCP | ADEs, medication errors and discrepancies;  All-cause mortality; Readmission;  Costs |
| Singh 2012  [154]  USA  N=104 | PCP->HOS Referral | HCP outcome | Referral delays | We used a mixed quantitative-qualitative approach to analyse post referral colonoscopy delays in colorectal cancer patients and explored referring physician’s perception of processes surrounding these delays. | Patients with newly diagnosed colorectal cancer | Observational study: retrospective design |  | HCP | ADEs, medication errors and discrepancies;  All-cause mortality; Readmission;  Costs |
| Smith 2015 [155]  USA  N=90 | PCP<->HOS admission and discharge | Patient outcome | Medication reconciliation | To assess if the pharmacy department should be more involved in the medication reconciliation process to assist in the reduction of medication errors that occur during transition of care points in the hospital setting. | Adult patients with at least 5 medications | Observational study: prospective design |  | HCP | ADEs, medication errors and discrepancies |
| Soong 2014  [156]  Canada  N=214 | HOS->PCP Discharge | HCP & patient outcome | Discharge safety | To examine the effect of a 72-hour post-discharge phone call on the patient’s transition of care experience. | Adult general medical patients discharged home after hospitalization | Intervention study: RCT | A 72-hour post-discharge follow-up telephone call | HCP & patient | ADEs, medication errors and discrepancies; All-cause mortality; Readmission;  Costs;  Patient knowledge;  Care transition measure (CTM) (Coleman et al.) |
| Spalek 1999  [157]  USA  N=39 | PCP<->HOS Overall continuity | Patient outcome | Continuity of care: anticoagulation (care chain) | To provide a continuum of patient care, decrease ED visits, decrease episodes of hospitalization, and increase patient satisfaction and quality of life. | Patients using anticoagulation | Intervention study: before- after design | An integrated anticoagulation health system in which a pharmacist is authorized to perform point-of-care testing for pro-trombin times, adjust doses of anticoagulants, order vitamin K, and schedule return visits per established guidelines. | HCP & patient | ADEs, medication errors and discrepancies;  All-cause mortality; Readmission;  Costs;  Physical and psychosocial outcomes;  Patient knowledge; Care transition measure (CTM) (Coleman et al.) |
| Spehar 2005  [158]  USA  N=1845 | HOS->PCP Discharge | Patient outcome | Discharge safety | To identify ways to maximize improvement in post-discharge patient outcomes. This research targeted patients at risk for unscheduled readmissions, examined delivery system processes, and identified possible interventions for providing more seamless care. | Patients at risk for unscheduled readmissions | Observational study: retrospective + prospective design |  | HCP & patient | ADEs, medication errors and discrepancies;  All-cause mortality; Readmission;  Costs;  Patient knowledge |
| Stafford 2011  [159]  Australia  N= 268 | HOS->PCP Discharge | Patient outcome | Discharge safety: anticoagulation | To evaluate the clinical outcomes of a collaborative, home-based post-discharge warfarin management service adapted from the Australian Home Medicines Review (HMR) program. | Patients discharged from the hospital and newly initiated on or continuing warfarin therapy | Intervention study: non-randomized controlled trial | A post-discharge service of 2 or 3 home visits by a trained pharmacist in their first 8 to 10 days post-discharge. The post-discharge service involved point-of-care international normalized ratio (INR) monitoring, warfarin education, and an HMR, in collaboration with the patient’s GP and community pharmacist. | HCP | ADEs, medication errors and discrepancies;  All-cause mortality; Readmission;  Costs |
| Staveski 2016 [160]  India  N=68 | HOS->PCP Discharge | Patient outcome | Discharge preparedness | To compare parent and nurse perceptions of parent readiness for discharge after a structured nurse-led parent discharge teaching programme in India. | Parents of children who underwent cardiac surgery and nurses caring for children after cardiac  surgery | Intervention study: before- after design | A parent education discharge instruction programme. | HCP & patient | Patient care experiences;  Patient knowledge; Questionnaires: related concepts and non-validated questionnaires |
| Stein 2014  [161]  Israel  N=323 | HOS->PCP Discharge | HCP outcome | Discharge safety/ discharge summaries | To evaluate a quality improvement model and training program for writing postoperative discharge summaries for three surgical procedures. | Patients who had been hospitalized for elective surgery | Intervention study: before- after design | A training session concerning the required content of the discharge summaries followed by random reviews and feedback on discharge summaries by the senior attending doctors. | HCP | ADEs, medication errors and discrepancies;  All-cause mortality; Readmission;  Costs |
| Stella 2016 [162]  USA  N=131 | HOS->PCP Discharge | HCP & patient outcome | Discharge safety | To determine whether treating hospitalists  can identify and address early post-discharge problems through a structured telephone call. | Patients discharged from a general internal medicine service of a university-affiliated public safety net  hospital | Observational study: prospective design | A post-discharge hospitalist call. | HCP & patient | ED visit;  Productivity;  Harm and adverse events |
| Steurbaut 2010  [163]  Belgium  N=197 | PCP<->HOS Admission and discharge | HCP & patient outcome | Medication reconciliation | To assess the clinical pharmacist’s performance in obtaining patients’ medication histories and in reconciling these data with the medical records and medication orders and whether the patients’ residential situation prior to hospitalization influences the number of drug discrepancies. | Elderly patients of a acute geriatric ward of a Belgian university hospital, who are prescribed at least 1 prescription drug | Observational study: prospective design | Medication reconciliation at admission by a clinical pharmacist. | HCP | ADEs, medication errors and discrepancies;  All-cause mortality; Readmission;  Costs |
| Stone 2009  [164]  USA  N=23 | PCP->HOS Admission | Patient outcome | Medication reconciliation at admission | To evaluate admission medication reconciliation in children with medically complex conditions by determining the availability and accuracy of five information sources and characterising admitting order errors. | Newly admitted children with medically complex conditions | Observational study: prospective design | Medication reconciliation at hospital admission using information from parents (and patient if appropriate), primary care provider, community-based pharmacy, current admission history and physical exam note, and admission electronic medical record. | HCP | ADEs, medication errors and discrepancies;  All-cause mortality; Readmission;  Costs |
| Stuffken 2008  [165]  the Netherlands N=17362 | HOS->PCP Discharge | Patient outcome | Medication safety | To assess the association between hospitalization and medication therapy discontinuities. | Hospitalised patients | Observational study: retrospective design |  | HCP | ADEs, medication errors and discrepancies;  All-cause mortality; Readmission;  Costs |
| Tamblyn 2012  [166]  Canada  Sample size calculation: N=3714 | HOS->PCP Discharge | Patient outcome | Medication reconciliation at discharge | To determine if electronically enabled discharge reconciliation will reduce the risk of ADEs, emergency room visits and readmissions 30 days post-discharge compared with usual care. | Adult patients admitted to a general or intensive care medical or surgical unit, using prescription medication at admission, and discharged alive. | Intervention study: RCT | Electronic retrieval of the community drug list, combined with electronic discharge reconciliation. | HCP & patient | ADEs, medication errors and discrepancies;  All-cause mortality; Readmission;  Costs |
| Tan 2014  [167]  Australia N=219 | HOS->PCP Discharge | HCP outcome | Medication safety at discharge | To assess the accuracy of documentation of medication changes and expectations with regard to follow up from an acute assessment unit of a tertiary metropolitan hospital. | All patients admitted to an acute assessment unit | Observational study: retrospective design |  | HCP | ADEs, medication errors and discrepancies;  All-cause mortality; Readmission;  Costs;  Harm and adverse events |
| Tan 2015 [168]  Australia  N=722 | HOS->PCP Discharge | HCP outcome | Quality of discharge summary | To assess the role of feedback and token incentives in improving discharge documentation. | Junior medical staff  who compile discharge summaries | Intervention study: before- after design | Discharge summaries of junior medical staff was edited and fed back and positive incentives were given | HCP | Quality and timeliness of discharge summary;  HCP satisfaction and opinions |
| Triller 2005  [169]  USA  N=4250 | HOS->PCP Discharge | Patient outcome | Medication safety at discharge | The prevalence of risk factors for ADEs in patients discharged from the hospital | Patients 21 years and older, discharged from hospital, who were admitted to maternity or same day surgery units, or who had an actual or implied diagnosis of HIV infection or AIDS | Observational study: retrospective design |  | HCP | ADEs, medication errors and discrepancies;  All-cause mortality; Readmission;  Costs;  Harm and adverse events |
| Tsilimingras  2015  [170]  USA  N=684 | HOS->PCP Discharge | Patient outcome | Discharge safety | We aimed to determine the rate of post-discharge adverse events, classify the types of post-discharge adverse events, and identify risk factors for post-discharge adverse events in urban and rural patients. | Adult patients at risk for post-discharge adverse events | Observational study: prospective design |  | HCP & patient | Harm and adverse events |
| Uijen 2013  [171]  the Netherlands  N=519 | PCP<->HOS Overall continuity | HCP outcome | Continuity of care | To explore the level of experienced continuity of care of patients at risk for depression in primary care, and compare these to those of patients with heart failure. | Patients at risk for depression and chronic heart failure patients | Observational study: cross-sectional design |  | Patient | Interprofessional collaboration and communication;  Patient care experiences; Questionnaires: related concepts and non-validated questionnaires |
| Uitvlugt 2015 [172]  the Netherlands  N=99 | HOS->PCP Discharge | HCP & patient outcome | Medication safety at discharge | To assess the completeness of medication-related information in discharge letters and post-discharge GP-overviews. | Patients discharged from the departments of neurology, cardiology, pulmonology and internal medicine | Observational study: prospective design |  | HCP | ADEs, medication errors and discrepancies; Quality and timeliness of discharge summary |
| van Melle 2016 [173]  the Netherlands  N=13 | PCP<->HOS All transitions of care | Patient outcome | Transitional patient safety | To investigate whether transitional incidents can be identified from the medical records of the GPs and the hospital and to assess the concordance of transitional incidents between medical records and patient interviews. | Patients that have experienced or have high risk for transitional incidents | Observational study: retrospective design |  | HCP & patient | Harm and adverse events |
| Varkey 2007  [174]  USA  N=102 | PCP<->HOS Admission and discharge | Patient outcome | Medication reconciliation | To assess the effectiveness of a multidisciplinary medication reconciliation process was studied in an inpatient family medicine unit of an academic hospital centre. | All patients admitted a family medicine department | Intervention study: RCT | Medication reconciliation at admission and discharge by a pharmacist and physician. The reconciled admission and discharge medication lists was included in the hospital summary. | HCP | ADEs, medication errors and discrepancies; Length of hospital stay |
| Villani 2014  [175]  Italy  N=80 | HOS->PCP Discharge | Patient & organisational outcome | Discharge safety | To assess the clinical efficacy and cost of this system. | Patients with chronic heart failure leaving hospital after being treated for clinical instability. | Intervention study: RCT | A handheld PDA for the patient which kept in touch daily with the monitoring centre, communicating with the patient by asking simple questions about their symptoms and giving information and counselling through visual and audible reminders. | HCP & patient | Cause-specific mortality; Admission and inpatient care use; Specific disease-related outcomes;  Costs;  Patient adherence; Physical and psychosocial outcomes |
| Vira 2006  [176]  Canada  N=60 | PCP<->HOS Admission and discharge | Patient outcome | Medication reconciliation | To describe the potential impact of a medication reconciliation process to identify and rectify medication errors at the time of hospital admission and discharge. | Patients admitted to an acute care unit | Observational study: prospective design | A study pharmacist conducted a medication reconciliation process at admission and discharge using information from patient, HCPs, and/or outpatient healthcare providers. | HCP | ADEs, medication errors and discrepancies |
| Vrijhoef 2001  [177]  the Netherlands  N=99 | PCP<->HOS Overall continuity | Patient outcome | Substitution to a nurse practitioner | To assess the effects on quality of care, in terms of patient outcomes, when tasks in the care for outpatients with stable type 2 diabetes are transferred from internist to nurse specialist and from outpatient clinic to general practice. | Patients diagnosed with noninsulin dependent diabetes, without serious co-morbidity treated by a hospitalist | Intervention study: non-randomized controlled trial | Substitution of patient care from hospital internal medicine outpatient care to a nurse specialist in general practice. | HCP & patient | Visits to primary care; Admission and inpatient care use; Specific disease-related outcomes;  Patient adherence; Patient satisfaction; Physical and psychosocial outcomes;  Patient knowledge; Questionnaires: related concepts and non-validated questionnaires |
| Waring 2012  [178]  USA  Sample size calculation: N=2500 | HOS->PCP Discharge | Patient outcome | Quality of discharge | To advance the science of acute coronary syndromes by examining the determinants and outcomes of the quality of transition from hospital to community and by quantifying the impact of potentially modifiable characteristics associated with decreased quality of life, rehospitalisation, and mortality. | Patients hospitalized with acute coronary syndromes | Observational study: prospective design |  | Patient | All-cause mortality; Readmission; Admission and inpatient care use; ED visits;  Patient care experiences;  Physical and psychosocial outcomes |
| Warsame 2016 [179]  USA  N= not reported | HOS->PCP Discharge | HCP & patient outcome | Discharge safety | To develop a care model to decrease incidence of preventable errors in the complex multidisciplinary care of haematology inpatients at the time of discharge. | Discharged haematology Patients Receiving Chemotherapy | Intervention study: before- after design | A multidisciplinary discharge checklist. | HCP | Readmission;  Quality and timeliness of discharge summary; Guideline use |
| Watkins 2012  [180]  USA  N=292 | HOS->PCP Discharge | HCP & patient outcome | Discharge safety | To describe a social-worker navigator transitional care model for at-risk seniors being discharged from hospital to home. | Frail elderly patients at high risk of readmission | Observational study: prospective design | Post-discharge home visits to evaluate the home environment, assess medical management, and make referrals for other services. | Patient | Readmission;  Costs;  Patient satisfaction; Physical and psychosocial outcomes |
| Weiner 2010  [181]  USA  N=6785 | PCP->HOS Referral | Organisational outcome | Referral quality and timeliness of appointment after referral | To determine the extent to which referral-based consultations were completed in a US medical institution. Factors associated with completion were identified. | Elderly patients receiving health care in an integrated, urban, tax-supported medical institution on an acADEsmic campus | Observational study: retrospective design |  | HCP | Referral: timeliness and inappropriateness |
| Were 2009  [182]  USA  N=668 | HOS->PCP Discharge | HCP outcome | Discharge safety; pending tests | To determine the adequacy with which hospital discharge summaries document tests with pending results and the appropriate follow-up providers. | Patients with pending laboratory tests at discharge | Observational study: retrospective design |  | HCP | Diagnostic test follow up;  Quality and timeliness of discharge summary |
| Williams 2015 [183]  UK  N=598 | HOS->PCP Discharge | Patient outcome | Discharge safety | To describe the frequency and types of patient safety incidents associated with discharge from secondary to primary care, and commonly described contributory factors to identify recommendations for practice. | Patients that have experienced patient safety incidents associated with discharge from  secondary to primary care reported to a reporting system | Observational study: cross-sectional design |  | HCP | Harm and adverse events |
| Wilson 2001  [184]  Australia N=569 | HOS->PCP Discharge | HCP outcome | Discharge information timeliness and accuracy | To examine the reliability, effectiveness, accuracy and timeliness of hospital to GP information transfer by discharge summaries produced in a general public hospital in New South Wales, Australia. | Patients discharged from medical, surgical, gynaecological, paediatric, and aged care | Observational study: retrospective design |  | HCP | Quality and timeliness of discharge summary |
| Witherington  2008  [185]  UK  N=108 | HOS->PCP Discharge | HCP & patient outcome | Communication at discharge | To identify communication gaps at hospital discharge for older people who are readmitted within 28 days; to assess the contribution of incomplete discharge information to readmission; to identify measures that might improve communication at hospital discharge. | Patients aged 75 and over who were readmitted as an emergency within 28 days of previous discharge | Observational study: retrospective design |  | HCP & patient | Drug-related hospital (re) admission;  Patient knowledge |
| Wong 2008  [186]  Canada  N=150 | HOS->PCP Discharge | Patient outcome | Medication reconciliation | To identify, characterize, and assess the clinical impact of unintentional medication discrepancies at hospital discharge. | All internal medicine patients admitted for at least 72 hours to a tertiary care teaching hospital | Observational study: prospective design |  | HCP | ADEs, medication errors and discrepancies |
| Wortman 2008  [187]  USA  N=600 | PCP<->HOS Admission and discharge | Patient outcome | Medication reconciliation | To examine a medication reconciliation program involving physicians, pharmacists, nursing staff, and other personnel at a community hospital. | Patient admitted to hospital | Intervention study: before- after design | Medication reconciliation by a multidisciplinary team (physicians, pharmacists, nursing staff, and other personnel) at admission, transfer, and discharge. | HCP | ADEs, medication errors and discrepancies |
| Yi 2011  [188]  Singapore  N=24000 | PCP->HOS Admission | Patient outcome | Medication reconciliation | This article aims to describe the Tan Tock Seng Hospital medication reconciliation system and to highlight common medication errors occurring following incomplete medication reconciliation. | All patients admitted to hospital | Observational study: prospective design | Medication review by a pharmacist at admission. Any discrepancies in medicines are brought to the doctors’ attention. Patients are also counselled about changes to their medications. | HCP | ADEs, medication errors and discrepancies |
| Young 2013  [189]  Australia  N=775 | HOS->PCP Discharge | HCP & patient outcome | Discharge safety | To investigate the effectiveness of a centralized, nurse-delivered telephone-based service to improve care coordination and patient-reported outcomes after surgery for colorectal cancer. | Adult patients with newly diagnosed colorectal cancer | Intervention study: RCT | Standardized calls from the centrally based nurse after discharge from hospital. | Patient | Readmission;  ED visits;  Patient care experiences;  Patient satisfaction; Physical and psychosocial outcomes; Questionnaires: related concepts and non-validated questionnaires |
| Zhang 2013  [190]  Australia Sample size calculation: N=456 | PCP<->HOS Overall continuity | Patient outcome | Continuity of diabetes care (care chain) | To evaluate the health and clinical benefits of the new model of care, assesses the acceptability of the model to patients, GPs and other health professionals, and examines the cost-effectiveness of the model. | Patients with complex and/or uncontrolled T2DM | Intervention study: RCT | Diabetes care by a multidisciplinary team consisting of an Endocrinologist, advanced-skilled GPs (known as Clinical Fellows), a credentialed diabetes educator and a podiatrist all located together in a community based complex diabetes service. | HCP & patient | Specific disease-related outcomes;  Costs;  Patient adherence; Patient satisfaction; Harm and adverse events;  Physical and psychosocial outcomes;  Patient knowledge |
| Zoni 2012  [191]  Spain  N=162 | PCP->HOS Admission | Patient outcome | Medication reconciliation at admission | To assess the impact of an intervention aimed to decrease unintended discrepancies between patient's usual treatment and medications prescribed on admission to the department of internal medicine. | All patients hospitalized in a department of internal medicine, and whose prescriptions prior to admission included three or more drugs | Intervention study: before- after design | Medication reconciliation at admission by an electronic reconciliation tool that connected patients' usual medication with the electronic prescription program. | HCP | ADEs, medication errors and discrepancies |

ADE=Adverse drug event; AE=Adverse event; DOC-questionnaire= Doctors’ Opinions on Collaboration-questionnaire; ED=Emergency department; GP=General practitioner; HCP=Healthcare professional; PCP=Primary care physician; RCT=Randomised controlled trial.

**REFERENCES**

1. Ahmed S, Harding J. Investigating the procedures, drawbacks and implications of seamless primary-secondary care. Quality in Primary Care. 2005;12:51–55.
2. Akbarov A, Kontopantelis E, Sperrin M, Stocks SJ, Williams R, Rodgers S, Avery A, Buchan I, Ashcroft DM. Primary Care Medication Safety Surveillance with Integrated Primary and Secondary Care Electronic Health Records: A Cross-Sectional Study. Drug Saf. 2015 Jul;38(7):671-82. doi: 10.1007/s40264-015-0304-x.
3. Alassaad A, Gillespie U, Bertilsson M, Melhus H, Hammarlund-Udenaes M. Prescription and transcription errors in multidose-dispensed medications on discharge from hospital: an observational and interventional study. J Eval Clin Pract. 2013 Feb;19(1):185-91. doi: 10.1111/j.1365-2753.2011.01798.x. Epub 2011 Dec 29.
4. Alex S, Adesnew AB, Arundel C, Maron DD, Kerns JC. Medication Errors Despite Using Electronic Health Records: The Value of a Clinical Pharmacist Service in Reducing Discharge-Related Medication Errors. Qual Manag Health Care. 2016 Jan-Mar;25(1):32-7.
5. Alidina S, Rosenthal M, Schneider E, Singer S. Coordination within medical neighborhoods: Insights from the early experiences of Colorado patient-centered medical homes. Health Care Manage Rev. 2016 Apr-Jun;41(2):101-12.
6. Aller MB, Vargas I, Waibel S, Coderch-Lassaletta J, Sánchez-Pérez I, Llopart JR, Colomés L, Ferran M, Garcia-Subirats I, Vázquez Navarrete ML. Factors associated to experienced continuity of care between primary and outpatient secondary care in the Catalan public healthcare system. Gac Sanit. 2013 May-Jun;27(3):207-13. doi: 10.1016/j.gaceta.2012.06.011. Epub 2012 Sep 14.
7. Aller MB, Vargas I, Waibel S, Coderch J, Sánchez-Pérez I, Colomés L, Llopart JR, Ferran M, Vázquez ML. A comprehensive analysis of patients' perceptions of continuity of care and their associated factors. Int J Qual Health Care. 2013 Jul;25(3):291-9. doi: 10.1093/intqhc/mzt010. Epub 2013 Feb 19.
8. Anderson SL, Marrs JC, Vande Griend JP, Hanratty R. Implementation of a clinical pharmacy specialist-managed telephonic hospital discharge follow-up program in a patient-centered medical home. Popul Health Manag. 2013 Aug;16(4):235-41. doi: 10.1089/pop.2012.0070. Epub 2013 Mar 28.
9. Armor BL, Wight AJ, Carter SM. Evaluation of Adverse Drug Events and Medication Discrepancies in Transitions of Care Between Hospital Discharge and Primary Care Follow-Up. J Pharm Pract. 2016 Apr;29(2):132-7. doi: 10.1177/0897190014549836. Epub 2014 Oct 13.
10. Arora VM, Prochaska ML, Farnan JM, D'Arcy MJ 5th, Schwanz KJ, Vinci LM, Davis AM, Meltzer DO, Johnson JK. Problems after discharge and understanding of communication with their primary care physicians among hospitalized seniors: a mixed methods study. J Hosp Med. 2010 Sep;5(7):385-91. doi: 10.1002/jhm.668.
11. Atkinson WL, Frey D. Integration of a medication management model into outcome-based quality improvement: a pilot program in a rural propriety home healthcare agency. Home Health Care Serv Q. 2005;24(1-2):29-45.
12. Azzi M, Constantino M, Pont L, Mcgill M, Twigg S, Krass I. Medication Safety: an audit of medication discrepancies in transferring type 2 diabetes mellitus (T2DM) patients from Australian primary care to tertiary ambulatory care. Int J Qual Health Care. 2014 Aug;26(4):397-403. doi: 10.1093/intqhc/mzu051. Epub 2014 May 19.
13. Balaban RB, Galbraith AA, Burns ME, Vialle-Valentin CE, Larochelle MR, Ross-Degnan D. A Patient Navigator Intervention to Reduce Hospital Readmissions among High-Risk Safety-Net Patients: A Randomized Controlled Trial. J Gen Intern Med. 2015 Jul;30(7):907-15. doi: 10.1007/s11606-015-3185-x. Epub 2015 Jan 24.
14. Baldo V, Lombardi S, Cocchio S, Rancan S, Buja A, Cozza S, Marangon C, Furlan P, Cristofoletti M. Diabetes outcomes within integrated healthcare management programs. Prim Care Diabetes. 2015 Feb;9(1):54-9. doi: 10.1016/j.pcd.2014.03.005. Epub 2014 Apr 16.
15. Balling L, Erstad BL, Weibel K. Impact of a transition-of-care pharmacist during hospital discharge. J Am Pharm Assoc. 2015 Jul-Aug;55(4):443-8. doi: 10.1331/JAPhA.2015.14087.
16. Balogun SA, Rose K, Thomas S, Owen J, Brashers V. Innovative interprofessional geriatric education for medical and nursing students: focus on transitions in care. QJM. 2015 Jun;108(6):465-71. doi: 10.1093/qjmed/hcu226. Epub 2014 Oct 31.
17. Bauer A, Vordermark D, Seufferlein T, Schmoll HJ, Dralle H, Mau W, Unverzagt S, Boese S, Fach EM, Landenberger M. Trans-sectoral care in patients with colorectal cancer: Protocol of the randomized controlled multi-center trial Supportive Cancer Care Networkers (SCAN). BMC Cancer. 2015 Dec 22;15:997. doi: 10.1186/s12885-015-2002-6.
18. Belda-Rustarazo S, Cantero-Hinojosa J, Salmeron-García A, González-García L, Cabeza-Barrera J, Galvez J. Medication reconciliation at admission and discharge: an analysis of prevalence and associated risk factors. Int J Clin Pract. 2015 Nov;69(11):1268-74. doi: 10.1111/ijcp.12701. Epub 2015 Jul 22.
19. Berendsen AJ, Kuiken A, Benneker WH, Meyboom-de Jong B, Voorn TB, Schuling J. How do general practitioners and specialists value their mutual communication? A survey. BMC Health Serv Res. 2009 Aug 8;9:143. doi: 10.1186/1472-6963-9-143.
20. Bergkvist A, Midlöv P, Höglund P, Larsson L, Bondesson A, Eriksson T. Improved quality in the hospital discharge summary reduces medication errors--LIMM: Landskrona Integrated Medicines Management. Eur J Clin Pharmacol. 2009 Oct;65(10):1037-46. doi: 10.1007/s00228-009-0680-1. Epub 2009 Jun 26.
21. Biese K, Lamantia M, Shofer F, McCall B, Roberts E, Stearns SC, Principe S, Kizer JS, Cairns CB, Busby-Whitehead J. A randomized trial exploring the effect of a telephone call follow-up on care plan compliance among older adults discharged home from the emergency department. Acad Emerg Med. 2014 Feb;21(2):188-95. doi: 10.1111/acem.12308.
22. Bishop MA, Cohen BA, Billings LK, Thomas EV. Reducing errors through discharge medication reconciliation by pharmacy services. Am J Health Syst Pharm. 2015 Sep 1;72(17 Suppl 2):S120-6. doi: 10.2146/sp150021.
23. Bloom-Feshbach K, Casey D, Schulson L, Gliatto P, Giftos J, Karani R. Health Literacy in Transitions of Care: An Innovative Objective Structured Clinical Examination for Fourth-Year Medical Students in an Internship Preparation Course. J Gen Intern Med. 2016 Feb;31(2):242-6. doi: 10.1007/s11606-015-3513-1. Epub 2015 Oct 9.
24. Bonnet-Zamponi D, d'Arailh L, Konrat C, Delpierre S, Lieberherr D, Lemaire A, Tubach F, Lacaille S, Legrain S; Optimization of Medication in AGEd study group. Drug-related readmissions to medical units of older adults discharged from acute geriatric units: results of the Optimization of Medication in AGEd multicenter randomized controlled trial. J Am Geriatr Soc. 2013 Jan;61(1):113-21. doi: 10.1111/jgs.12037. Epub 2012 Dec 18.
25. Braund R, Coulter CV, Bodington AJ, Giles LM, Greig AM, Heaslip LJ, Marshall BJ. Drug related problems identified by community pharmacists on hospital discharge prescriptions in New Zealand. Int J Clin Pharm. 2014 Jun;36(3):498-502. doi: 10.1007/s11096-014-9935-8. Epub 2014 Apr 4.
26. Bray-Hall S, Schmidt K, Aagaard E. Toward safe hospital discharge: a transitions in care curriculum for medical students. J Gen Intern Med. 2010 Aug;25(8):878-81. doi: 10.1007/s11606-010-1364-3. Epub 2010 May 5.
27. Brener SS, Bronksill SE, Comrie R, Huang A, Bell CM. Association between in-hospital supportive visits by primary care physicians and patient outcomes: A population-based cohort study. J Hosp Med. 2016 Jun;11(6):418-24. doi: 10.1002/jhm.2561. Epub 2016 Feb 23.
28. Burns ME, Galbraith AA, Ross-Degnan D, Balaban RB. Feasibility and evaluation of a pilot community health worker intervention to reduce hospital readmissions. Int J Qual Health Care. 2014 Aug;26(4):358-65. doi: 10.1093/intqhc/mzu046. Epub 2014 Apr 16.
29. Callen JL, Alderton M, McIntosh J. Evaluation of electronic discharge summaries: a comparison of documentation in electronic and handwritten discharge summaries. Int J Med Inform. 2008 Sep;77(9):613-20. doi: 10.1016/j.ijmedinf.2007.12.002. Epub 2008 Feb 21.
30. Callen J, McIntosh J, Li J. Accuracy of medication documentation in hospital discharge summaries: A retrospective analysis of medication transcription errors in manual and electronic discharge summaries. Int J Med Inform. 2010 Jan;79(1):58-64. doi: 10.1016/j.ijmedinf.2009.09.002. Epub 2009 Oct 3.
31. Carter BL, Farris KB, Abramowitz PW, Weetman DB, Kaboli PJ, Dawson JD, James PA, Christensen AJ, Brooks JM. The Iowa Continuity of Care study: Background and methods. Am J Health Syst Pharm. 2008 Sep 1;65(17):1631-42. doi: 10.2146/ajhp070600.
32. Cavanaugh JJ, Lindsey KN, Shilliday BB, Ratner SP. Pharmacist-coordinated multidisciplinary hospital follow-up visits improve patient outcomes. J Manag Care Spec Pharm. 2015 Mar;21(3):256-60.
33. Chan B, Goldman LE, Sarkar U, Schneidermann M, Kessell E, Guzman D, Critchfield J, Kushel M. The Effect of a Care Transition Intervention on the Patient Experience of Older Multi-Lingual Adults in the Safety Net: Results of a Randomized Controlled Trial. J Gen Intern Med. 2015 Dec;30(12):1788-94. doi: 10.1007/s11606-015-3362-y.
34. Chevalier BA, Parker DS, MacKinnon NJ, Sketris I. Nurses' perceptions of medication safety and medication reconciliation practices. Nurs LeADEsrsh (Tor Ont). 2006 Sep;19(3):61-72.
35. Coleman EA, Smith JD, Frank JC, Min SJ, Parry C, Kramer AM. Preparing patients and caregivers to participate in care delivered across settings: the Care Transitions Intervention. J Am Geriatr Soc. 2004 Nov;52(11):1817-25.
36. Coleman EA, Min SJ, Chomiak A, Kramer AM. Posthospital care transitions: patterns, complications, and risk identification. Health Serv Res. 2004 Oct;39(5):1449-65.
37. Coleman EA, Smith JD, Raha D, Min SJ. Posthospital medication discrepancies: prevalence and contributing factors. Arch Intern Med. 2005 Sep 12;165(16):1842-7.
38. Coleman EA, Parry C, Chalmers S, Min SJ. The care transitions intervention: results of a randomized controlled trial. Arch Intern Med. 2006 Sep 25;166(17):1822-8.
39. Dalal AK, Roy CL, Poon EG, Williams DH, Nolido N, Yoon C, Budris J, Gandhi T, Bates DW, Schnipper JL. Impact of an automated email notification system for results of tests pending at discharge: a cluster-randomized controlled trial. J Am Med Inform Assoc. 2014 May-Jun;21(3):473-80. doi: 10.1136/amiajnl-2013-002030. Epub 2013 Oct 23.
40. Daley CM. A hybrid transitional care program. Crit Pathw Cardiol. 2010 Dec;9(4):231-4. doi: 10.1097/HPC.0b013e3182018838.
41. Dichmann Sorknaes A. The Effect of Tele-Consultation Between a Hospital-Based Nurse and a COPD Patient. Stud Health Technol Inform. 2016;225:883-4.
42. Donaho EK, Hall AC, Gass JA, Elayda MA, Lee VV, Paire S, Meyers DE. Protocol-Driven Allied Health Post-Discharge Transition Clinic to Reduce Hospital Readmissions in Heart Failure. J Am Heart Assoc. 2015 Dec 23;4(12). pii: e002296. doi: 10.1161/JAHA.115.002296.
43. Douglas AM, Letts LJ, Richardson JA, Eva KW. Validity of predischarge measures for predicting time to harm in older adults. Can J Occup Ther. 2013 Feb;80(1):19-27.
44. Dunn AS, Shetreat-Klein A, Berman J, Cho HJ, Stein L, Lewis C, Hamilton S, To S, Francaviglia P, Kannry J.Improving transitions of care for patients on warfarin: The safe transitions anticoagulation report. J Hosp Med. 2015 Sep;10(9):615-8. doi: 10.1002/jhm.2393.
45. Dutton, K., Hedger, N., Wills, S., Brown, D., Davies, P. Prevent medication errors on admission. Clinical Governance: An International Journal 2003 8:128-137.
46. Elliott RA, Tran T, Taylor SE, Harvey PA, Belfrage MK, Jennings RJ, Marriott JL. Impact of a pharmacist-prepared interim residential care medication administration chart on gaps in continuity of medication management after discharge from hospital to residential care: a prospective pre- and post-intervention study (MedGap Study). BMJ Open. 2012 May 25;2(3). pii: e000918. doi: 10.1136/bmjopen-2012-000918. Print 2012.
47. Ellitt GR, Engblom E, Aslani P, Westerlund T, Chen TF. Drug related problems after discharge from an Australian teaching hospital. Pharm World Sci. 2010 Oct;32(5):622-30. doi: 10.1007/s11096-010-9406-9. Epub 2010 Jun 26.
48. Eskildsen MA, Chakkalakal R, Flacker JM. Use of a virtual classroom in training fourth-year medical students on care transitions. J Hosp Med. 2012 Jan;7(1):14-21. doi: 10.1002/jhm.915. Epub 2011 Oct 3.
49. Farris KB, Carter BL, Xu Y, Dawson JD, Shelsky C, Weetman DB, Kaboli PJ, James PA, Christensen AJ, Brooks JM. Effect of a care transition intervention by pharmacists: an RCT. BMC Health Serv Res. 2014 Sep 18;14:406. doi: 10.1186/1472-6963-14-406.
50. Fertig A, Roland M, King H, Moore T. Understanding variation in rates of referral among general practitioners: are inappropriate referrals important and would guidelines help to reduce rates? BMJ. 1993 Dec 4;307(6917):1467-70.
51. Flink M, Bergenbrant Glas S, Airosa F, Öhlén G, Barach P, Hansagi H, Brommels M, Olsson M. Patient-centered handovers between hospital and primary health care: an assessment of medical records. Int J Med Inform. 2015 May;84(5):355-62. doi: 10.1016/j.ijmedinf.2015.01.009. Epub 2015 Jan 22.
52. Forster AJ, Murff HJ, Peterson JF, Gandhi TK, Bates DW. The incidence and severity of adverse events affecting patients after discharge from the hospital. Ann Intern Med. 2003 Feb 4;138(3):161-7.
53. Forster AJ, Clark HD, Menard A, Dupuis N, Chernish R, Chandok N, Khan A, van Walraven C. Adverse events among medical patients after discharge from hospital. CMAJ. 2004 Feb 3;170(3):345-9.
54. Forster AJ, Murff HJ, Peterson JF, Gandhi TK, Bates DW. Adverse drug events occurring following hospital discharge. J Gen Intern Med. 2005 Apr;20(4):317-23.
55. Forster AJ, Rose NG, van Walraven C, Stiell I. Adverse events following an emergency department visit. Qual Saf Health Care. 2007 Feb;16(1):17-22.
56. Foust JB, Naylor MD, Bixby MB, Ratcliffe SJ. Medication problems occurring at hospital discharge among older adults with heart failure. Res Gerontol Nurs. 2012 Jan;5(1):25-33. doi: 10.3928/19404921-20111206-04. Epub 2011 Dec 29.
57. Frydenberg K, Brekke M. Poor communication on patients' medication across health care levels leads to potentially harmful medication errors. Scand J Prim Health Care. 2012 Dec;30(4):234-40. doi: 10.3109/02813432.2012.712021. Epub 2012 Oct 10.
58. García-Molina Sáez C, Urbieta Sanz E, Madrigal de Torres M, Vicente Vera T, Pérez Cárceles MD. Computerized pharmaceutical intervention to reduce reconciliation errors at hospital discharge in Spain: an interrupted time-series study. J Clin Pharm Ther. 2016 Apr;41(2):203-8. doi: 10.1111/jcpt.12365. Epub 2016 Feb 25.
59. Gardella JE, Cardwell TB, Nnadi M. Improving medication safety with accurate preadmission medication lists and post-discharge education. Jt Comm J Qual Patient Saf. 2012 Oct;38(10):452-8.
60. Gardner R, Li Q, Baier RR, Butterfield K, Coleman EA, Gravenstein S. Is implementation of the care transitions intervention associated with cost avoidance after hospital discharge? J Gen Intern Med. 2014 Jun;29(6):878-84. doi: 10.1007/s11606-014-2814-0. Epub 2014 Mar 4.
61. Ghatnekar O, Bondesson A, Persson U, Eriksson T. Health economic evaluation of the Lund Integrated Medicines Management Model (LIMM) in elderly patients admitted to hospital. BMJ Open. 2013 Jan 10;3(1). pii: e001563. doi: 10.1136/bmjopen-2012-001563.
62. Gilbert AV, Patel B, Morrow M, Williams D, Roberts MS, Gilbert AL. Providing community-based health practitioners with timely and accurate discharge medicines information. BMC Health Serv Res. 2012 Dec 10;12:453. doi: 10.1186/1472-6963-12-453.
63. Glintborg B, Andersen SE, Dalhoff K. Insufficient communication about medication use at the interface between hospital and primary care. Qual Saf Health Care. 2007 Feb;16(1):34-9.
64. Graumlich JF, Novotny NL, Nace GS, Aldag JC. Patient and physician perceptions after software-assisted hospital discharge: cluster randomized trial. J Hosp Med. 2009 Jul;4(6):356-63. doi: 10.1002/jhm.565.
65. Graumlich JF, Novotny NL, Stephen Nace G, Kaushal H, Ibrahim-Ali W, Theivanayagam S, William Scheibel L, Aldag JC. Patient readmissions, emergency visits, and adverse events after software-assisted discharge from hospital: cluster randomized trial. J Hosp Med. 2009 Sep;4(7):E11-9. doi: 10.1002/jhm.469.
66. Gray S, Urwin M, Woolfrey S, Harrington B, Cox J. Copying hospital discharge summaries to practice pharmacists: does this help implement treatment plans? Qual Prim Care. 2008;16(5):327-34.
67. Grimes T, Delaney T, Duggan C, Kelly JG, Graham IM. Survey of medication documentation at hospital discharge: implications for patient safety and continuity of care. Ir J Med Sci. 2008 Jun;177(2):93-7. doi: 10.1007/s11845-008-0142-2. Epub 2008 Apr 15.
68. Grimes TC, Duggan CA, Delaney TP, Graham IM, Conlon KC, Deasy E, Jago-Byrne MC, O' Brien P. Medication details documented on hospital discharge: cross-sectional observational study of factors associated with medication non-reconciliation. Br J Clin Pharmacol. 2011 Mar;71(3):449-57. doi: 10.1111/j.1365-2125.2010.03834.x.
69. Hamar B, Rula EY, Wells AR, Coberley C, Pope JE, Varga D. Impact of a scalable care transitions program for readmission avoidance. Am J Manag Care. 2016 Jan;22(1):28-34.
70. Hanlon JT, Pieper CF, Hajjar ER, Sloane RJ, Lindblad CI, Ruby CM, SchmADEsr KE. Incidence and predictors of all and preventable adverse drug reactions in frail elderly persons after hospital stay. J Gerontol A Biol Sci Med Sci. 2006 May;61(5):511-5.
71. Hansen LO, Greenwald JL, Budnitz T, Howell E, Halasyamani L, Maynard G, Vidyarthi A, Coleman EA, Williams MV. Project BOOST: effectiveness of a multihospital effort to reduce rehospitalization. J Hosp Med. 2013 Aug;8(8):421-7. doi: 10.1002/jhm.2054. Epub 2013 Jul 22.
72. Hastings SN, Barrett A, Weinberger M, Oddone EZ, Ragsdale L, Hocker M, SchmADEsr KE. Older patients' understanding of emergency department discharge information and its relationship with adverse outcomes. J Patient Saf. 2011 Mar;7(1):19-25. doi: 10.1097/PTS.0b013e31820c7678.
73. Hawes EM, Maxwell WD, White SF, Mangun J, Lin FC. Impact of an outpatient pharmacist intervention on medication discrepancies and health care resource utilization in posthospitalization care transitions. J Prim Care Community Health. 2014 Jan 1;5(1):14-8. doi: 10.1177/2150131913502489. Epub 2013 Sep 17.
74. Hazen AC, Sloeserwij VM, Zwart DL, de Bont AA, Bouvy ML, de Gier JJ, de Wit NJ, Leendertse AJ. Design of the POINT study: Pharmacotherapy Optimisation through Integration of a Non-dispensing pharmacist in a primary care Team (POINT). BMC Fam Pract. 2015 Jul 2;16:76. doi: 10.1186/s12875-015-0296-8.
75. Hellström LM, Bondesson Å, Höglund P, Eriksson T. Errors in medication history at hospital admission: prevalence and predicting factors. BMC Clin Pharmacol. 2012 Apr 3;12:9. doi: 10.1186/1472-6904-12-9.
76. Heyworth L, Paquin AM, Clark J, Kamenker V, Stewart M, Martin T, Simon SR. Engaging patients in medication reconciliation via a patient portal following hospital discharge. J Am Med Inform Assoc. 2014 Feb;21(e1):e157-62. doi: 10.1136/amiajnl-2013-001995. Epub 2013 Sep 13.
77. Hohmann C, Neumann-Haefelin T, Klotz JM, Freidank A, Radziwill R. Providing systematic detailed information on medication upon hospital discharge as an important step towards improved transitional care. J Clin Pharm Ther. 2014 Jun;39(3):286-91. Epub 2014 Feb 18.
78. Holland DE, Hemann MA. Standardizing hospital discharge planning at the Mayo Clinic. Jt Comm J Qual Patient Saf. 2011 Jan;37(1):29-36.
79. Horwitz LI, Jenq GY, Brewster UC, Chen C, KanADEs S, Van Ness PH, Araujo KL, Ziaeian B, Moriarty JP, Fogerty RL, Krumholz HM. Comprehensive quality of discharge summaries at an acADEsmic medical center. J Hosp Med. 2013 Aug;8(8):436-43. doi: 10.1002/jhm.2021. Epub 2013 Mar 22.
80. Hu SH, Capezuti E, Foust JB, Boltz MP, Kim H. Medication discrepancy and potentially inappropriate medication in older Chinese-American home-care patients after hospital discharge. Am J Geriatr Pharmacother. 2012 Oct;10(5):284-95. doi: 10.1016/j.amjopharm.2012.08.001. Epub 2012 Sep 1.
81. Huynh C, Wong IC, Tomlin S, Halford E, Jani Y, Ghaleb M. An evaluation of paediatric medicines reconciliation at hospital discharge into the community. Int J Pharm Pract. 2016 May;24(3):196-202. doi: 10.1111/ijpp.12229. Epub 2015 Dec 16.
82. Jack BW, Chetty VK, Anthony D, Greenwald JL, Sanchez GM, Johnson AE, Forsythe SR, O'Donnell JK, Paasche-Orlow MK, Manasseh C, Martin S, Culpepper L. A reengineered hospital discharge program to decrease rehospitalization: a randomized trial. Ann Intern Med. 2009 Feb 3;150(3):178-87.
83. Julian S, Naftalin NJ, Clark M, Szczepura A, Rashid A, Baker R, Taub N, Habiba M. An integrated care pathway for menorrhagia across the primary-secondary interface: patients' experience, clinical outcomes, and service utilisation. Qual Saf Health Care. 2007 Apr;16(2):110-5.
84. Kanaan AO, Donovan JL, Duchin NP, Field TS, Tjia J, Cutrona SL, Gagne SJ, Garber L, Preusse P, Harrold LR, Gurwitz JH. Adverse drug events after hospital discharge in older adults: types, severity, and involvement of Beers Criteria Medications. J Am Geriatr Soc. 2013 Nov;61(11):1894-9. doi: 10.1111/jgs.12504. Epub 2013 Oct 1.
85. Kantelhardt P, Giese A, Kantelhardt SR. Medication reconciliation for patients undergoing spinal surgery. Eur Spine J. 2016 Mar;25(3):740-7. doi: 10.1007/s00586-015-3878-1. Epub 2015 Mar 21.
86. Karapinar-Carkit F, Borgsteede SD, Zoer J, Siegert C, van Tulder M, Egberts AC, van den Bemt PM. The effect of the COACH program(Continuity Of Appropriate pharmacotherapy, patient Counselling and information transferin Healthcare) on readmission rates in a multicultural population of internal medicine patients. BMC Health Services Research201010:39 DOI: 10.1186/1472-6963-10-39
87. Keers RN, Williams SD, Vattakatuchery JJ, Brown P, Miller J, Prescott L, Ashcroft DM. Medication safety at the interface: evaluating risks associated with discharge prescriptions from mental health hospitals. J Clin Pharm Ther. 2015 Dec;40(6):645-54. doi: 10.1111/jcpt.12328. Epub 2015 Nov 3.
88. Kennedy AG, Chen H, Corriveau M, MacLean CD. Improving population management through pharmacist-primary care integration: a pilot study. Popul Health Manag. 2015 Feb;18(1):23-9. doi: 10.1089/pop.2014.0043. Epub 2014 Jul 16.
89. Kind AJ, Jensen L, Barczi S, Bridges A, Kordahl R, Smith MA, Asthana S. Low-cost transitional care with nurse managers making mostly phone contact with patients cut rehospitalization at a VA hospital. Health Aff (Millwood). 2012 Dec;31(12):2659-68. doi: 10.1377/hlthaff.2012.0366.
90. King TL. The Impact of a Nurse-Driven Evidence-Based Discharge Planning Protocol on Organizational Efficiency and Patient Satisfaction in Patients with Cardiac Implants [Dissertation]. Orlando, FL: Univ of Central Florida; 2008.
91. Knez L, Suskovic S, Rezonja R, Laaksonen R, Mrhar A. The need for medication reconciliation: a cross-sectional observational study in adult patients. Respir Med. 2011 Oct;105 Suppl 1:S60-6. doi: 10.1016/S0954-6111(11)70013-0.
92. Koehler BE, Richter KM, Youngblood L, Cohen BA, Prengler ID, Cheng D, Masica AL. Reduction of 30-day post-discharge hospital readmission or emergency department (ED) visit rates in high-risk elderly medical patients through delivery of a targeted care bundle. J Hosp Med. 2009 Apr;4(4):211-8. doi: 10.1002/jhm.427.
93. Lai CJ, Nye HE, Bookwalter T, Kwan A, Hauer KE. Post-discharge follow-up visits for medical and pharmacy students on an inpatient medicine clerkship. J Hosp Med. 2008 Jan;3(1):20-7. doi: 10.1002/jhm.264.
94. Lalonde L, Lampron AM, Vanier MC, Levasseur P, Khaddag R, Chaar N. Effectiveness of a medication discharge plan for transitions of care from hospital to outpatient settings. Am J Health Syst Pharm. 2008 Aug 1;65(15):1451-7. doi: 10.2146/ajhp070565.
95. Lassere MN, Baker S, Parle A, Sara A, Johnson KR. Improving quality of care and long-term health outcomes through continuity of care with the use of an electronic or paper patient-held portable health file (COMMUNICATE): study protocol for a randomized controlled trial. Trials. 2015 Jun 4;16:253. doi: 10.1186/s13063-015-0760-8.
96. Lee BH, Lehmann CU, Jackson EV, Kost-Byerly S, Rothman S, Kozlowski L, Miller MR, Pronovost PJ, Yaster M. Assessing controlled substance prescribing errors in a pediatric teaching hospital: an analysis of the safety of analgesic prescription practice in the transition from the hospital to home. J Pain. 2009 Feb;10(2):160-6. doi: 10.1016/j.jpain.2008.08.004. Epub 2008 Nov 17.
97. Lesselroth B, Adams S, Felder R, Dorr DA, Cauthers P, Church V, Douglas D. Using consumer-based kiosk technology to improve and standardize medication reconciliation in a specialty care setting. Jt Comm J Qual Patient Saf. 2009 May;35(5):264-70.
98. Lindpaintner LS, Gasser JT, Schramm MS, Cina-Tschumi B, Müller B, Beer JH. Discharge intervention pilot improves satisfaction for patients and professionals. Eur J Intern Med. 2013 Dec;24(8):756-62. doi: 10.1016/j.ejim.2013.08.703. Epub 2013 Sep 26.
99. Lindquist LA, Yamahiro A, Garrett A, Zei C, Feinglass JM. Primary care physician communication at hospital discharge reduces medication discrepancies. J Hosp Med. 2013 Dec;8(12):672-7. doi: 10.1002/jhm.2098. Epub 2013 Nov 1.
100. Lingaratnam S, Aranda S, Pearce T, Kirsa S. A controlled before and after study to evaluate a patient and health professional partnership model towards effective medication reconciliation. J Oncol Pharm Pract. 2013 Mar;19(1):48-56. doi: 10.1177/1078155212451936. Epub 2012 Jul 30.
101. Linsky A, Simon SR. Medication discrepancies in integrated electronic health records. BMJ Qual Saf. 2013 Feb;22(2):103-9. doi: 10.1136/bmjqs-2012-001301. Epub 2012 Oct 25.
102. Manning DM, O'Meara JG, Williams AR, Rahman A, Myhre D, Tammel KJ, Carter LC. 3D: a tool for medication discharge education. Qual Saf Health Care. 2007 Feb;16(1):71-6.
103. Martinussen PE. Referral quality and the cooperation between hospital physicians and general practice: the role of physician and primary care factors. Scand J Public Health. 2013 Dec;41(8):874-82. doi: 10.1177/1403494813498951. Epub 2013 Aug 19.
104. Maslove DM, Leiter RE, Griesman J, Arnott C, Mourad O, Chow CM, Bell CM. Electronic versus dictated hospital discharge summaries: a randomized controlled trial. J Gen Intern Med. 2009 Sep;24(9):995-1001. doi: 10.1007/s11606-009-1053-2. Epub 2009 Jul 16.
105. Mazor K, Roblin DW, Greene SM, Fouayzi H, Gallagher TH. Primary care physicians' willingness to disclose oncology errors involving multiple providers to patients. BMJ Qual Saf. 2016 Oct;25(10):787-95. doi: 10.1136/bmjqs-2015-004353. Epub 2015 Nov 3.
106. McCormick ME, Ward E, Roberson DW, Shah RK, Stachler RJ, Brenner MJ. Life after Tracheostomy: Patient and Family Perspectives on Teaching, Transitions, and Multidisciplinary Teams. Otolaryngol Head Neck Surg. 2015 Dec;153(6):914-20. doi: 10.1177/0194599815599525. Epub 2015 Aug 18.
107. McMillan TE, Allan W, Black PN. Accuracy of information on medicines in hospital discharge summaries. Intern Med J. 2006 Apr;36(4):221-5.
108. McMillan A, Trompeter J, Havrda D, Fox J. Continuity of care between family practice physicians and hospitalist services. J Healthc Qual. 2013 Jan-Feb;35(1):41-9. doi: 10.1111/j.1945-1474.2011.00165.x. Epub 2011 Sep 13.
109. Meran S, Don K, Shah N, Donovan K, Riley S, Phillips AO. Impact of chronic kidney disease management in primary care. QJM. 2011 Jan;104(1):27-34. doi: 10.1093/qjmed/hcq151. Epub 2010 Aug 30.
110. Mesteig M, Helbostad JL, Sletvold O, Røsstad T, Saltvedt I. Unwanted incidents during transition of geriatric patients from hospital to home: a prospective observational study. BMC Health Serv Res. 2010 Jan 4;10:1. doi: 10.1186/1472-6963-10-1.
111. Meyers AG, Salanitro A, Wallston KA, Cawthon C, Vasilevskis EE, Goggins KM, Davis CM, Rothman RL, Castel LD, Donato KM, Schnelle JF, Bell SP, Schildcrout JS, Osborn CY, Harrell FE, Kripalani S. Determinants of health after hospital discharge: rationale and design of the Vanderbilt Inpatient Cohort Study (VICS). BMC Health Serv Res. 2014 Jan 8;14:10. doi: 10.1186/1472-6963-14-10.
112. Mishra AK, Bhattarai S, Bhurtel P, Bista NR, Shrestha P, Thakali K, Banthia P, Pathak SR. Need for improvement of medical records. JNMA J Nepal Med Assoc. 2009 Apr-Jun;48(174):103-6.
113. Misky GJ, Wald HL, Coleman EA. Post-hospitalization transitions: Examining the effects of timing of primary care provider follow-up. J Hosp Med. 2010 Sep;5(7):392-7. doi: 10.1002/jhm.666.
114. Moore C, Wisnivesky J, Williams S, McGinn T. Medical errors related to discontinuity of care from an inpatient to an outpatient setting. Gen Intern Med. 2003 Aug;18(8):646-51.
115. Moore P, Armitage G, Wright J, Dobrzanski S, Ansari N, Hammond I, Scally A. Medicines reconciliation using a shared electronic health care record. J Patient Saf. 2011 Sep;7(3):148-54. doi: 10.1097/PTS.0b013e31822c5bf9.
116. Moyer VA, Papile LA, Eichenwald E, Giardino AP, Khan MM, Singh H. An intervention to improve transitions from NICU to ambulatory care: quasi-experimental study. BMJ Qual Saf. 2014 Dec;23(12):e3.
117. Murphy EM, Oxencis CJ, Klauck JA, Meyer DA, Zimmerman JM. Medication reconciliation at an acADEsmic medical center: implementation of a comprehensive program from admission to discharge. Am J Health Syst Pharm. 2009 Dec 1;66(23):2126-31. doi: 10.2146/ajhp080552.
118. Musgrave CR, Pilch NA, Taber DJ, Meadows HB, McGillicuddy JW, Chavin KD, Baliga PK. Improving transplant patient safety through pharmacist discharge medication reconciliation. Am J Transplant. 2013 Mar;13(3):796-801. doi: 10.1111/ajt.12070. Epub 2013 Jan 17.
119. Nana B, Lee-Such S, Allen G. Initiation of an emergency department pharmacy program during economically challenging times. Am J Health Syst Pharm. 2012 Oct 1;69(19):1682-6.
120. Naylor MD. Comprehensive discharge planning for hospitalized elderly: a pilot study. Nurs Res. 1990 May-Jun;39(3):156-61.
121. Naylor M, Brooten D, Jones R, Lavizzo-Mourey R, Mezey M, Pauly M. Comprehensive discharge planning for the hospitalized elderly. A randomized clinical trial. Ann Intern Med. 1994 Jun 15;120(12):999-1006.
122. Naylor MD, Brooten D, Campbell R, Jacobsen BS, Mezey MD, Pauly MV, Schwartz JS. Comprehensive discharge planning and home follow-up of hospitalized elders: a randomized clinical trial. JAMA. 1999 Feb 17;281(7):613-20.
123. Naylor MD, McCauley KM. The effects of a discharge planning and home follow-up intervention on elders hospitalized with common medical and surgical cardiac conditions. J Cardiovasc Nurs. 1999 Oct;14(1):44-54.
124. Naylor MD, Brooten DA, Campbell RL, Maislin G, McCauley KM, Schwartz JS. Transitional care of older adults hospitalized with heart failure: a randomized, controlled trial. J Am Geriatr Soc. 2004 May;52(5):675-84. Erratum in: J Am Geriatr Soc. 2004 Jul;52(7):1228.
125. Naylor MD, Bowles KH, McCauley KM, Maccoy MC, Maislin G, Pauly MV, Krakauer R. High-value transitional care: translation of research into practice. J Eval Clin Pract. 2013 Oct;19(5):727-33. doi: 10.1111/j.1365-2753.2011.01659.x. Epub 2011 Mar 16.
126. Nuñez S, Hexdall A, Aguirre-Jaime A. Unscheduled returns to the emergency department: an outcome of medical errors? Qual Saf Health Care. 2006 Apr;15(2):102-8.
127. Nuño-Solinís R, Zabalegui IB, Rodríguez LS, Arce RS, Gagnon MP. Does interprofessional collaboration between care levels improve following the creation of an integrated delivery organisation? The Bidasoa case in the Basque Country. Int J Integr Care. 2013 Sep 20;13:e030. eCollection 2013.
128. Okoniewska BM, Santana MJ, Holroyd-Leduc J, Flemons W, O'Beirne M, White D, Clement F, Forster A, Ghali WA. The Seamless Transfer-of-Care Protocol: a randomized controlled trial assessing the efficacy of an electronic transfer-of-care communication tool. BMC Health Serv Res. 2012 Nov 21;12:414. doi: 10.1186/1472-6963-12-414.
129. Ong MK, Romano PS, Edgington S, Aronow HU, Auerbach AD, Black JT, De Marco T, Escarce JJ, Evangelista LS, Hanna B, Ganiats TG, Greenberg BH, Greenfield S, Kaplan SH, Kimchi A, Liu H, Lombardo D, Mangione CM, SADEsghi B, SADEsghi B, SarrafzADEsh M, Tong K, Fonarow GC; Better Effectiveness After Transition–Heart Failure (BEAT-HF) Research Group. Effectiveness of Remote Patient Monitoring After Discharge of Hospitalized Patients With Heart Failure: The Better Effectiveness After Transition -- Heart Failure (BEAT-HF) Randomized Clinical Trial. JAMA Intern Med. 2016 Mar;176(3):310-8. doi: 10.1001/jamainternmed.2015.7712.
130. Ouchida K, LoFaso VM, Capello CF, Ramsaroop S, Reid MC. Fast forward rounds: an effective method for teaching medical students to transition patients safely across care settings. J Am Geriatr Soc. 2009 May;57(5):910-7. doi: 10.1111/j.1532-5415.2009.02203.x. Epub 2009 Apr 2.
131. Parrish MM, O'Malley K, Adams RI, Adams SR, Coleman EA. Implementation of the care transitions intervention: sustainability and lessons learned. Prof Case Manag. 2009 Nov-Dec;14(6):282-93; quiz 294-5. doi: 10.1097/NCM.0b013e3181c3d380.
132. Paulino EI, Bouvy ML, Gastelurrutia MA, Guerreiro M, Buurma H; ESCP-SIR Rejkjavik Community Pharmacy Research Group. Drug related problems identified by European community pharmacists in patients discharged from hospital. Pharm World Sci. 2004 Dec;26(6):353-60.
133. Phatak A, Prusi R, Ward B, Hansen LO, Williams MV, Vetter E, Chapman N, Postelnick M. Impact of pharmacist involvement in the transitional care of high-risk patients through medication reconciliation, medication education, and post-discharge call-backs (IPITCH Study). J Hosp Med. 2016 Jan;11(1):39-44. doi: 10.1002/jhm.2493. Epub 2015 Oct 5.
134. Pourrat X, Roux C, Bouzige B, Garnier V, Develay A, Allenet B, Fraysse M, Halimi JM, Grassin J, Giraudeau B. Impact of drug reconciliation at discharge and communication between hospital and community pharmacists on drug-related problems: study protocol for a randomized controlled trial. Trials. 2014 Jun 30;15:260. doi: 10.1186/1745-6215-15-260.
135. Redmond P, Carroll H, Grimes T, Galvin R, McDonnell R, Boland F, McDowell R, Hughes C, Fahey T. GPs' and community pharmacists' opinions on medication management at transitions of care in Ireland. Fam Pract. 2016 Apr;33(2):172-8. doi: 10.1093/fampra/cmw006.
136. Reese PP, Hicks LS, McWilliams M, Britton O, McKean SCW. Hospitalist Home Visit Program Identifies Medication Errors in Vulnerable General Medicine Population. JCOM 2003 10(4).
137. Rice YB, Barnes CA, Rastogi R, Hillstrom TJ, Steinkeler CN. Tackling 30-Day, All-Cause Readmissions with a Patient-Centered Transitional Care Bundle. Popul Health Manag. 2016 Feb;19(1):56-62. doi: 10.1089/pop.2014.0163. Epub 2015 Apr 28.
138. Riordan CO, Delaney T, Grimes T. Exploring discharge prescribing errors and their propagation post-discharge: an observational study. Int J Clin Pharm. 2016 Oct;38(5):1172-81. doi: 10.1007/s11096-016-0349-7. Epub 2016 Jul 29.
139. Roorda C, Berendsen AJ, Haverkamp M, van der Meer K, de Bock GH. Discharge of breast cancer patients to primary care at the end of hospital follow-up: a cross-sectional survey. Eur J Cancer. 2013 May;49(8):1836-44. doi: 10.1016/j.ejca.2013.01.016. Epub 2013 Feb 28.
140. Roughead EE, Kalisch LM, Ramsay EN, Ryan P, Gilbert AL. Continuity of care: when do patients visit community healthcare providers after leaving hospital? Intern Med J. 2011 Sep;41(9):662-7. doi: 10.1111/j.1445-5994.2009.02105.x. Epub 2009 Oct 22.
141. Russell P, Hewage U, Thompson C. Method for improving the quality of discharge summaries written by a general medical team. Intern Med J. 2014 Mar;44(3):298-301. doi: 10.1111/imj.12362.
142. Santell JP. Reconciliation failures lead to medication errors. Jt Comm J Qual Patient Saf. 2006 Apr;32(4):225-9.
143. Scales DC, Fischer HD, Li P, Bierman AS, Fernandes O, Mamdani M, Rochon P, Urbach DR, Bell CM. Unintentional Continuation of Medications Intended for Acute Illness After Hospital Discharge: A Population-Based Cohort Study. J Gen Intern Med. 2016 Feb;31(2):196-202. doi: 10.1007/s11606-015-3501-5.
144. Schnipper JL, Kirwin JL, Cotugno MC, Wahlstrom SA, Brown BA, Tarvin E, Kachalia A, Horng M, Roy CL, McKean SC, Bates DW. Role of pharmacist counseling in preventing adverse drug events after hospitalization. Arch Intern Med. 2006 Mar 13;166(5):565-71.
145. Schnipper JL, Hamann C, Ndumele CD, Liang CL, Carty MG, Karson AS, Bhan I, Coley CM, Poon E, Turchin A, Labonville SA, Diedrichsen EK, Lipsitz S, Broverman CA, McCarthy P, Gandhi TK. Effect of an electronic medication reconciliation application and process redesign on potential adverse drug events: a cluster-randomized trial. Arch Intern Med. 2009 Apr 27;169(8):771-80. doi: 10.1001/archinternmed.2009.51.
146. Scotten M, Manos EL, Malicoat A, Paolo AM. Minding the gap: Interprofessional communication during inpatient and post-discharge chasm care. Patient Educ Couns. 2015 Jul;98(7):895-900. doi: 10.1016/j.pec.2015.03.009. Epub 2015 Mar 17.
147. Seidling HM, Schmitt SP, Bruckner T, Kaltschmidt J, Pruszydlo MG, Senger C, Bertsche T, Walter-Sack I, Haefeli WE. Patient-specific electronic decision support reduces prescription of excessive doses. Qual Saf Health Care. 2010 Oct;19(5):e15. doi: 10.1136/qshc.2009.033175. Epub 2010 Apr 27.
148. Sharma AN, Dvorkin R, Tucker V, Margulies J, Yens D, Rosalia A Jr. Medical reconciliation in patients discharged from the emergency department. J Emerg Med. 2012 Aug;43(2):366-73. doi: 10.1016/j.jemermed.2011.05.080. Epub 2011 Dec 22.
149. Shastri A, Bangar S, Waldman S, Esfahani E, Brindle N. Content and Timing of Inpatient Discharge Summaries at the Mount. BJMP 2014;7(3):a726
150. Shen MW, Hershey D, Bergert L, Mallory L, Fisher ES, Cooperberg D. Pediatric hospitalists collaborate to improve timeliness of discharge communication. Hosp Pediatr. 2013 Jul;3(3):258-65.
151. Shu CC, Hsu NC, Lin YF, Wang JY, Lin JW, Ko WJ. Integrated post-discharge transitional care in a hospitalist system to improve discharge outcome: an experimental study. BMC Med. 2011 Aug 17;9:96. doi: 10.1186/1741-7015-9-96.
152. Sides EG, Zimmer LO, Wilson L, Pan W, Olson DM, Peterson ED, Bushnell C. Medication coaching program for patients with minor stroke or TIA: a pilot study. BMC Public Health. 2012 Jul 25;12:549. doi: 10.1186/1471-2458-12-549.
153. Silva PA, Bernstam EV, Markowitz E, Johnson TR, Zhang J, Herskovic JR. Automated medication reconciliation and complexity of care transitions. AMIA Annu Symp Proc. 2011;2011:1252-60. Epub 2011 Oct 22.
154. Singh H, Khan R, Giardina TD, Paul LW, Daci K, Gould M, El-Serag H. Postreferral colonoscopy delays in diagnosis of colorectal cancer: a mixed-methods analysis. Qual Manag Health Care. 2012 Oct-Dec;21(4):252-61.
155. Smith L, Mosley J, Lott S, Cyr E Jr, Amin R, Everton E, Islami A, Phan L, Komolafe O. Impact of pharmacy-led medication reconciliation on medication errors during transition in the hospital setting. Pharm Pract (Granada). 2015 Oct-Dec;13(4):634. doi: 10.18549/PharmPract.2015.04.634. Epub 2015 Dec 15.
156. Soong C, Kurabi B, Wells D, Caines L, Morgan MW, Ramsden R, Bell CM. Do post-discharge phone calls improve care transitions? A cluster-randomized trial. PLoS One. 2014 Nov 11;9(11):e112230. doi: 10.1371/journal.pone.0112230. eCollection 2014.
157. Spalek VH, Gong WC. Pharmaceutical care in an integrated health system. J Am Pharm Assoc (Wash). 1999 Jul-Aug;39(4):553-7.
158. Spehar AM, Campbell RR, Cherrie C, Palacios P, Scott D, Baker JL, Bjornstad B, Wolfson J. Seamless Care: Safe Patient Transitions from Hospital to Home. Henriksen K, Battles JB, Marks ES, Lewin DI, editors. Advances in Patient Safety: From Research to Implementation (Volume 1: Research Findings). Rockville (MD): Agency for Healthcare Research and Quality (US); 2005 Feb.
159. Stafford L, Peterson GM, Bereznicki LR, Jackson SL, van Tienen EC, Angley MT, Bajorek BV, McLachlan AJ, Mullan JR, Misan GM, Gaetani L. Clinical outcomes of a collaborative, home-based post-discharge warfarin management service. Ann Pharmacother. 2011 Mar;45(3):325-34. doi: 10.1345/aph.1P617. Epub 2011 Mar 8.
160. Staveski SL, Parveen VP, Madathil SB, Kools S, Franck LS. Nurse and parent perceptions associated with the Parent Education Discharge Instruction Programme in southern India. Cardiol Young. 2016 Aug;26(6):1168-75. doi: 10.1017/S1047951115002164. Epub 2015 Oct 12.
161. Stein R, Neufeld D, Shwartz I, Erez I, Haas I, Magen A, Glassberg E, Shmulevsky P, Paran H. Assessment of surgical discharge summaries and evaluation of a new quality improvement model. Isr Med Assoc J. 2014 Nov;16(11):714-7.
162. Stella SA, Keniston A, Frank MG, Heppe D, Mastalerz K, Lones J, Brody D, Albert RK, Burden M. Post-discharge telephone calls by hospitalists as a transitional care strategy. Am J Manag Care. 2016 Oct 1;22(10):e338-e342.
163. Steurbaut S, Leemans L, Leysen T, De Baere E, Cornu P, Mets T, Dupont AG. Medication history reconciliation by clinical pharmacists in elderly inpatients admitted from home or a nursing home. Ann Pharmacother. 2010 Oct;44(10):1596-603. doi: 10.1345/aph.1P192. Epub 2010 Aug 24.
164. Stone BL, Boehme S, Mundorff MB, Maloney CG, Srivastava R. Hospital admission medication reconciliation in medically complex children: an observational study. Arch Dis Child. 2010 Apr;95(4):250-5. doi: 10.1136/adc.2009.167528. Epub 2009 Nov 30.
165. Stuffken R, Heerdink ER, de Koning FH, Souverein PC, Egberts AC. Association between hospitalization and discontinuity of medication therapy used in the community setting in the Netherlands. Ann Pharmacother. 2008 Jul;42(7):933-9. doi: 10.1345/aph.1L062. Epub 2008 Jun 10.
166. Tamblyn R, Huang AR, Meguerditchian AN, WinslADEs NE, Rochefort C, Forster A, Eguale T, Buckeridge D, Jacques A, Naicker K, Reidel KE. Using novel Canadian resources to improve medication reconciliation at discharge: study protocol for a randomized controlled trial. Trials. 2012 Aug 27;13:150. doi: 10.1186/1745-6215-13-150.
167. Tan B, Mulo B, Skinner M. Transition from hospital to primary care: an audit of discharge summary - medication changes and follow-up expectations. Intern Med J. 2014 Nov;44(11):1124-7. doi: 10.1111/imj.12581.
168. Tan B, Mulo B, Skinner M. Discharge documentation improvement project: a pilot study. Intern Med J. 2015 Dec;45(12):1280-5. doi: 10.1111/imj.12895.
169. Triller DM, Clause SL, Hamilton RA. Risk of adverse drug events by patient destination after hospital discharge. Am J Health Syst Pharm. 2005 Sep 15;62(18):1883-9.
170. Tsilimingras D, Schnipper J, Duke A, Agens J, Quintero S, Bellamy G, Janisse J, Helmkamp L, Bates DW. Post-Discharge Adverse Events Among Urban and Rural Patients of an Urban Community Hospital: A Prospective Cohort Study. J Gen Intern Med. 2015 Aug;30(8):1164-71. doi: 10.1007/s11606-015-3260-3. Epub 2015 Mar 31.
171. Uijen AA, Schers HJ, Schene AH, Schellevis FG, Lucassen P, van den Bosch WJ. Experienced continuity of care in patients at risk for depression in primary care. Eur J Gen Pract. 2014 Sep;20(3):161-6. doi: 10.3109/13814788.2013.828201. Epub 2013 Sep 13.
172. Uitvlugt EB, Siegert CE, Janssen MJ, Nijpels G, Karapinar-Çarkit F. Completeness of medication-related information in discharge letters and post-discharge general practitioner overviews. Int J Clin Pharm. 2015 Dec;37(6):1206-12. doi: 10.1007/s11096-015-0187-z. Epub 2015 Sep 4.
173. Varkey P, Cunningham J, O'Meara J, Bonacci R, Desai N, Sheeler R. Multidisciplinary approach to inpatient medication reconciliation in an acADEsmic setting. Am J Health Syst Pharm. 2007 Apr 15;64(8):850-4.
174. van Melle MA, Erkelens DC, van Stel HF, de Wit NJ, Zwart DL. Pilot study on identification of incidents in healthcare transitions and concordance between medical records and patient interview data. BMJ Open. 2016 Aug 19;6(8):e011368. doi: 10.1136/bmjopen-2016-011368.
175. Villani A, Malfatto G, Compare A, Della Rosa F, Bellardita L, Branzi G, Molinari E, Parati G. Clinical and psychological telemonitoring and telecare of high risk heart failure patients. J Telemed Telecare. 2014 Dec;20(8):468-75. doi: 10.1177/1357633X14555644. Epub 2014 Oct 22.
176. Vira T, Colquhoun M, Etchells E. Reconcilable differences: correcting medication errors at hospital admission and discharge. Qual Saf Health Care. 2006 Apr;15(2):122-6.
177. Vrijhoef HJ, Diederiks JP, Spreeuwenberg C, Wolffenbuttel BH. Substitution model with central role for nurse specialist is justified in the care for stable type 2 diabetic outpatients. J Adv Nurs. 2001 Nov;36(4):546-55.
178. Waring ME, McManus RH, Saczynski JS, Anatchkova MD, McManus DD, Devereaux RS, Goldberg RJ, Allison JJ, Kiefe CI; TRACE-CORE Investigators. Transitions, Risks, and Actions in Coronary Events--Center for Outcomes Research and Education (TRACE-CORE): design and rationale. Circ Cardiovasc Qual Outcomes. 2012 Sep 1;5(5):e44-50.
179. Warsame R, Kasi PM, Villasboas-Bisneto JC, Gallenberg D, Wolf R, Ward J, Matt-Hensrud N, Grethen K, Colborn L, Zeldenrust S, Lacy MQ, Thompson CA. Transition of Care for Inpatient Hematology Patients Receiving Chemotherapy: Development of Hospital Discharge Huddle Process and Effects of Implementation. J Oncol Pract. 2016 Jan;12(1):e88-94. doi: 10.1200/JOP.2015.005785. Epub 2015 Sep 15.
180. Watkins L, Hall C, Kring D. Hospital to home: a transition program for frail older adults. Prof Case Manag. 2012 May-Jun;17(3):117-23; quiz 124-5. doi: 10.1097/NCM.0b013e318243d6a7.
181. Weiner M, Perkins AJ, Callahan CM. Errors in completion of referrals among older urban adults in ambulatory care. J Eval Clin Pract. 2010 Feb;16(1):76-81. doi: 10.1111/j.1365-2753.2008.01117.x.
182. Were MC, Li X, Kesterson J, CadwallADEsr J, Asirwa C, Khan B, Rosenman MB. ADEsquacy of hospital discharge summaries in documenting tests with pending results and outpatient follow-up providers. J Gen Intern Med. 2009 Sep;24(9):1002-6. doi: 10.1007/s11606-009-1057-y. Epub 2009 Jul 3.
183. Williams H, Edwards A, Hibbert P, Rees P, Prosser Evans H, Panesar S, Carter B, Parry G, Makeham M, Jones A, Avery A, Sheikh A, Donaldson L, Carson-Stevens A. Harms from discharge to primary care: mixed methods analysis of incident reports. Br J Gen Pract. 2015 Dec;65(641):e829-37. doi: 10.3399/bjgp15X687877.
184. Wilson S, Ruscoe W, Chapman M, Miller R. General practitioner-hospital communications: a review of discharge summaries. J Qual Clin Pract. 2001 Dec;21(4):104-8.
185. Witherington EM, Pirzada OM, Avery AJ. Communication gaps and readmissions to hospital for patients aged 75 years and older: observational study. Qual Saf Health Care. 2008 Feb;17(1):71-5. doi: 10.1136/qshc.2006.020842.
186. Wong JD, Bajcar JM, Wong GG, Alibhai SM, Huh JH, Cesta A, Pond GR, Fernandes OA. Medication reconciliation at hospital discharge: evaluating discrepancies. Ann Pharmacother. 2008 Oct;42(10):1373-9. doi: 10.1345/aph.1L190.
187. Wortman SB. Medication reconciliation in a community, nonteaching hospital. Am J Health Syst Pharm. 2008 Nov 1;65(21):2047-54. doi: 10.2146/ajhp080091.
188. Yi SB, Shan JC, Hong GL. Medication reconciliation service in Tan Tock Seng Hospital. Int J Health Care Qual Assur. 2013;26(1):31-6.
189. Young JM, Butow PN, Walsh J, Durcinoska I, Dobbins TA, Rodwell L, Harrison JD, White K, Gilmore A, Hodge B, Hicks H, Smith S, O'Connor G, Byrne CM, Meagher AP, Jancewicz S, Sutherland A, Ctercteko G, Pathma-Nathan N, Curtin A, Townend D, Abraham NS, Longfield G, Rangiah D, Young CJ, Eyers A, Lee P, Fisher D, Solomon MJ. Multicenter randomized trial of centralized nurse-led telephone-based care coordination to improve outcomes after surgical resection for colorectal cancer: the CONNECT intervention. J Clin Oncol. 2013 Oct 1;31(28):3585-91. doi: 10.1200/JCO.2012.48.1036. Epub 2013 Sep 3.
190. Zhang J, Burridge L, Baxter KA, Donald M, Foster MM, Hollingworth SA, Ware RS, Russell AW, Jackson CL. A new model of integrated primary-secondary care for complex diabetes in the community: study protocol for a randomised controlled trial. Trials. 2013 Nov 12;14:382. doi: 10.1186/1745-6215-14-382.
191. Zoni AC, Durán García ME, Jiménez Muñoz AB, Salomón Pérez R, Martin P, Herranz Alonso A. The impact of medication reconciliation program at admission in an internal medicine department. Eur J Intern Med. 2012 Dec;23(8):696-700. doi: 10.1016/j.ejim.2012.08.013. Epub 2012 Sep 27.
